# Global, regional and national burden of Metabolic dysfunction-associated steatotic liver disease in adolescents and adults aged 15–49 years from 1990 to 2021: results from the 2021 Global Burden of Disease study

**DOI:** 10.3389/fmed.2025.1568211

**Published:** 2025-06-25

**Authors:** Biwu Xu, Hailong Li, Yijie Pi, Kaiyuan Li

**Affiliations:** ^1^Department of Hepatobiliary Surgery, Taizhou Hospital of Zhejiang Province affiliated to Wenzhou Medical University, Linhai, Zhejiang, China; ^2^Department of Cardiovascular Medicine, The Second Affiliated Hospital, Jiangxi Medical College, Nanchang University, Nanchang, Jiangxi, China; ^3^Department of Ophthalmology, The Second Affiliated Hospital, Jiangxi Medical College, Nanchang University, Nanchang, Jiangxi, China; ^4^Graduate School of Dalian Medical University, Dalian Medical University, Dalian, China

**Keywords:** Metabolic dysfunction-associated steatotic liver disease, DALYs, prevalence trends, socioeconomic disparities, GBD 2021

## Abstract

**Background:**

With rising obesity and diabetes rates, the incidence of Metabolic dysfunction-associated steatotic liver disease (MASLD) among individuals aged 15–49 is increasing, affecting their productivity, health, and quality of life. However, a comprehensive global assessment of MASLD burden and long-term trends in this age group remains lacking.

**Methods:**

The study analyzed prevalence, incidence, mortality, and disability-adjusted life years (DALYs) for individuals aged 15–49 using data from the Global Burden of Disease (GBD) 2021 study. Trends from 1990 to 2021 were assessed using Estimated Annual Percentage Change (EAPC), with data stratified by Socio-Demographic Index (SDI), sex, and age.

**Results:**

Between 1990 and 2021, the number of individuals with MASLD increased from 343 to 666 million, reflecting an average annual growth rate of 0.95%. The number of MASLD-related deaths increased from 7,920 to 15,110, corresponding to an average annual growth rate of 0.8%. Over the same period, DALYs climbed from 399,000 to 751,000, indicating an increase of approximately 0.77%. Low-SDI regions showed the fastest growth in mortality and DALYs, whereas some high-SDI areas, notably high-income Asia Pacific, demonstrated a decline in these measures. Regarding sex-based differences, males exhibited a higher growth rate of MASLD-related deaths and DALYs compared to females, although the increase in prevalence was more pronounced among females. Age-group analysis revealed that the 45–49 years group experienced the most significant surge in MASLD burden.

**Conclusion:**

Over the past three decades, the global burden of MASLD has risen significantly, especially in regions with rapidly increasing obesity rates. Despite progress in some high-income countries, the persistent escalation in low-income regions underscores the urgency for targeted interventions.

## 1 Introduction

Metabolic dysfunction-associated steatotic liver disease (MASLD), previously known as non-alcoholic fatty liver disease (NAFLD) (1), is one of the most prevalent chronic liver diseases worldwide, with an estimated overall prevalence of approximately 30%, and it imposes a substantial health and economic burden (2–4). The clinical spectrum of MASLD spans simple steatosis, metabolic dysfunction-associated steatohepatitis (MASH) through cirrhosis and hepatocellular carcinoma and is strongly associated with multi-systemic complications such as type 2 diabetes mellitus, cardiovascular disease and chronic kidney disease (5, 6).

The global prevalence of MASLD continues to rise, particularly in nations with high rates of obesity, diabetes, and metabolic syndrome. High-income countries have demonstrated a higher prevalence due to dietary patterns and lifestyle factors (4), whereas low- and middle-income countries are experiencing a rapid increase in MASLD attributed to urbanization and the westernization of diets (7, 8). MASLD is closely associated with a range of systemic diseases, and it notably undermines health-related quality of life while escalating healthcare costs and economic burdens (9–12). Large population cohorts now confirm that MASLD clusters with—and may precede—cardiovascular events at the family level (13).

This study specifically focuses on individuals aged 15–49 years, a demographically and socioeconomically critical group that is often underrepresented in disease burden research. This age group typically constitutes the most active segment of the labor force and plays a central role in economic productivity, family development, and social stability. In addition to diminishing work capacity and contributing to increased sick leave and premature mortality, MASLD may also negatively impact reproductive health in women (14–16). Moreover, the earlier onset of MASLD during these years increases the lifetime risk of progression to cirrhosis and hepatocellular carcinoma, potentially leading to a greater cumulative health and economic impact (17, 18). However, current assessments of MASLD burden in this specific age group remain limited.

The Global Burden of Disease (GBD) study, encompassing data from 195 countries and regions, serves as a key instrument for investigating the epidemiological characteristics of MASLD. The latest iteration of GBD 2021 has further refined disease estimation and analytical methodologies (19, 20). The aim of this study was to systematically estimate the prevalence, age-standardized incidence and disability-adjusted life years (DALYs) of MASLD using the GBD 2021 database, and to compare the spatial and temporal evolution of MASLD by Socio-demographic Index (SDI) stratification from 1990 to 2021, to provide an evidence-based basis for the development of clinical management and health policies. In order to be in line with the latest international consensus, the codes for “non-alcoholic fatty liver disease” in GBD 2021 are collectively referred to as “MASLD.”

## 2 Materials and methods

### 2.1 Data source and disease definition

In GBD 2021, the condition is coded as “non-alcoholic fatty liver disease.” In line with the 2023 international consensus that renamed NAFLD to MASLD, we henceforth refer to this code as MASLD. GBD 2021 provides detailed estimates of epidemiological burden from 1990 through 2021 for 21 GBD regions and 204 countries and territories, encompassing more than 369 diseases and injuries (21). The dataset is freely accessible from the Global Health Data Exchange website^1^, and the relevant statistical models and methodological details have been extensively documented in prior reports (22).

### 2.2 SDI

The Institute for Health Metrics and Evaluation (IHME) introduced the SDI in 2015 to quantify the level of socioeconomic development across countries or regions and to investigate its relationship with health outcomes. The SDI ranges from values close to zero (indicating lower levels of socioeconomic development) to values near the upper limit (reflecting higher levels of socioeconomic development). Based on this index, countries and regions are stratified into five development tiers, spanning from low to high socioeconomic development. This stratification facilitates comparative analyses of health burdens at various stages of socioeconomic advancement, thereby offering a foundation for formulating evidence-based public health policies and interventions.

### 2.3 DALYs

DALYs to quantify the global health burden of MASLD among adolescents and adults. DALYs represent the sum of years of life lost (YLL) due to premature mortality and years lived with disability (YLD) attributable to the disease. In GBD 2021, YLL and YLD estimates are derived from extensive and high-quality population data.

### 2.4 Estimated annual percentage change (EAPC)

EAPC is a widely used metric to measure the average yearly variation in specific health indicators (e.g., prevalence, incidence) over a defined period. In this study, EAPC was employed to examine the dynamics of MASLD prevalence, incidence, and DALYs from 1990 to 2021 among adolescents and adults.

Estimated annual percentage change is calculated based on a linear regression model that regresses the natural logarithm of the health indicator on the calendar year. Specifically, the model is expressed as: ln(y) = α + βx + ϵ, where y is the observed indicator, x denotes the year, α is the intercept, β is the slope, and ϵ represents the random error term. The EAPC value is derived from the slope β, and its 95% confidence interval (CI) is determined from the model output. A lower bound of the 95% CI exceeding 0 indicates a statistically significant upward trend; an upper bound below 0 signifies a statistically significant downward trend; and a CI that includes 0 suggests that the change is not statistically significant.

### 2.5 Data analysis

All analyses and data visualization were conducted using R software (version 4.3.0), with statistical significance assessed at a two-sided *p*-value < 0.05. The ggplot2 package was used to generate figures and graphs.

### 2.6 Ethics statement

The parent Global Burden of Disease 2021 study received approval from the Institutional Review Board of the University of Washington, Seattle, United States (UW IRB approval number STUDY00009060). The data released by GBD are fully de-identified and aggregated. Because the present work is a secondary analysis of those public, anonymized data, our own institution deemed it non-human-subjects research and waived additional review and informed consent.

## 3 Results

### 3.1 Global level

Between 1990 and 2021, the epidemiological characteristics of MASLD among adolescents and adults changed substantially. The global number of MASLD cases rose from approximately 342.94 million (95% Uncertainty Interval, UI: 305.23–388.80 million) in 1990 to 665.77 million (95% UI: 596.51–754.28 million) in 2021, representing a 94% increase. Meanwhile, the global prevalence increased from 12652.46 per 100,000 population (95% UI: 11261.11–14344.35) to 16860.94 per 100,000 (95% UI: 15106.92–19102.52), indicating a continuous upward trend (Table 1).

**TABLE 1:** The prevalent cases and prevalence rates for MASLD among the adolescents and adults aged 15–49 years from 1990 to 2021.

	Prevalent cases			Prevalence rates		
Location	1990 million (95%UI)	2021 million (95%UI)	Percentage change (%)	1990 per 100,000 (95% UI)	2021 per 100,000 (95% UI)	EAPC (95% CI)
Global	342.94 (305.23–388.8)	665.77 (596.51–754.28)	0.94	12652.46 (11261.11–14344.35)	16860.94 (15106.92–19102.52)	0.95 (0.88–1.02)
High SDI	44.5 (39.62–50.35)	71.5 (63.92–81.01)	0.61	9656.52 (8597.73–10925.33)	14237.03 (12726.7–16129.17)	1.35 (1.31–1.39)
High-middle SDI	73.05 (64.99–82.95)	116.04 (103.31–132.08)	0.59	12942.38 (11514.62–14697.06)	18432.23 (16409–20979.04)	1.16 (1.02–1.3)
Middle SDI	123.77 (109.84–140.46)	233.97 (209.77–265.71)	0.89	13592.57 (12061.92–15425.05)	18642.26 (16713.97–21170.64)	1.04 (0.97–1.12)
Low-middle SDI	74.4 (65.93–84.03)	168.96 (151.25–190.38)	1.27	13499.83 (11964.24–15247.71)	16625.4 (14883.45–18733.27)	0.67 (0.62–0.73)
Low SDI	26.9 (23.83–30.3)	74.77 (66.25–84.79)	1.78	12169.65 (10781.77–13707.42)	13784.9 (12214.02–15633.8)	0.41 (0.37–0.46)
Andean Latin America	2.27 (2.02–2.57)	5.42 (4.81–6.16)	1.39	12195.19 (10817.13–13773.94)	15485.47 (13763.61–17611.66)	0.83 (0.81–0.84)
Australasia	0.89 (0.79–1.01)	1.58 (1.4–1.79)	0.78	8262.99 (7285.72–9383.73)	10950.29 (9697.86–12421.18)	0.88 (0.84–0.93)
Caribbean	2.46 (2.19–2.78)	3.87 (3.45–4.39)	0.57	13442.83 (11982.62–15204.09)	16158.17 (14428.25–18335.3)	0.6 (0.57–0.64)
Central Asia	4.66 (4.12–5.3)	8.7 (7.77–9.91)	0.87	13984.21 (12343.47–15884.89)	17851.53 (15934.14–20324.76)	0.74 (0.68–0.8)
Central Europe	7.69 (6.84–8.75)	7.96 (7.06–9.03)	0.04	12386.77 (11020.08–14092.75)	15105.79 (13405.04–17139.38)	0.68 (0.63–0.73)
Central Latin America	11.5 (10.23–13)	24.08 (21.51–27.33)	1.09	14089.35 (12527.24–15930.19)	18090.63 (16154.79–20527.55)	0.82 (0.81–0.83)
Central Sub-Saharan Africa	2.6 (2.29–2.98)	7.73 (6.83–8.79)	1.97	10647.09 (9386.91–12184.72)	11862.37 (10470.69–13475.82)	0.35 (0.32–0.37)
East Asia	87.65 (77.64–99.71)	126.74 (111.96–145.52)	0.45	12723.93 (11271.53–14474.72)	18407.97 (16262.02–21135.4)	1.21 (0.95–1.46)
Eastern Europe	12.8 (11.31–14.65)	13.53 (11.89–15.36)	0.06	11603.22 (10251.73–13282.62)	14055.59 (12352.35–15960.12)	0.56 (0.5–0.62)
Eastern Sub-Saharan Africa	9.46 (8.39–10.65)	27.07 (23.92–30.74)	1.86	11337.18 (10053.65–12773.13)	12925.25 (11424.2–14679.1)	0.42 (0.38–0.46)
High-income Asia Pacific	8.06 (7.17–9.09)	8.38 (7.41–9.48)	0.04	8685.28 (7725.43–9790.15)	10719.25 (9477.3–12113.65)	0.83 (0.76–0.9)
High-income North America	12.79 (11.31–14.51)	18.82 (16.73–21.32)	0.47	8579.06 (7590.96–9737.07)	11159.33 (9916.89–12641.46)	0.91 (0.85–0.97)
North Africa and Middle East	35.92 (32.09–40.46)	106.41 (95.68–119.81)	1.96	22409.35 (20022.77–25244.92)	31829.13 (28618.37–35834.85)	1.23 (1.19–1.28)
Oceania	0.4 (0.36–0.46)	1.03 (0.92–1.18)	1.57	12627.38 (11210.63–14361.04)	14587.58 (12978.4–16626.35)	0.49 (0.45–0.52)
South Asia	65.45 (58.01–73.99)	149.73 (133.05–169.02)	1.29	12371.6 (10965.32–13986.5)	14873.36 (13216.15–16789.56)	0.57 (0.47–0.67)
Southeast Asia	32.02 (28.39–36.34)	62.9 (56.19–71.34)	0.96	13531.97 (11998.06–15359.3)	16962.85 (15154.1–19238.95)	0.76 (0.75–0.78)
Southern Latin America	2.08 (1.84–2.36)	3.95 (3.49–4.47)	0.9	8486.69 (7495.18–9653.92)	11377.24 (10072.83–12891.81)	0.95 (0.92–0.98)
Southern Sub-Saharan Africa	3.51 (3.12–3.97)	7.46 (6.64–8.47)	1.13	13639.95 (12099.52–15424.85)	17288.69 (15380.12–19609.5)	0.75 (0.7–0.81)
Tropical Latin America	11.51 (10.2–13.16)	21.92 (19.55–25.03)	0.9	14660.31 (12995.36–16759.61)	18291.15 (16318.74–20885.4)	0.76 (0.74–0.79)
Western Europe	17.78 (15.89–20.1)	24.15 (21.56–27.37)	0.36	9193.24 (8215.11–10393.99)	12811.28 (11435.23–14518.36)	1.12 (1.05–1.19)
Western Sub-Saharan Africa	11.45 (10.13–12.99)	34.33 (30.45–38.95)	2	13371.1 (11836.99–15170.94)	14969.8 (13278.16–16985.21)	0.36 (0.35–0.37)

Regarding incidence, the number of new cases increased from 19.82 million (95% UI: 17.41–22.36 million) in 1990 to 35.60 million (95% UI: 31.68–39.97 million) in 2021, an 80% increase (Supplementary Table 1). The incidence rate also climbed from 731.07 per 100,000 (95% UI: 642.35–825.09) to 901.60 per 100,000 (95% UI: 802.21–1012.22), reflecting increases in both the absolute number of new cases and the incidence rate.

Globally, MASLD-related DALYs grew significantly from 0.40 million (95% UI: 0.26–0.61 million) in 1990 to 0.75 million (95% UI: 0.48–1.12 million) in 2021, an 88% increase (Supplementary Table 2). The DALY rate similarly rose from 14.72 per 100,000 (95% UI: 9.61–22.54) to 19.01 per 100,000 (95% UI: 12.05–28.47), indicating an escalating disease burden.

In terms of mortality, the global number of MASLD-related deaths rose from 7.92 thousand (95% UI: 5.13–12.46 thousand) in 1990 to 15.11 thousand (95% UI: 9.52–22.60 thousand) in 2021, a 91% increase; correspondingly, the mortality rate increased from 0.29 per 100,000 (95% UI: 0.19–0.46) to 0.38 per 100,000 (95% UI: 0.24–0.57), underscoring MASLD’s intensifying impact on mortality (Supplementary Table 3).

Estimated annual percentage change analysis revealed that the average annual growth rate of MASLD cases was 0.95% (95% CI: 0.88–1.02), while MASLD-related deaths grew annually by 0.8% (95% CI: 0.63–0.97). Incidence demonstrated an EAPC of 0.72% (95% CI: 0.67–0.77), and DALYs of 0.77% (95% CI: 0.60–0.93). Although the growth rates for incidence and mortality were relatively moderate, the global numbers of MASLD cases, deaths, and overall disease burden all rose steadily, particularly the marked DALY increase—highlighting MASLD’s persistent threat to global health.

### 3.2 SDI regional level

**FIGURE 1:**
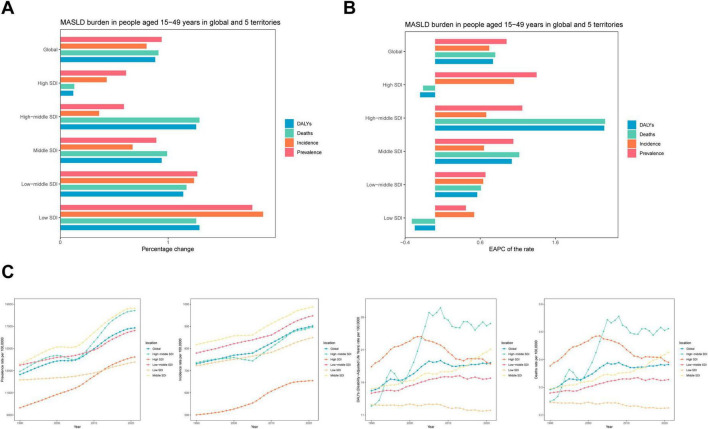
Temporal trend of MASLD burden among the adolescents and adults aged 15–49 years in global and five territories. **(A)** Percentage change in cases of prevalent, incident, DALYs and Deaths in 1990 and 2021. **(B)** The EAPC of prevalence, incidence, DALYs and Deaths rates from 1990 to 2021. **(C)** The rates of prevalence, incidence, DALYs and Deaths from 1990 to 2021.

Over the past three decades, the MASLD burden among adolescents and adults exhibited notable regional disparities across different SDI tiers (Figure 1 and Table 1). With respect to prevalence, low-SDI regions experienced an increase in MASLD cases from 26.9 million (95% UI: 23.83–30.30 million) in 1990 to 74.77 million (95% UI: 66.25–84.79 million) in 2021, representing a 178% increase and an EAPC of 0.41% (95% CI: 0.37–0.46). High-SDI regions also showed substantial growth, with MASLD cases rising from 44.50 million (95% UI: 39.62–50.35 million) to 71.5 million (95% UI: 63.92–81.01 million)—a 61% increase—and an EAPC of 1.35% (95% CI: 1.31–1.39), demonstrating continued growth in MASLD prevalence. In terms of mortality, MASLD-related deaths in low-SDI regions increased significantly from 27.77 (95% UI: 17.77–43.06) to 63.48 (95% UI: 40.25–95.97), indicating a substantial rise.

For incidence, middle-SDI regions exhibited a rapid increase, with new MASLD cases growing from 7.44 million (95% UI: 6.54–8.39 million) in 1990 to 12.41 million (95% UI: 11.07–13.97 million) in 2021, a 67% increase (EAPC: 0.65%, 95% CI: 0.60–0.70). By contrast, high-SDI regions experienced a −20% decrease in DALYs (EAPC: −0.2, 95% CI: −0.45 to 0.05), suggesting some success in mitigating the MASLD burden. While rising SDI values generally corresponded to more moderate growth in MASLD prevalence and the overall burden, the absolute burden in high-SDI regions remained substantial. Conversely, low-SDI regions exhibited faster overall increases, particularly for MASLD-related deaths and DALYs.

### 3.3 GBD regional level

**FIGURE 2:**
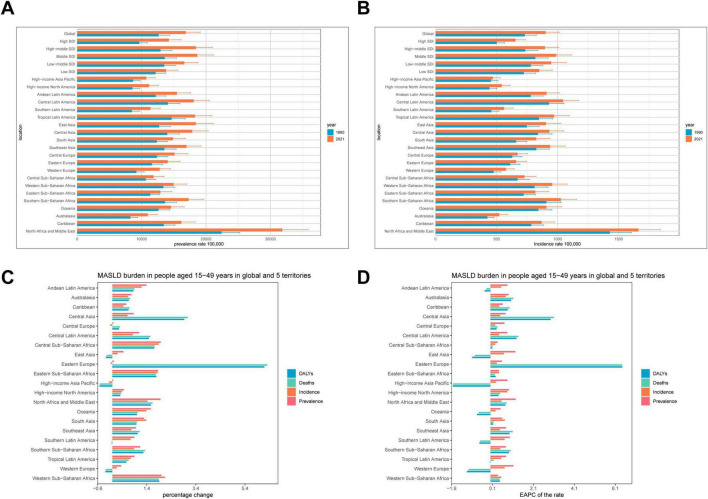
Temporal trend of MASLD burden among the adolescents and adults aged 15–49 years in regions. **(A)** Prevalence rate per 100,000 population in 1990 and 2021. **(B)** Incidence rate per 100,000 population in 1990 and 2021. **(C)** Percentage change in cases of prevalence, incidence, deaths and DALYs in 1990 and 2021. **(D)** EAPC of rates of prevalent, incident, deaths and DALYs from 1990 to 2021.

Between 1990 and 2021, MASLD burden and trends among adolescents and adults displayed pronounced heterogeneity across different GBD regions (Figure 2 and Table 1). In East Asia, MASLD cases expanded from 2.85 million (95% UI: 2.20–3.81 million) in 1990 to 9.88 million (95% UI: 7.65–13.06 million) in 2021, a 247% increase, indicating a marked upward trajectory. Western Europe also showed an increase, from 4.60 million (95% UI: 3.57–5.97 million) to 7.96 million (95% UI: 6.63–9.55 million), a 73% growth, reflecting a continued rise in MASLD burden.

Eastern Europe was especially notable. Although MASLD cases grew from 1.44 million (95% UI: 1.09–1.89 million) to 2.15 million (95% UI: 1.64–2.83 million), a 49% increase, MASLD-related deaths rose substantially from 292.43 (95% UI: 180.54–455.67) to 2,137.26 (95% UI: 1277.05–3367.80), a 631% rise, with an EAPC of 6.43% (95% CI: 5.23–7.64), signaling a marked rise MASLD’s health impact. Central Asia likewise exhibited a clear upward trendin new cases, from 279.76 thousand (95% UI: 245.26–318.09 thousand) to 454.27 thousand (95% UI: 401.46–511.36 thousand), a 62% increase (EAPC: 0.47%, 95% CI: 0.41–0.53). Simultaneously, DALYs increased markedly from 6.70 thousand (95% UI: 4.26–10.30 thousand) to 26.34 thousand (95% UI: 15.49–41.18 thousand), a 293% increase (EAPC: 2.94%, 95% CI: 2.46–3.42).

Disability-adjusted life years in South Asia and Southeast Asia increased by 72% and 43%, respectively, further underscoring a rising MASLD burden. In contrast, high-income Asia Pacific showed a downward trend in MASLD-related deaths and DALYs: deaths declined from 164.20 (95% UI: 95.32–268.06) to 80.58 (95% UI: 45.92–136.70), an 51% decrease (EAPC: −1.82%, 95% CI: −1.98 to −1.66), while DALYs dropped from 7.92 thousand (95% UI: 4.68–12.64 thousand) to 3.84 thousand (95% UI: 2.21–6.41 thousand) (EAPC: −0.65%, 95% CI: −0.78 to −0.52). Low- and middle-income GBD regions experienced steeper, more substantial increases in MASLD burden compared to high-income regions, most notably in Eastern Europe and Central Asia, where mortality and DALYs rose precipitously. Although MASLD burden also rose in high-income regions, the growth was relatively moderate, with some metrics showing a decline.

### 3.4 Countries level

**FIGURE 3:**
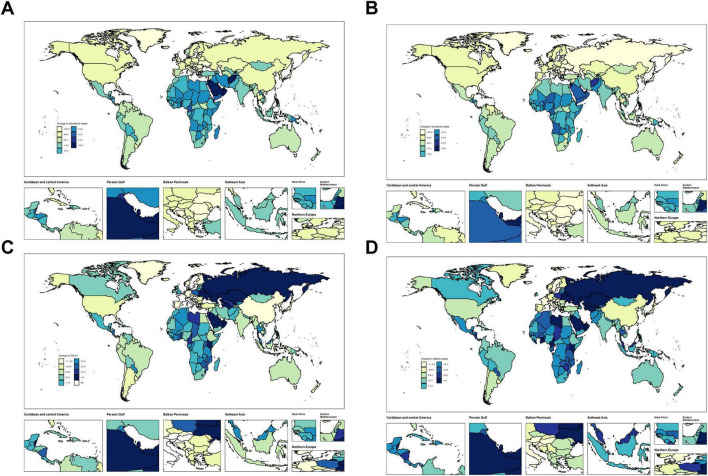
Temporal trend of MASLD burden among the adolescents and adults aged 15–49 years globally. **(A)** Change in prevalence cases across 204 countries in 1990 and 2021. **(B)** Change in incidence cases across 204 countries in 1990 and 2021. **(C)** Change in DALYs cases across 204 countries in 1990 and 2021. **(D)** Change in deaths cases across 204 countries in 1990 and 2021.

Across individual countries, MASLD burden among adolescents and adults exhibited a general rise in prevalence and mortality. Developing nations, in particular, showed notably large increases (Figure 3 and Supplementary Tables 4–7). We have selected China, the Russian Federation, Germany and the United States as representative cases for illustration, mainly because they represent different levels of disease burden, specific patterns of trends, or different stages of economic development, etc. The number of MASLD cases in China grew from 72,749,854 (95% UI: 64,057,475–82,692,295) in 1990 to 84,743,324 (95% UI: 75,074,753–96,388,231) in 2021. In terms of mortality, the Russian Federation displayed a marked escalation, with MASLD deaths increasing from 158.01 (95% UI: 96.67–246.21) in 1990 to 1,593.98 (95% UI: 944.34–2488.09) in 2021, yielding an annual growth rate of 7.32 (95% CI: 6–8.65). Conversely, Germany experienced a decline, with MASLD deaths decreasing from 402.55 (95% UI: 229.45–631.98) to 212.41 (95% UI: 122.29–319.64), at an annual reduction rate of −2.03 (95% CI: −2.48 to −1.57). Meanwhile, the United States saw MASLD cases increase from 11,550,618 (95% UI: 10,219,699–13,107,974) in 1990 to 17,225,817 (95% UI: 15,306,637–19,488,473) in 2021, corresponding to an annual growth rate of 0.96 (95% CI: 0.89–1.03). Substantial variation exists among countries in both MASLD burden and trends. Developing countries—particularly China and India—confront more critical MASLD epidemics, whereas some developed nations, such as Germany, have achieved relative control over MASLD-related mortality.

### 3.5 Sex differences

**FIGURE 4:**
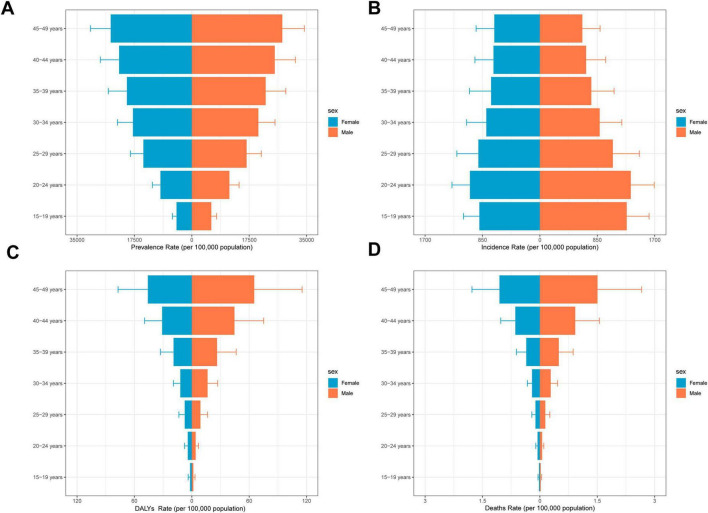
Overall time trends by sex and age. **(A)** Prevalence rates by age group among the adolescents and adults aged 15–49 years by sex. **(B)** Incidence rates by age group among the adolescents and adults aged 15–49 years by sex. **(C)** DALYs rates by age group among the adolescents and adults aged 15–49 years by sex. **(D)** Death rates by age group among the adolescents and adults aged 15–49 years by sex.

**FIGURE 5:**
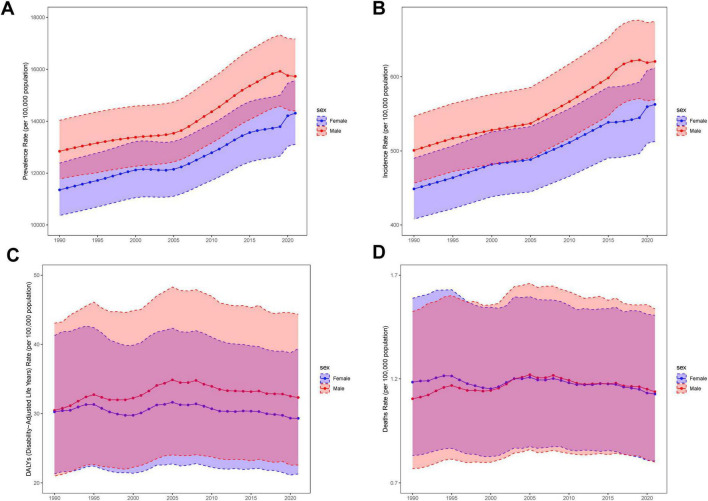
Structural analysis of sex and age. The solid line indicates the mean and the dashed line is the 95% confidence interval. **(A)** Trends in prevalence by sex, from1990 to 2021. **(B)** Trends in incidence by sex, from1990 to 2021. **(C)** Trends in DALYs by sex, from1990 to 2021. **(D)** Trends in deaths by sex, from1990 to 2021.

Although the overall burden of MASLD increased worldwide, diverging growth patterns by sex reveal distinct risk profiles (Figures 4, 5 and Table 2). Specifically, total global MASLD cases rose from 342.94 million in 1990 to 665.77 million in 2021, with female cases climbing from 1.557 million to 3.0711 million and male cases from 187.24 million to 358.66 million. In incidence, females showed a growth rate of 0.93 (95% CI: 0.87–0.99), whereas males reached 0.97 (95% CI: 0.89–1.05). Although the rise in prevalence was slightly higher among females, males exhibited faster growth in MASLD-related mortality and disease burden, with deaths increasing by 100% and DALYs by 97%. Female deaths rose from 3492.95 to 6272.18, while male deaths increased from 4426.82 to 8835.70. With respect to DALYs, females increased from 177.08 thousand to 313.98 thousand person-years, while males rose from 221.77 thousand to 436.65 thousand person-years.

**TABLE 2:** Global prevalence, incidence, DALYs, and death rates and trends of MASLD by sex among the adolescents and adults aged 15–49 years from 1990 to 2021.

	Cases			Rates		
Sex	1990 (95% UI)	2021 (95% UI)	Percentage change (%)	1990 per 100,000 (95% UI)	2021 per 100,000 (95% UI)	EAPC (95% CI)
Female (prevalence)	155.7 million (138.19–176.88)	307.11 million (273.95–347.8)	0.97 (0.97–0.98)	11642.18 (10333.13–13226.01)	15758.57 (14056.98–17846.21)	0.93
Male (prevalence)	187.24 million (166.77–211.92)	358.66 million (322.29–406.48)	0.92 (0.92–0.93)	13636.43 (12145.25–15433.83)	17935.27 (16116.55–20326.88)	0.97
Female (incidence)	8.9 thousand (7.8–10.05)	16.1 thousand (14.23–18.2)	0.81 (0.81–0.82)	665.21 (583.13–751.2)	825.88 (730.12–934.09)	0.68
Male (incidence)	10.92 thousand (9.57–12.32)	19.51 thousand (17.34–21.92)	0.79 (0.78–0.81)	795.2 (696.76–897.11)	975.4 (866.93–1096.18)	0.76
Female (DALYs)	177.08 Thousand (116.23–266.15)	313.98 thousand (203.2–470.89)	0.77 (0.75–0.77)	13.24 (8.69–19.9)	16.11 (10.43–24.16)	0.61
Male (DALYs)	221.77 thousand (144.41–338.25)	436.65 thousand (274.55–667.76)	0.97 (0.90–0.97)	16.15 (10.52–24.63)	21.84 (13.73–33.39)	0.88
Female (death)	3492.95 (2244.09–5322.72)	6272.18 (4001.04–9395.68)	0.8 (0.77–0.8)	0.26 (0.17–0.4)	0.32 (0.21–0.48)	0.65
Male (death)	4426.82 (2779.56–6842.98)	8835.7 (5462.83–13698.51)	1 (0.97–1)	0.32 (0.2–0.5)	0.44 (0.27–0.69)	0.92

### 3.6 Age patterns

**FIGURE 6:**
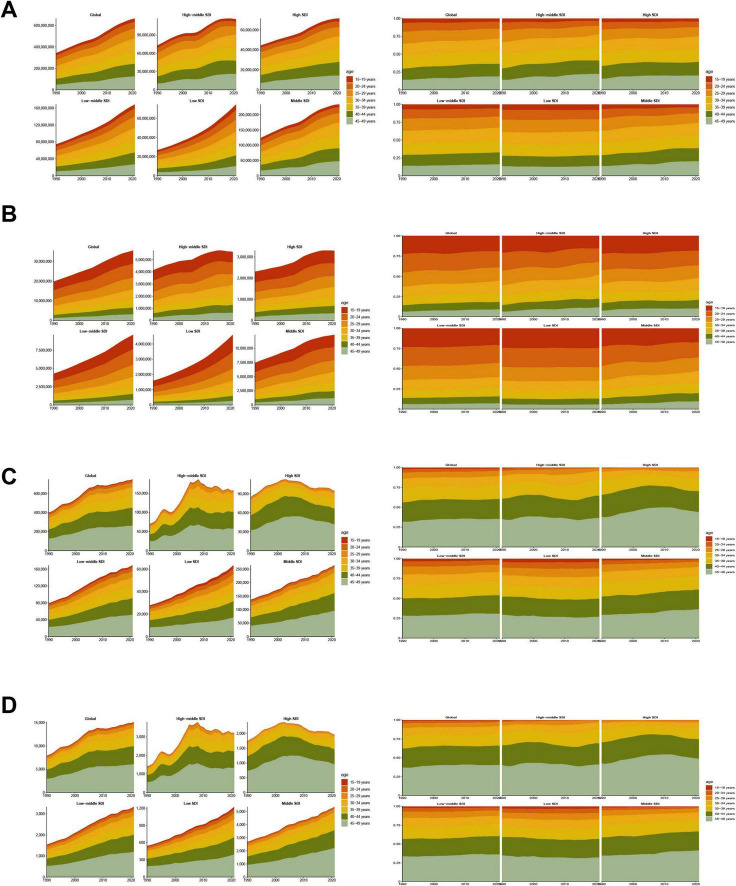
Temporal trend of MASLD burden among the adolescents and adults aged 15–49 years by age pattern in different regions. **(A)** Prevalent case and percentage change of seven age groups from 1990 to 2021 globally and in five regions low to high SDI. **(B)** Incident case and percentage change of seven age groups from 1990 to 2021 globally and in five regions (low to high SDI). **(C)** DALYs and percentage change of seven age groups from 1990 to 2021 globally and in five regions (low to high SDI). **(D)** Deaths and percentage change of seven age groups from 1990 to 2021 globally and in five regions (low to high SDI).

Over the last 30 years, adolescents and adults worldwide experienced significant increases in MASLD prevalence, mortality, incidence, and DALYs across all age groups, albeit with notable heterogeneity by age and SDI region (Figure 6, Table 3, Supplementary Tables 8–10). Specifically, in 2021, the global prevalence rate for the 15–19 age group was approximately 5310.34 per 100,000 population. In contrast, the burden increased sharply in the 45–49 age group, with a prevalence rate reaching 26166.61 per 100,000, an incidence rate of 67.35 per 100,000, a DALY rate of 55.64 per 100,000, and a mortality rate climbing to 1.28 per 100,000. Regarding trends from 1990 to 2021, prevalence and DALY rates showed increasing trends across almost all age groups. The EAPC for prevalence was generally positive across age groups, ranging roughly from 0.59 (15–19 years) to 0.79 (35–49 years), indicating a continuous growth in the prevalence burden. The trend for incidence rates was comparatively stable, even showing slight decreases in most age groups (EAPC range from −0.27 to −0.12), with only a slight overall increase observed for the 15–49 age group as a whole (EAPC = 0.20).

**TABLE 3:** The prevalent cases and prevalence rates for MASLD among the adolescents and adults aged 15–49 years in age patterns from 1990 to 2021.

		Prevalent cases			Prevalence rates		
Location	Age (year)	1990 million (95%UI)	2021 million (95%UI)	Percentage change (%)	1990 per 100,000 (95% UI)	2021 per 100,000 (95% UI)	EAPC (95% CI)
Global	15–49 years	342.94 (305.23–388.8)	665.77 (596.51–754.28)	0.94 (0.95–0.94)	12652.46 (11261.11–14344.35)	16860.94 (15106.92–19102.52)	0.95 (0.88–1.02)
Global	15–19 years	23.02 (17.61–29.27)	33.14 (25.33–42.28)	0.44 (0.44–0.44)	4431.58 (3390.87–5635.32)	5310.34 (4059.95–6775.27)	0.59 (0.58–0.61)
Global	20–24 years	42.62 (32.06–53.67)	62.84 (47.58–78.98)	0.47 (0.48–0.47)	8660.25 (6514.4–10907.27)	10523.81 (7967.3–13226.73)	0.66 (0.63–0.69)
Global	25–29 years	57.08 (44.47–72.76)	92.67 (72.32–116.89)	0.62 (0.63–0.61)	12896.13 (10046.56–16438.03)	15750.91 (12292.86–19867.8)	0.72 (0.68–0.75)
Global	30–34 years	59.48 (45.44–75)	115.71 (88.74–145.46)	0.95 (0.95–0.94)	15433.52 (11789.84–19458.17)	19141.79 (14681.2–24063.13)	0.77 (0.71–0.83)
Global	35–39 years	59.13 (45.61–76.11)	118.7 (91.71–152.13)	1.01 (1.01–1)	16785.52 (12948.98–21606.99)	21163.61 (16352.33–27124.36)	0.79 (0.71–0.87)
Global	40–44 years	53.53 (41.65–67.88)	118.81 (92.61–148.51)	1.22 (1.22–1.19)	18683.62 (14538.64–23695.4)	23750.45 (18513.06–29686.47)	0.79 (0.71–0.88)
Global	45–49 years	48.09 (37.91–60.14)	123.9 (97.87–153.47)	1.58 (1.58–1.55)	20711.32 (16325.21–25902.3)	26166.61 (20669.55–32411.55)	0.79 (0.71–0.87)
Low SDI	15–49 years	26.9 (23.83–30.3)	74.77 (66.25–84.79)	1.78 (1.78–1.8)	12169.65 (10781.77–13707.42)	13784.9 (12214.02–15633.8)	0.41 (0.37–0.46)
Low SDI	15–19 years	2.1 (1.59–2.68)	5.79 (4.39–7.4)	1.76 (1.76–1.76)	4148.37 (3143.24–5284.71)	4672.21 (3539.46–5965.26)	0.39 (0.35–0.42)
Low SDI	20–24 years	3.67 (2.77–4.58)	10.16 (7.67–12.72)	1.77 (1.77–1.78)	8615.64 (6498.54–10744.38)	9746.06 (7356.45–12202.61)	0.38 (0.34–0.42)
Low SDI	25–29 years	4.63 (3.59–5.88)	12.67 (9.9–16.19)	1.74 (1.76–1.75)	12917.05 (10034.21–16416.18)	14711.37 (11490.33–18793.57)	0.39 (0.36–0.42)
Low SDI	30–34 years	4.62 (3.56–5.85)	12.75 (9.78–16.27)	1.76 (1.75–1.78)	15561.48 (11970.39–19697.1)	17609.83 (13505.88–22474.96)	0.39 (0.36–0.41)
Low SDI	35–39 years	4.38 (3.38–5.69)	12.03 (9.24–15.59)	1.75 (1.73–1.74)	17177.1 (13235.54–22310.47)	19315.74 (14836.58–25035.73)	0.39 (0.36–0.43)
Low SDI	40–44 years	3.89 (3.02–4.92)	11.29 (8.77–14.26)	1.9 (1.9–1.9)	19533.04 (15132.05–24704)	21874.32 (16982.65–27629.45)	0.4 (0.35–0.46)
Low SDI	45–49 years	3.6 (2.81–4.56)	10.07 (7.85–12.62)	1.8 (1.79–1.77)	21445.23 (16698.19–27128.36)	24152.05 (18818.95–30262.77)	0.37 (0.31–0.44)
Low–middle SDI	15–49 years	74.4 (65.93–84.03)	168.96 (151.25–190.38)	1.27 (1.29–1.27)	13499.83 (11964.24–15247.71)	16625.4 (14883.45–18733.27)	0.67 (0.62–0.73)
Low–middle SDI	15–19 years	5.41 (4.14–6.89)	9.85 (7.52–12.55)	0.82 (0.82–0.82)	4551.04 (3482.14–5793.41)	5339.84 (4074.74–6801.96)	0.52 (0.49–0.54)
Low–middle SDI	20–24 years	9.75 (7.38–12.18)	19.12 (14.51–24.03)	0.96 (0.97–0.97)	9349.18 (7077.17–11677.96)	10937.11 (8298.46–13746.64)	0.52 (0.49–0.55)
Low–middle SDI	25–29 years	12.61 (9.79–16.1)	26.49 (20.63–33.52)	1.1 (1.11–1.08)	14063.72 (10927.6–17959.97)	16362.26 (12744.08–20708.02)	0.51 (0.47–0.55)
Low–middle SDI	30–34 years	12.82 (9.81–16.29)	29.18 (22.39–36.69)	1.28 (1.28–1.25)	16973.61 (12985.76–21580.15)	19740.98 (15148.81–24820.55)	0.51 (0.45–0.56)
Low–middle SDI	35–39 years	12.15 (9.37–15.65)	29.06 (22.45–37.34)	1.39 (1.4–1.39)	18677.4 (14402.11–24055.86)	21779.3 (16826.95–27979.59)	0.49 (0.43–0.56)
Low–middle SDI	40–44 years	11.23 (8.71–14.16)	28.46 (22.11–35.73)	1.53 (1.54–1.52)	21157.97 (16406.1–26686.7)	24688.68 (19181.05–31000.96)	0.48 (0.43–0.54)
Low–middle SDI	45–49 years	10.43 (8.16–13.06)	26.79 (21.05–33.37)	1.57 (1.58–1.56)	23376.72 (18277.53–29270.41)	27204.13 (21372.16–33880.28)	0.49 (0.45–0.53)
Middle SDI	15–49 years	123.77 (109.84–140.46)	233.97 (209.77–265.71)	0.89 (0.91–0.89)	13592.57 (12061.92–15425.05)	18642.26 (16713.97–21170.64)	1.04 (0.97–1.12)
Middle SDI	15–19 years	8.62 (6.62–10.89)	10.17 (7.76–12.99)	0.18 (0.17–0.19)	4600.78 (3535.14–5814.87)	5578.29 (4256.42–7122.2)	0.62 (0.61–0.64)
Middle SDI	20–24 years	16.28 (12.12–20.68)	19.6 (14.7–24.76)	0.2 (0.21–0.2)	9128 (6797.92–11599.74)	11055.78 (8294.17–13968.47)	0.64 (0.62–0.67)
Middle SDI	25–29 years	21.04 (16.41–26.76)	30.64 (23.81–38.59)	0.46 (0.45–0.44)	13936.41 (10872.77–17723.75)	16667.5 (12955.12–20994.85)	0.7 (0.66–0.74)
Middle SDI	30–34 years	20.99 (16.06–26.4)	40.92 (31.28–51.55)	0.95 (0.95–0.95)	17126.78 (13107.41–21542.67)	20512.92 (15679.71–25840.4)	0.69 (0.61–0.78)
Middle SDI	35–39 years	21.28 (16.39–27.48)	42.58 (32.89–54.35)	1 (1.01–0.98)	18753.84 (14442.46–24217.13)	23063.46 (17816–29437.22)	0.7 (0.58–0.81)
Middle SDI	40–44 years	18.75 (14.63–23.88)	43.13 (33.78–53.91)	1.3 (1.31–1.26)	21412.27 (16705.38–27270.66)	26152.4 (20483.29–32687.48)	0.65 (0.54–0.75)
Middle SDI	45–49 years	16.82 (13.29–20.96)	46.94 (36.87–57.93)	1.79 (1.77–1.76)	23893.84 (18886.18–29783.29)	28857.49 (22668.52–35613.26)	0.62 (0.54–0.7)
High–middle SDI	15–49 years	73.05 (64.99–82.95)	116.04 (103.31–132.08)	0.59 (0.59–0.59)	12942.38 (11514.62–14697.06)	18432.23 (16409–20979.04)	1.16 (1.02–1.3)
High–middle SDI	15–19 years	4.33 (3.3–5.48)	4.18 (3.19–5.31)	–0.03 (–0.03—-0.03)	4487.21 (3421.1–5677.99)	5766.58 (4405.76–7326.89)	0.88 (0.81–0.96)
High–middle SDI	20–24 years	8.33 (6.24–10.56)	8.18 (6.1–10.34)	–0.02 (–0.02—-0.02)	8532.69 (6398.09–10823.13)	10910.28 (8132.52–13790.82)	0.81 (0.68–0.94)
High–middle SDI	25–29 years	11.84 (9.18–15.07)	13.69 (10.55–17.26)	0.16 (0.15–0.15)	12745.37 (9876.04–16212.95)	16162.2 (12463.05–20382.05)	0.82 (0.72–0.92)
High–middle SDI	30–34 years	13.08 (10–16.52)	20.67 (15.9–25.85)	0.58 (0.59–0.56)	15341.64 (11726.01–19375.38)	19370.33 (14900.47–24223.29)	0.81 (0.69–0.93)
High–middle SDI	35–39 years	13.48 (10.34–17.39)	21.55 (16.63–27.59)	0.6 (0.61–0.59)	16797.12 (12892.51–21680.28)	21247.38 (16392.59–27197.85)	0.82 (0.68–0.96)
High–middle SDI	40–44 years	11.72 (9.08–14.82)	22.02 (17.16–27.53)	0.88 (0.89–0.86)	18766.06 (14531.76–23728.95)	23835.72 (18569.48–29792.88)	0.8 (0.66–0.93)
High–middle SDI	45–49 years	10.27 (8.11–12.95)	25.75 (20.24–32.26)	1.51 (1.5–1.49)	20781.04 (16423.81–26201.69)	26575.61 (20884.94–33288.93)	0.81 (0.68–0.95)
High SDI	15–49 years	44.5 (39.62–50.35)	71.5 (63.92–81.01)	0.61 (0.61–0.61)	9656.52 (8597.73–10925.33)	14237.03 (12726.7–16129.17)	1.35 (1.31–1.39)
High SDI	15–19 years	2.53 (1.94–3.23)	3.11 (2.4–3.93)	0.23 (0.24–0.22)	3867.83 (2964.3–4924.7)	5171.64 (3984.23–6537.96)	0.97 (0.95–0.99)
High SDI	20–24 years	4.56 (3.43–5.74)	5.74 (4.33–7.22)	0.26 (0.26–0.26)	6615.72 (4986.07–8327.24)	8775.4 (6613.58–11038.73)	1.05 (0.99–1.11)
High SDI	25–29 years	6.92 (5.38–8.84)	9.12 (7.07–11.56)	0.32 (0.31–0.31)	9487.89 (7386.31–12127.35)	12771.72 (9903.01–16195.54)	1.21 (1.11–1.3)
High SDI	30–34 years	7.92 (6–10.09)	12.1 (9.35–15.13)	0.53 (0.56–0.5)	10994.1 (8328.41–14004.75)	15591.02 (12052.13–19500.39)	1.38 (1.31–1.46)
High SDI	35–39 years	7.78 (5.98–10.06)	13.38 (10.35–17.08)	0.72 (0.73–0.7)	11502.71 (8833.54–14876.38)	17014.35 (13155.2–21717.33)	1.39 (1.32–1.46)
High SDI	40–44 years	7.87 (6.09–9.99)	13.81 (10.77–17.32)	0.75 (0.77–0.73)	12469.68 (9638.36–15826.86)	18258.83 (14238.4–22900.99)	1.28 (1.22–1.33)
High SDI	45–49 years	6.92 (5.39–8.77)	14.24 (11.28–17.71)	1.06 (1.09–1.02)	13639.59 (10615.78–17291.63)	19412.89 (15370.08–24136.55)	1.19 (1.15–1.24)

The DALY rate climbed steeply from 1.73 per 100,000 in the 15–19 age group to 55.64 per 100,000 in the 45–49 age group, showing that health loss due to the disease magnifies substantially with advancing age. Observing the trends, the increase in DALY rates was particularly pronounced in the 20–39 years age brackets, with EAPCs between 0.43 and 0.77. However, in the 40–44 (EAPC = 0.15) and 45–49 (EAPC = −0.07) age groups, the growth trend for DALY rates decelerated or even slightly decreased, which could be related to multiple factors, including enhanced interventions or competing risks from other causes of mortality. The age pattern for mortality rates was even steeper, increasing significantly from a very low level of 0.02 per 100,000 in the 15–19 age group to 1.28 per 100,000 in the 45–49 age group. Similar to DALYs, the growth trend in mortality was most significant in the 20–39 age groups (EAPC range 0.42–0.75), whilst decelerating in those over 40 years old (EAPC = 0.15 for 40–44 years; EAPC = −0.07 for 45–49 years). This suggests that although the absolute mortality burden is highest in the older age groups, its rate of increase has slowed compared to younger and middle-aged adults. Overall, the health damage and risk of premature mortality caused by MASLD accumulate significantly with age, with the burden accelerating particularly after the age of 30, although the rate of increase in DALYs and mortality for the oldest age group (45–49 years) slowed during the latter part of the study period.

All SDI regions exhibited the fundamental pattern of increasing prevalence, incidence, DALY, and mortality rates with age. However, the absolute levels of burden and the growth trends differed markedly between regions. In High-SDI regions, although prevalence rates were generally higher across all age groups compared to other regions (e.g., 19412.89 per 100,000 in the 45–49 age group in 2021), the EAPC were relatively stable or slightly decreasing, particularly for incidence rates (EAPCs were negative across all age groups, range −0.98 to −0.42), DALYs (EAPCs were negative for age groups 30 years and above, range −0.95 to −0.35), and mortality (EAPCs were negative for age groups 30 years and above, range −0.95 to −0.34). This indicates that the burden is stabilizing or even declining at these high levels. In contrast, the burden growth trends were more pronounced in High-middle-SDI and Middle-SDI regions, especially among individuals over 30 years old. For instance, DALY rates (EAPCs between 1.28 and 2.94) and mortality rates (EAPCs between 0.30 and 2.95) in High-middle-SDI regions showed strong upward trends in the 30–49 age group. Similar increases were observed in Middle-SDI regions, with DALY rates (EAPC range 0.19–1.02) and mortality rates (EAPC range 0.19–1.12) trending upwards in most age groups. In Low-middle-SDI regions, although the absolute burden level was relatively lower, DALY and mortality rates also showed increases for the 15–49 age group overall (EAPC 0.56 and 0.61, respectively) and within several age sub-groups. Low-SDI regions displayed a somewhat unique pattern. Despite having the lowest absolute burden, the EAPCs for incidence, DALY, and mortality rates between 1990 and 2021 were negative or close to zero for most age groups, suggesting that burden growth may have stabilized or decreased. However, this could also be influenced by factors such as data quality and disease surveillance capabilities. These differences indicate heterogeneity in the age-related burden patterns and evolutionary trends of MASLD across regions of varying development levels.

### 3.7 The association between adolescents and adults MASLD burden and SDI

**FIGURE 7:**
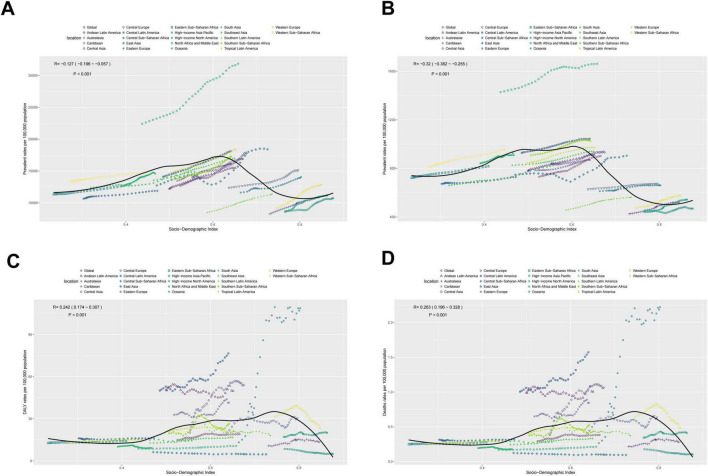
The associations between the SDI and MASLD burden among the adolescents and adults aged 15–49 years across 21 GBD regions. **(A)** Prevalent rates per 100,000 population. **(B)** Incident rates per 100,000 population. **(C)** DALYs rates per 100,000 population. **(D)** Deaths rates per 100,000 population.

As illustrated in Figure 7, MASLD prevalence in adolescents and adults is negatively associated with SDI overall [R = −0.127, 95% CI: (−0.196, −0.057); *P* < 0.001]. Specifically, in regions where SDI is below 0.6, prevalence tends to increase slightly with SDI; however, the magnitude of change is limited. Once SDI increases from 0.6 to 0.8, prevalence exhibits a marked decline, remaining low at SDI > 0.8. Likewise, incidence is significantly negatively correlated with SDI [R = −0.32, 95% CI: (−0.382, −0.255); *P* < 0.001]: it remains relatively stable below SDI 0.6, decreases substantially between 0.6 and 0.8, and stabilizes again above 0.8.

By contrast, mortality displays a significant positive correlation with SDI [R = 0.263, 95% CI: (0.196, 0.328); *P* < 0.001]. In regions with SDI below 0.7, mortality grows slowly and remains fairly stable; however, as SDI surpasses 0.7, mortality declines appreciably. DALYs also show a positive correlation with SDI [R = 0.242, 95% CI: (0.174, 0.307); *P* < 0.001]. Similar to mortality, once SDI reaches around 0.7–0.8, DALYs drop, remaining significantly lower than those in low-SDI regions.

## 4 Discussion

This study systematically assessed the global spatial and temporal burden of MASLD from 1990 to 2021 based on the GBD 2021 data, and stratified the analysis by SDI, age, and sex to fill the gap of the lack of overall epidemiologic evidence in the working-age population aged 15–49 years.

Notably, emerging evidence suggests that MASLD is increasingly being detected in populations without traditionally recognized risk factors, such as individuals with normal body weight—referred to as “lean MASLD”—indicating that the disease burden may extend beyond classical high-risk groups (e.g., those with obesity or metabolic syndrome) (23, 24). Both incidence and DALYs have exhibited a considerably high rate of increase, with growth particularly pronounced in certain low-SDI and low-middle-SDI countries and regions. Key drivers of this trend can be traced to rapid global urbanization, the westernization of diets, and deeper transformations in lifestyle patterns over recent years: the imbalance between excessive caloric intake and insufficient physical activity resulted in progressively severe metabolic derangements. Under such conditions, individuals readily progress from simple steatosis to steatohepatitis and, in some cases, ultimately to cirrhosis or hepatocellular carcinoma (25). A recent state-of-the-art review synthesizes these progression pathways across diverse phenotypes of MASLD (26). Moreover, insufficient early prevention and screening result in many mild-to-moderate MASLD cases failing to receive timely clinical intervention, with diagnoses often made only at relatively advanced stages, thereby exacerbating the overall disease burden (27).

Although the total number of MASLD cases is also rising in high-SDI regions, these areas may benefit from more robust clinical and preventive infrastructures, coupled with heightened health awareness and strengthened public health support systems. Consequently, the management of moderate-to-severe MASLD and the containment of mortality appears more effective, contributing to slowed or reduced mortality and DALYs in some high-SDI countries (27–29).

In high-SDI regions, the phenomenon of stable or declining MASLD incidence rates alongside persistently high or rising prevalence can be primarily explained by the epidemiological principle where prevalence (P) approximates incidence (I) multiplied by average disease duration (D) (P ≈ I × D). Thus, even with slower growth in new cases, a significant prolongation of average disease duration due to several factors can lead to a continued accumulation of prevalent cases: first, medical advancements may have reduced MASLD-specific mortality, thereby extending patient survival with the disease; second, the limited long-term effectiveness of lifestyle interventions and the lack of highly effective pharmacological treatments may contribute to low natural remission rates (18); and third, a reduction in premature mortality from other acute or competing diseases allows more individuals to survive longer and manifest chronic conditions like MASLD (30). Therefore, MASLD control strategies in high-SDI regions must not only focus on reducing incidence risk but also emphasize the long-term management and outcome improvement of the large pool of existing patients to effectively control the overall disease burden.

By contrast, low-SDI and low-middle-SDI regions exhibit rapid increases in mortality and health loss, particularly in Eastern Europe and Central Asia, where the surge in deaths and DALYs related to MASLD underscores the inadequate disease control and intervention in these populations. Socioeconomic and cultural backgrounds in these areas are highly complex: on one hand, rapid lifestyle westernization and economic transitions expose more individuals to risks associated with unhealthy dietary patterns—which include not only excessive caloric intake but also potentially detrimental changes in specific nutrient intakes (31). Indeed, research has highlighted the impact of particular dietary components, such as sodium intake, on outcomes for MASLD patients (32); on the other, constrained medical resources, limited public health funding, and the absence of systematic strategies for early MASLD detection and management jointly accelerates disease progression toward more severe stages. Moreover, longstanding dietary deficiencies, economic unrest, or regional conflict in some areas further impedes the availability of timely interventions and treatment (33). In terms of diagnostics, numerous countries or regions have yet to establish comprehensive screening, follow-up, and oversight systems for chronic liver diseases, and lacking precise mechanisms to identify high-risk groups (e.g., obesity, type 2 diabetes, metabolic syndrome) (34). At the same time, attention in these regions tends to focus on other chronic liver diseases, such as hepatitis B and C, often leading to neglect of MASLD. Once disease progression accelerates, apart from causing a severe decline in individual quality of life, substantial healthcare costs and caregiving burdens are imposed on society and families.

Regarding sex- and age- specific differences, males and females face distinct pathophysiological challenges in the onset and development of MASLD. Compared with females, males generally exhibit a higher MASLD prevalence. A systematic review and meta-analysis indicated that the risk of MASLD in females is 19% lower than males in the general population (35), while another study reported global prevalence rates of 36.6% in males and 25.5% in females (4). Overall, females may benefit from partial estrogenic protection prior to menopause, resulting in a comparatively slower MASLD progression. Yet, drastic lifestyle changes (e.g., high-sugar diets, nocturnal snacking, and sleep deprivation) and the growing impact of obesity and insulin resistance on young women are causing their prevalence rates to climb quickly (36–39). In addition, pregnancy and other reproductive factors may compound effects on maternal and infant health—an area of risk still insufficiently quantified (15, 40). Once MASLD is established, however, females carry a higher risk of progression to more severe forms of liver disease (35).

Metabolic dysfunction-associated steatotic liver disease in the 15–24 years adolescent and young adult group is an emerging concern where rising prevalence parallels increasing obesity and metabolic syndrome in this age bracket. Reports indicate that MASLD is highly prevalent among younger individuals; one study suggests that approximately 20.7% of those around 24 years old exhibit suspected steatosis, and 2.7% already have fibrosis (41). In many cases, the pathogenesis and clinical course of MASLD in this group are overlooked by traditional perspectives, which regards adolescence as a “window of opportunity for health” with relatively well-maintained metabolic function and fewer chronic diseases. However, with the widespread westernization of dietary habits, declining levels of physical activity, and the sedentary lifestyle facilitated by electronic devices, young individuals are increasingly prone to energy imbalance (42–44). Studies have shown that the metabolic damage caused by excessive sugar and high-fat intake can accumulate from adolescence onward; some do not yet meet obesity thresholds but already exhibit considerable visceral fat accumulation and insidious metabolic inflammation (45–47). These young patients often display no obvious clinical symptoms and, along with insufficient health education and early screening, remain in a “subclinical” state during the early stages of disease. Consequently, by the time they reach the 25–34 or 35–44 age brackets, the interplay of work and life stress can spur MASLD to escalate rapidly into MASH, with a heightened risk of hepatic fibrosis during acute stress (48). A cause for concern is that upon entering the 35–44 age group, individuals who have accumulated MASLD since their teenage years may be at a higher risk of cirrhosis or severe hepatic impairment than those who develop metabolic disorders only in middle or older age. In other words, neglecting the 15–24 years cohort ultimately burdens the middle-aged population with a greater health toll, compromising personal quality of life and imposing substantial social and economic consequences. By the time individuals reach 45–49 years of age, this group experiences the highest incidence and prevalence of MASLD and, correspondingly, marked increases in DALYs, as observed in this study. Given that individuals aged 15–49 represent the core of the working-age population, the increasing MASLD burden in this group may also compromise work capacity, reduce enterprise productivity, and exert broader socioeconomic effects. These trends highlight the need to incorporate MASLD prevention into occupational health promotion and workplace wellness programs. As this younger population ages, the cumulative risk of developing MASLD-related comorbidities is expected to rise markedly, potentially intensifying the long-term burden of non-communicable diseases. These findings underscore the critical importance of implementing early prevention and lifestyle interventions among adolescents and young adults to reduce future multimorbidity and healthcare strain.

Although this study provides valuable insights into the global epidemiological trends of MASLD in the 15–49 age group, several limitations should be acknowledged. First, the data are derived from the GBD database, which, despite its extensive coverage and methodological rigor, may be subject to time lags. Moreover, in low- and middle-income countries, limitations in disease surveillance systems could result in underreporting or inaccurate estimations. Second, although the GBD study has made substantial efforts to standardize data collection and processing, the actual variability in MASLD diagnostic criteria and disease definitions across regions and time periods may still affect the precision and comparability of prevalence estimates. These discrepancies are particularly evident in resource-limited settings, where heterogeneous diagnostic methodologies can lead to underdiagnosis or misclassification. Current diagnostic approaches for MASLD face considerable challenges, especially for large-scale screening. In this context, emerging technologies such as artificial intelligence in medical imaging present a promising future direction to enhance early detection, risk stratification, and monitoring of MASLD, potentially alleviating some of these diagnostic burdens (49). Third, although this study stratifies MASLD burden by SDI level, real-world differences in cultural practices, dietary behaviors, and national health policies remain complex and may influence the actual disease burden in ways not fully captured by SDI alone. Lastly, given the rapid evolution of global lifestyles—particularly in the aftermath of the COVID-19 pandemic—current estimates may not fully reflect the most recent shifts in MASLD prevalence and trends. Continued efforts to establish robust, up-to-date, and regionally representative data are essential for enhancing the accuracy of burden assessments, forecasting future trends, and informing effective public health strategies targeting MASLD in the working-age population.

## Data Availability

The original contributions presented in this study are included in this article/Supplementary material, further inquiries can be directed to the corresponding author.

## Footnotes

1
https://ghdx.healthdata.org/gbd-2021/sources
