# Supplementary material for: Global, regional and national burden of Metabolic dysfunction-associated steatotic liver disease in adolescents and adults aged 15–49 years from 1990 to 2021: results from the 2021 Global Burden of Disease study
Source: Front Med (Lausanne). 2025 Jun 25;12:1568211. doi: 10.3389/fmed.2025.1568211 (PMC12237898; doi:10.3389/fmed.2025.1568211)
Supplement: Supplementary file 1 [file Supplementary_file_1.ZIP › Supplementary Table 4 .docx]

**Supplementary Table 4** The prevalence of MASLD cases and rates among the adolescents and adults aged 15-49 years in 1990 and 2021 across 204 countries, and the trends from 1990 to 2021

| **location** | **Prevalence cases** | | | **Prevalence rates** | | |
| --- | --- | --- | --- | --- | --- | --- |
|  | **1990**  **(95%UI)** | **2021**  **(95%UI)** | **percentage**  **Change**  **(100%)** | **1990**  **Per 100,000**  **(95%UI)** | **2021**  **per 100,000 (95%UI)** | **EAPC**  **(95% CI)** |
| Afghanistan | 727498.54 (640658.1-822363.75) | 3286384.76 (2915039.38-3720430.85) | 3.52 | 18190.81 (16019.4-20562.87) | 22236.66 (19724.03-25173.55) | 0.87 (0.81-0.93) |
| Albania | 253284.41 (221874.25-291745.49) | 224860.59 (199016.88-256458.36) | -0.11 | 14800.5 (12965.07-17047.94) | 17855.39 (15803.23-20364.45) | 0.46 (0.42-0.51) |
| Algeria | 2445143.33 (2159647.4-2781622.89) | 7121431.01 (6350526.55-8053734.95) | 1.91 | 20772.44 (18347.05-23630.97) | 31447.51 (28043.27-35564.47) | 1.46 (1.4-1.51) |
| American Samoa | 4083.62 (3605.72-4623.7) | 5151.75 (4567.09-5803.07) | 0.26 | 16772.36 (14809.5-18990.58) | 21484.12 (19045.95-24200.31) | 0.78 (0.67-0.88) |
| Andorra | 3051.72 (2683.95-3509.51) | 5289.81 (4615.86-6093.72) | 0.73 | 9414.63 (8280.05-10826.95) | 12826.5 (11192.34-14775.78) | 1.03 (0.99-1.08) |
| Angola | 504662.57 (443588.26-574162.5) | 1650869.91 (1443386.89-1881125.33) | 2.27 | 10842.47 (9530.31-12335.64) | 11259.02 (9843.97-12829.37) | 0.16 (0.14-0.19) |
| Antigua and Barbuda | 4668.8 (4146.34-5282.21) | 8095.65 (7122.43-9162.42) | 0.73 | 14856.46 (13193.94-16808.35) | 17084.87 (15031.02-19336.16) | 0.44 (0.38-0.49) |
| Argentina | 1318226.37 (1156598.14-1508729.05) | 2586419.38 (2272276.92-2938667.58) | 0.96 | 8293.89 (7276.97-9492.47) | 10987.17 (9652.68-12483.52) | 0.94 (0.93-0.95) |
| Armenia | 261541.94 (228724.13-301320.02) | 286374.93 (251316.22-328797.87) | 0.09 | 15320.04 (13397.7-17650.07) | 19666.84 (17259.18-22580.24) | 0.76 (0.72-0.8) |
| Australia | 739420.93 (650821.02-839295.19) | 1306516.18 (1153272.12-1488706.13) | 0.77 | 8228.08 (7242.16-9339.45) | 10880.82 (9604.58-12398.12) | 0.89 (0.84-0.94) |
| Austria | 361437.22 (319509.43-409918.56) | 502363.97 (443124.99-573604.51) | 0.39 | 8978.27 (7936.76-10182.56) | 12452.16 (10983.8-14218.01) | 1.09 (1.04-1.14) |
| Azerbaijan | 558003.52 (489730.41-636465.76) | 1134657.01 (1006423.22-1301503.07) | 1.03 | 15202.37 (13342.32-17340.01) | 20366.37 (18064.66-23361.15) | 1 (0.94-1.05) |
| Bahamas | 18186.74 (15891.75-20766.25) | 34900.36 (30899.52-39706.08) | 0.92 | 12775.04 (11162.96-14586.98) | 16644.92 (14736.81-18936.9) | 0.88 (0.79-0.96) |
| Bahrain | 80007.01 (70631.04-90816.31) | 357582.29 (319071.09-401315.3) | 3.47 | 26520.16 (23412.28-30103.16) | 36515.57 (32582.88-40981.49) | 1.13 (1.08-1.17) |
| Bangladesh | 6240397.55 (5503150.18-7074952.23) | 13802358.1 (12228209.2-15574091.8) | 1.21 | 12486.25 (11011.11-14156.08) | 15691.16 (13901.59-17705.35) | 0.78 (0.76-0.81) |
| Barbados | 19533.55 (17302.21-22460.45) | 24103.49 (21105.04-27468.03) | 0.23 | 14504.58 (12847.71-16677.95) | 17250.16 (15104.25-19658.06) | 0.54 (0.45-0.62) |
| Belarus | 543754.2 (479883.79-619187.28) | 550618.13 (484748.85-626724.44) | 0.01 | 10783.89 (9517.2-12279.9) | 12941.39 (11393.24-14730.14) | 0.53 (0.48-0.58) |
| Belgium | 446258.93 (393994.26-506926.83) | 600432.13 (531457.98-678239.52) | 0.35 | 8998.24 (7944.39-10221.53) | 12022.74 (10641.64-13580.72) | 0.92 (0.86-0.99) |
| Belize | 11369.89 (10012.04-12918.46) | 41505.46 (36744.3-46955.5) | 2.65 | 13357.64 (11762.39-15176.93) | 17505.55 (15497.46-19804.19) | 0.94 (0.84-1.04) |
| Benin | 259713.33 (228315.29-297548.01) | 932796.15 (817731.29-1068840.49) | 2.59 | 12964.98 (11397.58-14853.7) | 14920.07 (13079.61-17096.1) | 0.46 (0.43-0.49) |
| Bermuda | 5610.05 (4963.52-6384.81) | 5421.25 (4765.54-6213.1) | -0.03 | 16590.24 (14678.28-18881.36) | 20195.17 (17752.52-23144.97) | 0.61 (0.59-0.63) |
| Bhutan | 36014.6 (31674.27-40993.2) | 70196.62 (61963.98-80324.09) | 0.95 | 11543.89 (10152.67-13139.7) | 16042.37 (14160.93-18356.85) | 0.98 (0.9-1.06) |
| Bolivia (Plurinational State of) | 323288.49 (285747.3-366922.95) | 838273.8 (742468.9-950648.45) | 1.59 | 10810.79 (9555.41-12269.93) | 13397.11 (11865.98-15193.06) | 0.73 (0.72-0.75) |
| Bosnia and Herzegovina | 378365.53 (332942.82-431843.22) | 299580.25 (265219.58-341573.67) | -0.21 | 15804.93 (13907.55-18038.78) | 20120.68 (17812.92-22941.08) | 0.77 (0.71-0.83) |
| Botswana | 74634.79 (65668.65-84848.41) | 224933.02 (199712.42-256388.38) | 2.01 | 12383.68 (10895.98-14078.36) | 16543.58 (14688.63-18857.09) | 0.97 (0.9-1.05) |
| Brazil | 11286819.17 (10004563.12-12906287.86) | 21357006.39 (19033863.82-24391804.85) | 0.89 | 14729.24 (13055.9-16842.63) | 18415.86 (16412.65-21032.73) | 0.77 (0.75-0.8) |
| Brunei Darussalam | 15361.3 (13493.67-17520.87) | 39456.08 (34909.76-44780.51) | 1.57 | 10488.32 (9213.15-11962.83) | 14532.33 (12857.84-16493.4) | 1.13 (1.09-1.16) |
| Bulgaria | 533691.41 (467576.13-608913.44) | 431833.84 (376598.37-494997.52) | -0.19 | 12839.01 (11248.47-14648.62) | 14706.68 (12825.56-16857.8) | 0.53 (0.44-0.61) |
| Burkina Faso | 370483.24 (324000.14-420016.43) | 1118002.5 (984321.75-1278953.15) | 2.02 | 9663.95 (8451.45-10956) | 10844.95 (9548.2-12406.21) | 0.39 (0.37-0.42) |
| Burundi | 271355.33 (238491.72-308302.79) | 766782.26 (670885.91-877560.88) | 1.83 | 11188.3 (9833.29-12711.68) | 12280.95 (10745.06-14055.21) | 0.19 (0.11-0.28) |
| Cabo Verde | 19391.09 (16981.44-22160.2) | 55987.41 (49634.24-63801.27) | 1.89 | 13160.24 (11524.87-15039.56) | 17837.89 (15813.74-20327.43) | 0.92 (0.87-0.97) |
| Cambodia | 539975.53 (474574.64-613405.76) | 1131177.16 (995756.47-1287779.76) | 1.09 | 11754.56 (10330.87-13353.04) | 12546.74 (11044.68-14283.73) | 0.24 (0.18-0.29) |
| Cameroon | 602448.83 (531893.09-681301.3) | 2348535.89 (2075220.45-2662894.07) | 2.9 | 13304.95 (11746.74-15046.39) | 15223.71 (13452.02-17261.45) | 0.46 (0.42-0.49) |
| Canada | 1231321.32 (1079764.47-1409813.29) | 1593120.75 (1401678.99-1820129.41) | 0.29 | 8350.85 (7322.99-9561.39) | 9576.74 (8425.92-10941.36) | 0.38 (0.34-0.41) |
| Central African Republic | 122048.82 (107019-138366.48) | 293532.9 (257041.05-333984.78) | 1.41 | 9829.68 (8619.19-11143.88) | 11010.43 (9641.62-12527.79) | 0.35 (0.34-0.37) |
| Chad | 286676.52 (252440.94-327593.8) | 877112.24 (769849.51-1000629.73) | 2.06 | 11507.4 (10133.16-13149.85) | 11826.04 (10379.82-13491.42) | 0.11 (0.09-0.14) |
| Chile | 625560.21 (549306.64-713446.02) | 1170692.99 (1035500.58-1335937.03) | 0.87 | 8798.34 (7725.86-10034.43) | 12317.93 (10895.45-14056.62) | 1.04 (0.95-1.14) |
| China | 84743323.66 (75061753.06-96388231.31) | 122357459.14 (108104377.93-140576998.05) | 0.44 | 12707.96 (11256.13-14454.21) | 18446.77 (16297.96-21193.57) | 1.21 (0.95-1.48) |
| Colombia | 2302729.36 (2032238.5-2620197.94) | 4602821.78 (4083518.43-5222864.97) | 1 | 13613.16 (12014.08-15489.95) | 17626.45 (15637.78-20000.89) | 0.82 (0.8-0.83) |
| Comoros | 31763.48 (28146.68-36287.05) | 72937.71 (64866.41-82476.59) | 1.3 | 15505.52 (13739.96-17713.73) | 18620.35 (16559.82-21055.54) | 0.63 (0.62-0.65) |
| Congo | 108073.77 (94497.5-123235.8) | 340208.28 (300219.7-386700.14) | 2.15 | 9772.79 (8545.13-11143.85) | 12072.28 (10653.29-13722.04) | 0.86 (0.82-0.91) |
| Cook Islands | 1479.74 (1304.29-1683.58) | 1437.07 (1275.55-1633.91) | -0.03 | 15579.4 (13732.2-17725.48) | 17820.98 (15818.02-20261.98) | 0.59 (0.43-0.74) |
| Costa Rica | 218018.05 (191298.15-249774.3) | 455816.89 (401513.4-521017.54) | 1.09 | 14145.35 (12411.72-16205.74) | 18205.77 (16036.84-20809.95) | 0.73 (0.69-0.76) |
| C么te d'Ivoire | 627980.21 (548683.95-714236.11) | 1780922.33 (1571462.73-2036742.21) | 1.84 | 11286.65 (9861.47-12836.93) | 13005.19 (11475.61-14873.31) | 0.47 (0.43-0.5) |
| Croatia | 315410.09 (276815.68-363972.34) | 282432.85 (248688.23-322800.7) | -0.1 | 12919.53 (11338.66-14908.69) | 15431.32 (13587.61-17636.9) | 0.59 (0.57-0.6) |
| Cuba | 878720.71 (772282.66-991917.2) | 949974.67 (841155.5-1080911.67) | 0.08 | 14281.93 (12551.98-16121.72) | 18691.97 (16550.82-21268.33) | 0.91 (0.84-0.98) |
| Cyprus | 39326.18 (34520.27-44855.99) | 92813.42 (81021.73-107207.58) | 1.36 | 9739.16 (8548.97-11108.62) | 13142.32 (11472.62-15180.52) | 0.93 (0.91-0.96) |
| Czechia | 572868.67 (503078.72-655143.47) | 652683.38 (576987.15-748062.92) | 0.14 | 11001.88 (9661.58-12581.96) | 13836.72 (12231.98-15858.74) | 0.8 (0.74-0.86) |
| Democratic People's Republic of Korea | 1328392.17 (1169072.65-1509045.39) | 2150464.64 (1907371.95-2434085.63) | 0.62 | 12363.87 (10881.02-14045.28) | 15549.4 (13791.66-17600.18) | 0.78 (0.76-0.79) |
| Democratic Republic of the Congo | 1797550.64 (1570436.19-2063328.94) | 5216080.78 (4587550.39-5930066.04) | 1.9 | 10708.36 (9355.39-12291.65) | 12041.97 (10590.93-13690.29) | 0.35 (0.31-0.39) |
| Denmark | 212218.42 (187284.6-241164.44) | 263963.02 (233046.07-299778.9) | 0.24 | 7940.9 (7007.91-9024.02) | 10264.69 (9062.43-11657.46) | 0.82 (0.74-0.9) |
| Djibouti | 23926.7 (21030.52-27237.4) | 108514.38 (95463.61-123828.96) | 3.54 | 11503 (10110.63-13094.65) | 15713.84 (13823.97-17931.53) | 0.99 (0.96-1.02) |
| Dominica | 4929.18 (4362.86-5613.86) | 5836.08 (5177.61-6652.38) | 0.18 | 13937.98 (12336.63-15874) | 17272.61 (15323.76-19688.53) | 0.65 (0.58-0.73) |
| Dominican Republic | 421250.07 (371043.24-479741.94) | 870527.89 (769130.88-990610.41) | 1.07 | 11586.19 (10205.29-13194.97) | 14850.55 (13120.79-16899.07) | 0.76 (0.73-0.78) |
| Ecuador | 791680.01 (698544.19-893073.22) | 1868618.15 (1664831.49-2122083.18) | 1.36 | 15979.22 (14099.37-18025.73) | 19894.34 (17724.71-22592.86) | 0.7 (0.67-0.73) |
| Egypt | 7361932.2 (6524328.14-8339039.25) | 19242277.86 (17335381.55-21564033.41) | 1.61 | 27429.91 (24309.07-31070.52) | 36002.92 (32435.06-40347) | 0.91 (0.87-0.94) |
| El Salvador | 334392.17 (294141.09-382761.55) | 582798.46 (515408.16-661701.6) | 0.74 | 13364.75 (11756.02-15297.94) | 17603.84 (15568.27-19987.16) | 0.82 (0.75-0.9) |
| Equatorial Guinea | 19869.95 (17405.34-22541.81) | 105982.53 (93174.07-121803.24) | 4.33 | 10989.95 (9626.79-12467.74) | 13063.71 (11484.9-15013.82) | 0.71 (0.61-0.81) |
| Eritrea | 170569.53 (149709.93-192833.17) | 430988.98 (380281.24-489768) | 1.53 | 11065.36 (9712.14-12509.67) | 12590.39 (11109.07-14307.48) | 0.34 (0.28-0.39) |
| Estonia | 80428.32 (71131.01-91441.75) | 72458.16 (63910.98-82688.07) | -0.1 | 10591.78 (9367.4-12042.16) | 12622.72 (11133.74-14404.84) | 0.64 (0.6-0.68) |
| Eswatini | 50801.44 (44717.96-57255.73) | 105763.36 (93814.37-120258.96) | 1.08 | 14264.73 (12556.53-16077.06) | 17151 (15213.31-19501.67) | 0.53 (0.49-0.58) |
| Ethiopia | 2405914.96 (2135122.36-2729391.19) | 6815945.62 (6001834.8-7775848.3) | 1.83 | 11081.63 (9834.36-12571.56) | 12384.71 (10905.45-14128.87) | 0.32 (0.24-0.4) |
| Fiji | 67020.13 (59372.16-76426.48) | 101525.96 (90275.81-115222.29) | 0.51 | 16960.8 (15025.33-19341.26) | 21697.34 (19293.05-24624.41) | 0.8 (0.75-0.85) |
| Finland | 194550.54 (171243.03-219979.58) | 223122.04 (196951.96-254780.46) | 0.15 | 7534.27 (6631.65-8519.05) | 9538.57 (8419.78-10891.98) | 0.69 (0.65-0.72) |
| France | 2443143.56 (2159294.16-2795453.74) | 3210925.25 (2851048.01-3645161.2) | 0.31 | 8372.32 (7399.61-9579.64) | 11350.56 (10078.4-12885.58) | 0.99 (0.95-1.04) |
| Gabon | 47434.34 (41840.21-54309.61) | 127742.55 (112129.91-145249.33) | 1.69 | 10606.64 (9355.75-12143.99) | 13772.96 (12089.63-15660.51) | 0.81 (0.78-0.84) |
| Gambia | 58889.14 (51813.31-66747.65) | 172818.13 (151540.51-197159.13) | 1.93 | 13285.17 (11688.89-15058.02) | 14570.6 (12776.65-16622.84) | 0.29 (0.29-0.3) |
| Georgia | 432265.44 (382878.53-493916.89) | 298255.61 (260960.87-341649.78) | -0.31 | 16118.31 (14276.77-18417.16) | 18643.45 (16312.22-21355.95) | 0.54 (0.51-0.57) |
| Germany | 3381067.21 (2969319.12-3864306.92) | 4113372.5 (3648271.86-4674846.76) | 0.22 | 8476.91 (7444.59-9688.47) | 11561.62 (10254.34-13139.78) | 1.05 (1.01-1.1) |
| Ghana | 812554.28 (714293.95-923719.74) | 2673046.17 (2358109.34-3057496.94) | 2.29 | 11884.02 (10446.91-13509.87) | 15250.76 (13453.92-17444.2) | 0.71 (0.67-0.74) |
| Greece | 459220.21 (405169.49-521899.71) | 547243.74 (481633.04-626453.06) | 0.19 | 9092.89 (8022.65-10333.99) | 12644.18 (11128.23-14474.32) | 1.16 (1.1-1.22) |
| Greenland | 2860.01 (2500.96-3304.43) | 2578.12 (2261.01-2938.14) | -0.1 | 8546.2 (7473.28-9874.18) | 9740.65 (8542.58-11100.89) | 0.4 (0.35-0.44) |
| Grenada | 4755.85 (4194.2-5413.49) | 8485.43 (7521.33-9623.04) | 0.78 | 12153.37 (10718.1-13833.93) | 15897.5 (14091.25-18028.82) | 0.87 (0.84-0.91) |
| Guam | 11164.11 (9885.2-12798.09) | 12957.42 (11515.35-14679.06) | 0.16 | 14437.16 (12783.3-16550.18) | 17350.59 (15419.59-19655.94) | 0.58 (0.49-0.67) |
| Guatemala | 507670.57 (448595.62-577154.18) | 1456798.42 (1285672.12-1656968.86) | 1.87 | 14353 (12682.82-16317.46) | 17315.14 (15281.17-19694.31) | 0.66 (0.62-0.7) |
| Guinea | 374976.98 (331099.18-426148) | 940776.36 (830201.52-1078239.59) | 1.51 | 14894.18 (13151.34-16926.7) | 15343.37 (13539.98-17585.29) | 0.06 (0-0.11) |
| Guinea-Bissau | 53275.23 (47398.18-60357) | 136012.13 (119193.09-154954.42) | 1.55 | 12193.57 (10848.44-13814.44) | 13599.05 (11917.41-15492.97) | 0.33 (0.29-0.38) |
| Guyana | 47491.95 (41624.42-54388.18) | 58567.02 (51776.94-66712.09) | 0.23 | 11785.88 (10329.75-13497.28) | 14647.33 (12949.16-16684.37) | 0.64 (0.58-0.71) |
| Haiti | 366513.33 (322344.31-415310.33) | 967569.58 (854414.09-1101247.43) | 1.64 | 12489.53 (10984.4-14152.37) | 14114.05 (12463.44-16064.03) | 0.45 (0.4-0.5) |
| Honduras | 314295.46 (274696.21-358113.65) | 1006415.23 (894008.52-1142631.04) | 2.2 | 15344.61 (13411.29-17483.92) | 18589.41 (16513.16-21105.44) | 0.6 (0.58-0.63) |
| Hungary | 642101.12 (564688.81-742369.21) | 683349.29 (600185.73-781158.5) | 0.06 | 12616.22 (11095.19-14586.32) | 15740.04 (13824.48-17992.94) | 0.74 (0.68-0.79) |
| Iceland | 11821.05 (10462.37-13464.43) | 19542.76 (17284.07-22137.97) | 0.65 | 8924.98 (7899.16-10165.74) | 11862.76 (10491.7-13438.09) | 0.89 (0.84-0.94) |
| India | 51697719.81 (45754136.94-58489476.44) | 114492832.48 (101718430.81-129313582.58) | 1.21 | 12297.31 (10883.52-13912.87) | 14684.7 (13046.27-16585.59) | 0.53 (0.4-0.65) |
| Indonesia | 13827589.39 (12253123.52-15712194.84) | 28963591.36 (25819979.13-33040745.74) | 1.09 | 14629.22 (12963.48-16623.09) | 18888.15 (16838.09-21547) | 0.89 (0.88-0.91) |
| Iran (Islamic Republic of) | 5786254.71 (5151867.51-6529922.77) | 17808377.58 (15922600.92-20091896.82) | 2.08 | 22664.64 (20179.76-25577.57) | 37572.23 (33593.6-42390.01) | 1.78 (1.58-1.99) |
| Iraq | 1848923.15 (1635127.63-2094144.02) | 6390889.79 (5755729.29-7169539.6) | 2.46 | 21840.28 (19314.84-24736.94) | 28847.84 (25980.79-32362.59) | 1.02 (0.97-1.07) |
| Ireland | 158730.23 (140193.44-179240.18) | 288420.76 (254322.91-327628.93) | 0.82 | 8910.02 (7869.49-10061.31) | 12493.13 (11016.16-14191.45) | 1.15 (1.1-1.2) |
| Israel | 265059.1 (233168.58-300768.29) | 665516.39 (593610.63-754531.78) | 1.51 | 10920.23 (9606.37-12391.42) | 14811.08 (13210.82-16792.12) | 1.03 (0.97-1.09) |
| Italy | 3294440.87 (2933545.87-3728870.35) | 4192911.29 (3719863.79-4756993.64) | 0.27 | 11470.37 (10213.83-12982.94) | 17037.98 (15115.74-19330.14) | 1.44 (1.28-1.59) |
| Jamaica | 151913.57 (133603.98-173300.16) | 263024.34 (231762.86-300546.95) | 0.73 | 13049.28 (11476.5-14886.38) | 17184.14 (15141.74-19635.6) | 0.82 (0.72-0.91) |
| Japan | 5401919.87 (4787537.52-6119944.81) | 4861619.38 (4311194.19-5511431.86) | -0.1 | 8321.31 (7374.9-9427.39) | 9592.89 (8506.8-10875.09) | 0.45 (0.39-0.51) |
| Jordan | 401714.31 (354444.9-454647.17) | 2226367.32 (2006068.55-2489420.02) | 4.54 | 22544.88 (19892.04-25515.56) | 32542.27 (29322.22-36387.25) | 1.3 (1.26-1.34) |
| Kazakhstan | 1036555.02 (908507.19-1182344) | 1546115.04 (1366214.53-1773172.79) | 0.49 | 12615.21 (11056.83-14389.51) | 16525.99 (14603.08-18952.94) | 0.75 (0.65-0.85) |
| Kenya | 1231953.38 (1086569.85-1397864.51) | 3896215.55 (3456335.78-4437753.26) | 2.16 | 12130.91 (10699.33-13764.61) | 14902.69 (13220.18-16974.02) | 0.71 (0.66-0.76) |
| Kiribati | 5576.86 (4935.55-6366.14) | 11359.5 (10032.14-12942.24) | 1.04 | 15207.27 (13458.53-17359.54) | 18301.18 (16162.68-20851.11) | 0.44 (0.36-0.53) |
| Kuwait | 318491.41 (283061.54-361696.7) | 1285355.05 (1143673.5-1449005.25) | 3.04 | 31085.42 (27627.39-35302.34) | 42204.49 (37552.39-47577.92) | 1.07 (1.05-1.09) |
| Kyrgyzstan | 301960.37 (264521.92-344330.88) | 586369.59 (516704.53-671740.82) | 0.94 | 14415.04 (12627.8-16437.74) | 17056.21 (15029.81-19539.47) | 0.46 (0.42-0.51) |
| Lao People's Democratic Republic | 182037.03 (160415.96-206207.01) | 461658.85 (406463.62-523859.93) | 1.54 | 9795.19 (8631.79-11095.75) | 11534.75 (10155.67-13088.87) | 0.56 (0.5-0.62) |
| Latvia | 133544.56 (118018.07-152480.09) | 98493.54 (86468.57-112594.58) | -0.26 | 10389.81 (9181.84-11863) | 12495.13 (10969.61-14284.02) | 0.63 (0.58-0.67) |
| Lebanon | 337398.34 (299832.74-379800.13) | 990385.27 (885204.85-1118110.51) | 1.94 | 23464.13 (20851.66-26412.93) | 32858.15 (29368.57-37095.71) | 1.06 (1.01-1.1) |
| Lesotho | 76813.8 (68013.74-87475.12) | 136477.25 (120984.55-154823.33) | 0.78 | 11579.09 (10252.55-13186.2) | 13588.93 (12046.33-15415.63) | 0.5 (0.48-0.53) |
| Liberia | 145885.99 (128663.04-165879.58) | 427184.32 (377969.74-487175.17) | 1.93 | 13471.26 (11880.87-15317.48) | 15236.4 (13481.06-17376.1) | 0.52 (0.37-0.66) |
| Libya | 466800.26 (414752.9-528290.49) | 1408230.89 (1262404.47-1588954.29) | 2.02 | 23585.49 (20955.76-26692.34) | 34157.87 (30620.73-38541.48) | 1.41 (1.34-1.48) |
| Lithuania | 179164.18 (159406.83-204297.75) | 133063.22 (117702.46-151647.53) | -0.26 | 9786.22 (8707.04-11159.05) | 11517.69 (10188.09-13126.31) | 0.51 (0.49-0.53) |
| Luxembourg | 17894.18 (15687.58-20498.48) | 38152.61 (33474.62-43529.74) | 1.13 | 8997.59 (7888.06-10307.09) | 12023.51 (10549.28-13718.08) | 0.94 (0.91-0.97) |
| Madagascar | 594978.59 (522314.81-672291.4) | 1809406.19 (1583164.84-2044016.15) | 2.04 | 11217.45 (9847.48-12675.06) | 12815.22 (11212.85-14476.86) | 0.41 (0.39-0.43) |
| Malawi | 537544.16 (475299.09-608082.6) | 1296165.47 (1138956.06-1457423.1) | 1.41 | 12212.39 (10798.25-13814.94) | 13388.78 (11764.88-15054.5) | 0.34 (0.32-0.36) |
| Malaysia | 1562992.33 (1369061.81-1771765.56) | 3840815.28 (3387914.65-4362835.28) | 1.46 | 17349.89 (15197.18-19667.36) | 21639.21 (19087.56-24580.28) | 0.63 (0.59-0.67) |
| Maldives | 12808.81 (11306.29-14514.63) | 63357.48 (55388.1-72768.9) | 3.95 | 13451.05 (11873.19-15242.4) | 18714.87 (16360.84-21494.87) | 0.98 (0.87-1.1) |
| Mali | 544891.73 (476267.55-618364.98) | 1672559.54 (1475000.56-1900430.12) | 2.07 | 15059.88 (13163.23-17090.56) | 15923.72 (14042.84-18093.17) | 0.2 (0.19-0.21) |
| Malta | 19631.63 (17253.07-22260.4) | 26772.41 (23468.39-30367.88) | 0.36 | 10246.55 (9005.08-11618.61) | 13757.99 (12060.1-15605.66) | 0.9 (0.88-0.93) |
| Marshall Islands | 2477.52 (2184.2-2818.62) | 4739.07 (4199.69-5367.32) | 0.91 | 12342.87 (10881.53-14042.17) | 15542.25 (13773.28-17602.67) | 0.81 (0.79-0.83) |
| Mauritania | 153952.56 (135975.68-175769.95) | 404671.74 (360197.13-459153.66) | 1.63 | 16885.13 (14913.47-19278) | 19678.33 (17515.63-22327.67) | 0.45 (0.43-0.47) |
| Mauritius | 76896.88 (67796.8-87862.68) | 105978.96 (93058.57-121080.53) | 0.38 | 12702.1 (11198.92-14513.47) | 16564.4 (14544.95-18924.76) | 0.84 (0.77-0.91) |
| Mexico | 6128403.05 (5474327.15-6929133.99) | 12600704.95 (11226255.98-14314500.39) | 1.06 | 14408.41 (12870.62-16291) | 18404.13 (16396.66-20907.24) | 0.85 (0.84-0.86) |
| Micronesia (Federated States of) | 6062.94 (5369.21-6876.48) | 8514.12 (7529.38-9652.82) | 0.4 | 12807.12 (11341.71-14525.59) | 15809.42 (13980.91-17923.82) | 0.66 (0.61-0.72) |
| Monaco | 1404.87 (1240.81-1597.79) | 1801.35 (1580.69-2066.14) | 0.28 | 10189.94 (9000-11589.26) | 12820.1 (11249.68-14704.56) | 0.75 (0.69-0.81) |
| Mongolia | 124773.13 (110514.5-142107.9) | 257293.39 (226530.37-292775.5) | 1.06 | 12150.55 (10762.02-13838.63) | 15254.73 (13430.81-17358.44) | 0.9 (0.86-0.94) |
| Montenegro | 40250.98 (35507.77-45929.96) | 46187.43 (40956.76-53069.34) | 0.15 | 12620.24 (11133.06-14400.82) | 15814.49 (14023.52-18170.84) | 0.73 (0.69-0.77) |
| Morocco | 2648103.69 (2347252.51-3005740.21) | 5861566.72 (5224784.45-6604699.94) | 1.21 | 21278.54 (18861.08-24152.28) | 30174.08 (26896.06-33999.57) | 1.18 (1.16-1.21) |
| Mozambique | 617131.41 (541979.41-703232.75) | 1622380.81 (1420734.32-1838876.4) | 1.63 | 10674.44 (9374.54-12163.72) | 11375.48 (9961.62-12893.46) | 0.21 (0.18-0.23) |
| Myanmar | 2784639.43 (2459265.27-3182872.65) | 4767472.89 (4243555.51-5407299.25) | 0.71 | 13638.82 (12045.18-15589.32) | 16216.1 (14434.05-18392.41) | 0.67 (0.63-0.71) |
| Namibia | 73131.24 (63945.32-82468.98) | 172093.79 (152161.52-197731.53) | 1.35 | 11107.22 (9712.06-12525.45) | 13333.54 (11789.22-15319.91) | 0.56 (0.53-0.59) |
| Nauru | 666.68 (589.83-758.35) | 834.44 (736.5-944.98) | 0.25 | 13420.59 (11873.64-15266.08) | 14527.16 (12822.03-16451.48) | 0.25 (0.19-0.3) |
| Nepal | 997629.78 (877856.78-1129166.41) | 2103318.06 (1853884.53-2396375.46) | 1.11 | 11207.99 (9862.39-12685.76) | 12635.77 (11137.28-14396.32) | 0.43 (0.39-0.48) |
| Netherlands | 740797.8 (650428.57-839514.32) | 858813.31 (761241.23-977522.87) | 0.16 | 9138.88 (8024.03-10356.7) | 11598.19 (10280.49-13201.35) | 0.76 (0.68-0.84) |
| New Zealand | 152242.61 (135165.79-174448.79) | 274737.19 (243680.41-312332.63) | 0.8 | 8436.84 (7490.49-9667.44) | 11293.17 (10016.57-12838.54) | 0.85 (0.8-0.9) |
| Nicaragua | 231989.71 (204981.65-262464.2) | 630182.75 (558528.21-715544.06) | 1.72 | 13420.59 (11858.17-15183.53) | 17543.18 (15548.44-19919.49) | 0.88 (0.86-0.9) |
| Niger | 455645.23 (402889.56-519269.78) | 1444651.18 (1268527.69-1639316.5) | 2.17 | 13608.16 (12032.58-15508.35) | 13928.33 (12230.27-15805.16) | 0.11 (0.06-0.16) |
| Nigeria | 5734394.26 (5091047.45-6514367.61) | 16727829.95 (14848402.87-18878763.17) | 1.92 | 13980.66 (12412.16-15882.27) | 15507.89 (13765.53-17501.96) | 0.33 (0.32-0.35) |
| Niue | 153.24 (135.7-174.06) | 140.27 (124.12-158.73) | -0.08 | 14897.75 (13192.29-16921.26) | 17942.39 (15876.6-20303.92) | 0.67 (0.62-0.72) |
| North Macedonia | 136079.89 (118531.7-156229.69) | 192083.99 (169675.95-219758.06) | 0.41 | 13178.15 (11478.76-15129.48) | 17450.85 (15415.08-19965.04) | 0.89 (0.85-0.94) |
| Northern Mariana Islands | 5185.96 (4550.21-5916.51) | 4866.57 (4301.58-5556.33) | -0.06 | 17862.28 (15672.51-20378.53) | 20518.26 (18136.17-23426.4) | 0.43 (0.25-0.61) |
| Norway | 177677.43 (157913.18-201655.62) | 272730.07 (242710.85-308819.44) | 0.53 | 8200.87 (7288.63-9307.6) | 10921.32 (9719.21-12366.49) | 0.88 (0.83-0.92) |
| Oman | 231939.31 (204940.44-265073.93) | 1055228.69 (933886.04-1196787.06) | 3.55 | 23310.93 (20597.42-26641.1) | 35244.85 (31191.98-39972.93) | 1.37 (1.27-1.48) |
| Pakistan | 6478286.66 (5743810.3-7333203.21) | 19263101.05 (17099533.16-21819140.22) | 1.97 | 13102.05 (11616.6-14831.08) | 15790.64 (14017.08-17885.91) | 0.63 (0.59-0.67) |
| Palau | 1270.92 (1126.54-1446.03) | 1826 (1610.78-2079.39) | 0.44 | 14835.91 (13150.57-16880.04) | 20142.85 (17768.78-22938.05) | 0.88 (0.77-1) |
| Palestine | 195176.14 (172258.63-221255.31) | 759625.96 (677976.07-856278.03) | 2.89 | 21999.86 (19416.64-24939.45) | 28608.67 (25533.61-32248.73) | 0.79 (0.77-0.82) |
| Panama | 170560.66 (150263-194735) | 379492.99 (337089.81-432732.74) | 1.22 | 13843.29 (12195.86-15805.37) | 17533.26 (15574.16-19993.03) | 0.79 (0.78-0.81) |
| Papua New Guinea | 226834.98 (199501.11-257954.41) | 729245.86 (647355.15-832768.81) | 2.21 | 11411.75 (10036.62-12977.33) | 13656.1 (12122.59-15594.71) | 0.63 (0.61-0.65) |
| Paraguay | 224357.94 (196701.54-256336.01) | 561185.77 (493370.94-639055.66) | 1.5 | 11866.79 (10403.98-13558.18) | 14543.08 (12785.66-16561.07) | 0.58 (0.51-0.66) |
| Peru | 1157665.18 (1017278.19-1319796.79) | 2709660.22 (2399986.81-3088931.18) | 1.34 | 10828.78 (9515.61-12345.36) | 14019.02 (12416.85-15981.26) | 0.94 (0.91-0.96) |
| Philippines | 3577899.35 (3174744.06-4069800.27) | 8091588.36 (7166508.97-9172131.96) | 1.26 | 11501.46 (10205.49-13082.72) | 13478.49 (11937.54-15278.39) | 0.51 (0.49-0.54) |
| Poland | 2238231.24 (1968107.73-2552904.18) | 2529626.14 (2225647.6-2869962.03) | 0.13 | 11802.61 (10378.2-13461.94) | 14114.26 (12418.18-16013.19) | 0.58 (0.51-0.66) |
| Portugal | 477531.22 (420904.82-543590.2) | 641885.42 (562753.56-740053.12) | 0.34 | 9548.7 (8416.4-10869.61) | 14014.89 (12287.13-16158.28) | 1.28 (1.21-1.35) |
| Puerto Rico | 306054.21 (271564.71-346497.54) | 298990.3 (265605.16-340573.62) | -0.02 | 16590.22 (14720.66-18782.53) | 20409.57 (18130.65-23248.12) | 0.7 (0.65-0.76) |
| Qatar | 86878.73 (76300.12-99270.04) | 904451.54 (801927.96-1023975.73) | 9.41 | 29808.49 (26178.92-34060) | 41241.05 (36566.2-46691.1) | 1.05 (1.02-1.08) |
| Republic of Korea | 2401598.09 (2118191.22-2730246.24) | 2988094 (2627894.13-3408366.04) | 0.24 | 9287.11 (8191.16-10558.01) | 12303.59 (10820.45-14034.08) | 1.29 (1.02-1.56) |
| Republic of Moldova | 239291.89 (210265.03-273790.2) | 249461.67 (219298.65-284947.24) | 0.04 | 10891.43 (9570.26-12461.63) | 13937.17 (12251.99-15919.71) | 0.81 (0.7-0.93) |
| Romania | 1316048.17 (1155189.47-1502319.28) | 1213186.32 (1062375.52-1391716.13) | -0.08 | 11607.36 (10188.61-13250.24) | 14562.77 (12752.48-16705.8) | 0.86 (0.82-0.91) |
| Russian Federation | 8715886.38 (7716010.26-9958834.09) | 9625391.88 (8443051.94-10952978.75) | 0.1 | 11743.16 (10396-13417.82) | 14273.03 (12519.8-16241.65) | 0.54 (0.48-0.61) |
| Rwanda | 291565.51 (254348.27-333872.31) | 727767.27 (638213.3-833052.88) | 1.5 | 9238.76 (8059.47-10579.32) | 10622.48 (9315.35-12159.22) | 0.42 (0.31-0.54) |
| Saint Kitts and Nevis | 2667.81 (2338.95-3055.57) | 5756.36 (5064.96-6571.24) | 1.16 | 13408.81 (11755.9-15357.75) | 18222.04 (16033.36-20801.56) | 0.9 (0.85-0.96) |
| Saint Lucia | 7602.98 (6696-8697.68) | 14492.4 (12762.46-16386.06) | 0.91 | 11418.02 (10055.94-13062.03) | 15669.35 (13798.93-17716.8) | 1.01 (0.97-1.04) |
| Saint Vincent and the Grenadines | 6633.76 (5847.52-7602.02) | 9271.16 (8139.91-10616.53) | 0.4 | 12420.41 (10948.32-14233.28) | 16414.46 (14411.6-18796.42) | 0.98 (0.93-1.03) |
| Samoa | 10597.01 (9365.95-12072.34) | 15817.8 (14054.35-17955.48) | 0.49 | 13527.4 (11955.91-15410.7) | 15771.82 (14013.5-17903.29) | 0.44 (0.33-0.54) |
| San Marino | 1157.62 (1012.83-1321.42) | 1702.53 (1503.36-1940.62) | 0.47 | 9298.96 (8135.9-10614.76) | 12394.36 (10944.39-14127.63) | 1.01 (0.92-1.1) |
| Sao Tome and Principe | 6698.5 (5889.15-7627.9) | 18201.13 (16107.33-20847.57) | 1.72 | 13393.29 (11775.04-15251.58) | 16122.63 (14267.94-18466.87) | 0.65 (0.6-0.7) |
| Saudi Arabia | 1825513.33 (1628183.45-2060038.91) | 9260902.36 (8221290.79-10486451.17) | 4.07 | 22763.11 (20302.52-25687.51) | 36604.55 (32495.4-41448.65) | 1.53 (1.49-1.57) |
| Senegal | 508840.64 (446831.48-580429.06) | 1376209.39 (1210322.18-1564547.89) | 1.7 | 15586.31 (13686.91-17779.14) | 17789.39 (15645.07-20223.92) | 0.44 (0.43-0.44) |
| Serbia | 751914.78 (659598.14-853495.76) | 817919.17 (727439.1-932273.15) | 0.09 | 15782.89 (13845.14-17915.1) | 19289.92 (17156.02-21986.86) | 0.74 (0.7-0.77) |
| Seychelles | 5203.79 (4611.86-5885.9) | 9848.27 (8694.67-11246.88) | 0.89 | 14048.15 (12450.18-15889.58) | 18193.44 (16062.3-20777.18) | 0.81 (0.74-0.89) |
| Sierra Leone | 238599.44 (209209.7-271873.65) | 608818.93 (536079.02-698568.38) | 1.55 | 12556.28 (11009.65-14307.34) | 13691.66 (12055.82-15710.02) | 0.15 (0.1-0.21) |
| Singapore | 243153.8 (214144.23-278388.38) | 495406.78 (433876.25-568847.75) | 1.04 | 12787.55 (11261.93-14640.55) | 16610.22 (14547.19-19072.58) | 0.78 (0.73-0.83) |
| Slovakia | 275987.87 (243710.46-315146.63) | 343261.63 (302541.97-392922.52) | 0.24 | 10330.01 (9121.89-11795.69) | 13272.03 (11697.63-15192.14) | 0.78 (0.71-0.85) |
| Slovenia | 114515.07 (100059.7-131215) | 126388.52 (111333.12-144790.05) | 0.1 | 11290.25 (9865.07-12936.73) | 14407.19 (12691-16504.8) | 0.85 (0.83-0.86) |
| Solomon Islands | 17731.94 (15706.65-20079.82) | 49494.91 (43814.45-56006.27) | 1.79 | 11702.16 (10365.57-13251.64) | 14372.5 (12722.99-16263.29) | 0.79 (0.72-0.86) |
| Somalia | 490678.32 (435068.79-556787.55) | 1472102.53 (1301938.47-1669646.58) | 2 | 14044.74 (12453.02-15936.99) | 14775.92 (13067.93-16758.73) | 0.28 (0.21-0.35) |
| South Africa | 2708969.01 (2406869.67-3065868.59) | 5764172.6 (5113833.96-6534769.99) | 1.13 | 14358.76 (12757.5-16250.49) | 18502.3 (16414.79-20975.82) | 0.8 (0.74-0.86) |
| South Sudan | 347070.34 (305771.58-396200.43) | 662155.55 (584679.25-750544.94) | 0.91 | 12854.57 (11324.98-14674.22) | 14883.95 (13142.44-16870.77) | 0.55 (0.52-0.57) |
| Spain | 1823416.35 (1614222-2077973.85) | 2834356.54 (2499167.67-3242677.45) | 0.55 | 9388.74 (8311.61-10699.46) | 14179.22 (12502.4-16221.9) | 1.38 (1.31-1.45) |
| Sri Lanka | 1438751.99 (1283361.22-1638022.39) | 1992033.62 (1763761.56-2267363.58) | 0.38 | 15663.88 (13972.12-17833.36) | 18068.28 (15997.79-20565.59) | 0.48 (0.47-0.5) |
| Sudan | 1375535.08 (1210889.32-1550691.25) | 4367589.27 (3863922.08-4963862.15) | 2.18 | 15095.3 (13288.46-17017.49) | 19534.46 (17281.76-22201.34) | 0.82 (0.77-0.87) |
| Suriname | 24301.45 (21464.76-27648.91) | 46535.43 (41073.97-53266.18) | 0.91 | 12291.2 (10856.46-13984.28) | 16203.12 (14301.5-18546.69) | 0.94 (0.89-1) |
| Sweden | 374511.82 (330077.6-425746.22) | 517398.17 (459597.14-589930.6) | 0.38 | 8912.32 (7854.91-10131.56) | 11429.82 (10152.94-13032.13) | 0.82 (0.79-0.85) |
| Switzerland | 321206.48 (282743.33-365564.49) | 464794.49 (409965.68-527762.98) | 0.45 | 8853.72 (7793.52-10076.4) | 11604.53 (10235.62-13176.66) | 0.87 (0.86-0.89) |
| Syrian Arab Republic | 1233396.14 (1094687.48-1398750.28) | 2121560.95 (1899353.95-2387179.48) | 0.72 | 22012.06 (19536.57-24963.09) | 29884.64 (26754.59-33626.18) | 1.2 (1.12-1.28) |
| Taiwan (Province of China) | 1577590.08 (1391204.04-1799302.03) | 2231395.7 (1954859.67-2549830.41) | 0.41 | 14013.51 (12357.87-15982.94) | 19621.31 (17189.65-22421.4) | 1.25 (1.18-1.32) |
| Tajikistan | 321160.91 (284022.88-367324.66) | 840952.31 (744643.94-959836.8) | 1.62 | 13324.03 (11783.28-15239.23) | 16358.75 (14485.3-18671.37) | 0.63 (0.55-0.72) |
| Thailand | 4007128.21 (3549131.23-4543249.1) | 5496240.05 (4844572.92-6278375.91) | 0.37 | 12657.96 (11211.21-14351.49) | 17220.04 (15178.33-19670.52) | 1.04 (0.98-1.1) |
| Timor-Leste | 51695.66 (45491.13-59042.38) | 92537.48 (82277.8-104963.13) | 0.79 | 13503.6 (11882.89-15422.66) | 13418.81 (11931.06-15220.65) | -0.08 (-0.1--0.05) |
| Togo | 190819.44 (168317.17-216587.51) | 611380.41 (538196.62-697847.69) | 2.2 | 11865.89 (10466.62-13468.25) | 14709.01 (12948.31-16789.3) | 0.71 (0.7-0.73) |
| Tokelau | 92.46 (81.64-104.71) | 109.9 (96.8-124.64) | 0.19 | 13230.06 (11682.28-14982.88) | 16728.57 (14734.66-18972.97) | 0.76 (0.71-0.8) |
| Tonga | 6377.06 (5639.78-7244.35) | 8489.32 (7506.38-9659.78) | 0.33 | 14402.53 (12737.4-16361.3) | 17254.96 (15257.09-19633.99) | 0.57 (0.47-0.66) |
| Trinidad and Tobago | 82593.76 (72705.81-94496.08) | 119169.21 (105069.37-136222.78) | 0.44 | 13351.31 (11752.92-15275.33) | 17269.33 (15226.06-19740.64) | 0.89 (0.85-0.93) |
| Tunisia | 866725.5 (767881.05-974298.08) | 1868304.65 (1670876.85-2111026.31) | 1.16 | 21156.88 (18744.07-23782.74) | 30909.86 (27643.55-34925.53) | 1.3 (1.28-1.33) |
| Turkey | 6341668.47 (5640315.24-7183563.24) | 13357849.83 (12049883.46-15076936.97) | 1.11 | 21880.02 (19460.21-24784.72) | 30403.59 (27426.54-34316.37) | 1.13 (1.1-1.15) |
| Turkmenistan | 248704.35 (218569.29-284388.61) | 476552.5 (420776.17-541740.94) | 0.92 | 14145.25 (12431.29-16174.81) | 17803.38 (15719.65-20238.73) | 0.77 (0.75-0.79) |
| Tuvalu | 608.48 (535.81-693.86) | 971.02 (858.56-1099.91) | 0.6 | 13472.42 (11863.42-15362.66) | 15537.65 (13738.1-17600.03) | 0.39 (0.33-0.46) |
| Uganda | 688595.87 (607246.81-778401.27) | 2094281.31 (1834616.76-2371395.22) | 2.04 | 9267.59 (8172.74-10476.25) | 10418.64 (9126.86-11797.22) | 0.39 (0.36-0.41) |
| Ukraine | 2904821.75 (2563952.16-3358254.26) | 2795649.79 (2466605.8-3182696.97) | -0.04 | 11641.81 (10275.69-13459.06) | 13821.92 (12195.1-15735.52) | 0.57 (0.51-0.62) |
| United Arab Emirates | 323160.81 (287211.78-369947.35) | 2842839.26 (2485888.24-3249619.1) | 7.8 | 27416.7 (24366.82-31386.04) | 41415.92 (36215.68-47342.1) | 1.47 (1.3-1.63) |
| United Kingdom | 2541144.32 (2268848.92-2876035.42) | 3745060.56 (3339857.55-4245077.18) | 0.47 | 8928.52 (7971.78-10105.18) | 12341.81 (11006.47-13989.61) | 0.98 (0.94-1.03) |
| United Republic of Tanzania | 1362391.15 (1196678.51-1549142.8) | 3990793.39 (3517942.43-4548986.14) | 1.93 | 12048.72 (10583.19-13700.32) | 14152.81 (12475.91-16132.37) | 0.54 (0.51-0.56) |
| United States of America | 11550618.89 (10219699.15-13107974.88) | 17225817.73 (15306637.58-19488473.67) | 0.49 | 8604.14 (7612.72-9764.22) | 11332.78 (10070.16-12821.37) | 0.96 (0.89-1.03) |
| United States Virgin Islands | 7581.89 (6660.24-8734.33) | 5884.1 (5178.3-6694.69) | -0.22 | 13817.94 (12138.25-15918.25) | 17407.23 (15319.22-19805.23) | 0.75 (0.71-0.78) |
| Uruguay | 134580.46 (119196.03-152961.4) | 189134.81 (168810.65-216662.08) | 0.41 | 9057.87 (8022.42-10294.98) | 11524.71 (10286.28-13202.05) | 0.8 (0.77-0.83) |
| Uzbekistan | 1378293.76 (1208170.98-1584126.85) | 3277595.77 (2902008.98-3735974.09) | 1.38 | 14092.83 (12353.35-16197.44) | 18378.45 (16272.42-20948.71) | 0.78 (0.71-0.85) |
| Vanuatu | 10341.37 (9121.63-11784.34) | 28237.69 (25100.51-32000.3) | 1.73 | 14725.58 (12988.74-16780.3) | 18097.15 (16086.57-20508.55) | 0.69 (0.68-0.7) |
| Venezuela (Bolivarian Republic of) | 1292828.96 (1132928.71-1478416.25) | 2368461.73 (2088661.58-2710946.07) | 0.83 | 13481.07 (11813.7-15416.3) | 18012.82 (15884.86-20617.51) | 0.84 (0.78-0.9) |
| Viet Nam | 3902243.86 (3445617.04-4464043.65) | 7794821.76 (6881440.77-8944180.22) | 1 | 11902.39 (10509.62-13615.96) | 14967.46 (13213.61-17174.44) | 0.74 (0.71-0.77) |
| Yemen | 994129.98 (879091.55-1122264.15) | 3798275.22 (3383480.37-4297494.01) | 2.82 | 18268.65 (16154.65-20623.31) | 22710.71 (20230.56-25695.65) | 0.84 (0.76-0.92) |
| Zambia | 384445.56 (339760.92-436803.13) | 1275045.92 (1128204.93-1448109.57) | 2.32 | 10881.83 (9617.02-12363.83) | 13197.3 (11677.43-14988.59) | 0.54 (0.49-0.6) |
| Zimbabwe | 528879.25 (465924.65-600065.88) | 1060186.64 (933567.26-1200428.55) | 1 | 11472.5 (10106.88-13016.69) | 13687.88 (12053.12-15498.52) | 0.55 (0.5-0.6) |
