# Supplementary material for: Global, regional and national burden of Metabolic dysfunction-associated steatotic liver disease in adolescents and adults aged 15–49 years from 1990 to 2021: results from the 2021 Global Burden of Disease study
Source: Front Med (Lausanne). 2025 Jun 25;12:1568211. doi: 10.3389/fmed.2025.1568211 (PMC12237898; doi:10.3389/fmed.2025.1568211)
Supplement: Supplementary file 1 [file Supplementary_file_1.ZIP › Supplementary Table 5.docx]

**Supplementary Table 5** The incidence of MASLD cases and rates among the adolescents and adults aged 15-49 years in 1990 and 2021 across 204 countries, and the trends from 1990 to 2021.

| **location** | **incidence cases** | | | **incidence rates** | | |
| --- | --- | --- | --- | --- | --- | --- |
|  | **1990**  **(95%UI)** | **2021**  **(95%UI)** | **percentage**  **Change**  **(100%)** | **1990**  **Per 100,000**  **(95%)** | **2021**  **Per 100,000**  **(95%UI)** | **EAPC**  **(95% CI)** |
| Afghanistan | 49190.44 (43025.9-56018.35) | 216935.24 (189459.28-245514.09) | 3.41 | 1229.99 (1075.85-1400.72) | 1467.85 (1281.94-1661.22) | 0.54 (0.47-0.61) |
| Albania | 13646.03 (11991.38-15554.7) | 11355.39 (10020.92-12815.4) | -0.17 | 797.4 (700.71-908.93) | 901.69 (795.73-1017.63) | 0.49 (0.43-0.55) |
| Algeria | 161455.6 (140806.13-182345.18) | 360598.85 (321810.51-403629.69) | 1.23 | 1371.63 (1196.2-1549.09) | 1592.37 (1421.08-1782.39) | 0.58 (0.54-0.61) |
| American Samoa | 306.85 (268.76-347.4) | 326.78 (289.06-364.77) | 0.06 | 1260.3 (1103.87-1426.86) | 1362.74 (1205.47-1521.18) | 0.23 (0.19-0.27) |
| Andorra | 146.93 (129.6-167.07) | 214.39 (189.28-240.53) | 0.46 | 453.27 (399.81-515.41) | 519.83 (458.96-583.22) | 0.41 (0.35-0.48) |
| Angola | 28645.46 (24818.17-32931.5) | 103122.11 (90412.7-117683.4) | 2.6 | 615.44 (533.21-707.52) | 703.3 (616.62-802.61) | 0.44 (0.39-0.49) |
| Antigua and Barbuda | 241.97 (212.28-276.44) | 412.72 (361.07-464.53) | 0.71 | 769.97 (675.5-879.64) | 871 (762-980.33) | 0.45 (0.42-0.49) |
| Argentina | 70487.2 (61702.76-80301.49) | 128379.22 (113248.21-145072.66) | 0.82 | 443.48 (388.22-505.23) | 545.36 (481.08-616.27) | 0.68 (0.63-0.73) |
| Armenia | 14123.98 (12288.75-16065.14) | 13462.26 (11872.05-15171.59) | -0.05 | 827.32 (719.82-941.03) | 924.52 (815.31-1041.91) | 0.58 (0.51-0.66) |
| Australia | 37554.99 (32883.08-42223.59) | 61823.46 (54285.33-69708.21) | 0.65 | 417.9 (365.91-469.85) | 514.87 (452.09-580.54) | 0.75 (0.71-0.79) |
| Austria | 18270.6 (15990.64-20665.44) | 22530.3 (19844.6-25391.87) | 0.23 | 453.85 (397.21-513.34) | 558.46 (491.89-629.39) | 0.76 (0.73-0.8) |
| Azerbaijan | 33640.22 (29299.68-38154.81) | 57113.48 (50523.47-64954.82) | 0.7 | 916.5 (798.25-1039.5) | 1025.15 (906.86-1165.9) | 0.56 (0.46-0.66) |
| Bahamas | 1181.37 (1029.83-1343.34) | 1908.62 (1692.17-2158.04) | 0.62 | 829.83 (723.39-943.61) | 910.27 (807.04-1029.23) | 0.39 (0.35-0.43) |
| Bahrain | 4390.39 (3783.89-5028.26) | 16608.37 (14929.39-18413.09) | 2.78 | 1455.3 (1254.26-1666.73) | 1696.01 (1524.56-1880.31) | 0.52 (0.48-0.56) |
| Bangladesh | 341519.38 (297741.03-388180.93) | 730432.1 (639609.06-830849.84) | 1.14 | 683.34 (595.74-776.7) | 830.39 (727.14-944.55) | 0.67 (0.64-0.69) |
| Barbados | 1105.26 (960.95-1251.09) | 1235.4 (1095.5-1379.2) | 0.12 | 820.71 (713.55-929) | 884.14 (784.02-987.05) | 0.26 (0.22-0.3) |
| Belarus | 28897.16 (25388.48-32938.09) | 25643.21 (22634.08-28953.13) | -0.11 | 573.1 (503.51-653.24) | 602.7 (531.98-680.5) | 0.22 (0.2-0.24) |
| Belgium | 21814.35 (19106.92-24875.68) | 27230.26 (24074.1-30561.38) | 0.25 | 439.86 (385.27-501.59) | 545.24 (482.05-611.95) | 0.76 (0.75-0.78) |
| Belize | 769.01 (672.88-876.21) | 2615.53 (2319.78-2961.03) | 2.4 | 903.45 (790.51-1029.4) | 1103.14 (978.4-1248.86) | 0.7 (0.64-0.75) |
| Benin | 15909.53 (13721.89-18091.42) | 60370.9 (52789.12-68300.68) | 2.79 | 794.21 (685-903.13) | 965.63 (844.36-1092.47) | 0.66 (0.63-0.69) |
| Bermuda | 280.77 (246.69-320.32) | 240.23 (213.66-270.05) | -0.14 | 830.31 (729.52-947.25) | 894.89 (795.91-1005.99) | 0.32 (0.28-0.37) |
| Bhutan | 2195.49 (1901.12-2506.31) | 3871.28 (3365.96-4407.88) | 0.76 | 703.73 (609.37-803.36) | 884.72 (769.24-1007.35) | 0.84 (0.81-0.87) |
| Bolivia (Plurinational State of) | 21042.83 (18411.07-24089.27) | 51781.36 (45479.07-58351.21) | 1.46 | 703.67 (615.67-805.55) | 827.56 (726.84-932.56) | 0.58 (0.54-0.61) |
| Bosnia and Herzegovina | 19318.46 (16971.36-21735.77) | 13221.61 (11653.3-14822.46) | -0.32 | 806.96 (708.92-907.94) | 888 (782.67-995.52) | 0.5 (0.44-0.56) |
| Botswana | 4713.43 (4092.25-5380.87) | 12873.77 (11259.79-14595.65) | 1.73 | 782.07 (679-892.81) | 946.85 (828.15-1073.49) | 0.69 (0.67-0.71) |
| Brazil | 651619.48 (576390.02-737443.42) | 1132223.83 (1008389.01-1277338.41) | 0.74 | 850.36 (752.19-962.36) | 976.3 (869.52-1101.43) | 0.53 (0.5-0.56) |
| Brunei Darussalam | 948.4 (823.81-1081.99) | 1914.38 (1663.87-2163.17) | 1.02 | 647.55 (562.48-738.76) | 705.1 (612.83-796.73) | 0.36 (0.33-0.39) |
| Bulgaria | 26787.02 (23622.09-30368.98) | 18713.97 (16579.01-21170.8) | -0.3 | 644.41 (568.28-730.59) | 637.33 (564.62-721) | -0.02 (-0.05-0.01) |
| Burkina Faso | 22320.09 (19314.49-25444) | 72006.09 (62502.19-82630.18) | 2.23 | 582.21 (503.81-663.7) | 698.48 (606.29-801.54) | 0.6 (0.59-0.61) |
| Burundi | 16630.56 (14401.94-18932.55) | 46392.74 (40089.02-52968.58) | 1.79 | 685.7 (593.81-780.61) | 743.04 (642.07-848.36) | 0.28 (0.25-0.31) |
| Cabo Verde | 1253.15 (1082.45-1447.92) | 3086.54 (2694.71-3480.42) | 1.46 | 850.48 (734.63-982.66) | 983.39 (858.55-1108.88) | 0.57 (0.55-0.6) |
| Cambodia | 31684.11 (27405.04-36358.54) | 67558.02 (59117.11-76931.94) | 1.13 | 689.72 (596.57-791.48) | 749.34 (655.71-853.31) | 0.36 (0.3-0.42) |
| Cameroon | 39586.1 (34494.77-45313.08) | 155863.47 (134898.2-177522.58) | 2.94 | 874.25 (761.81-1000.73) | 1010.34 (874.44-1150.74) | 0.45 (0.43-0.46) |
| Canada | 54277.58 (47580.67-61991.88) | 69878.28 (60690.34-79378.12) | 0.29 | 368.11 (322.69-420.43) | 420.06 (364.83-477.17) | 0.46 (0.45-0.48) |
| Central African Republic | 7748.81 (6695.06-8976.36) | 17849.17 (15471.89-20604.33) | 1.3 | 624.08 (539.21-722.95) | 669.52 (580.35-772.87) | 0.22 (0.19-0.24) |
| Chad | 16934.18 (14680.28-19330.83) | 57333.42 (49896.36-65899.71) | 2.39 | 679.75 (589.28-775.95) | 773.02 (672.75-888.52) | 0.4 (0.37-0.42) |
| Chile | 34779.77 (30215.64-39607.14) | 57256.21 (49833.23-64836.88) | 0.65 | 489.17 (424.98-557.06) | 602.45 (524.34-682.21) | 0.72 (0.67-0.77) |
| China | 4982201.39 (4361506.5-5654689.7) | 6010047.66 (5304971.13-6794592.52) | 0.21 | 747.12 (654.04-847.97) | 906.08 (799.78-1024.36) | 0.67 (0.46-0.88) |
| Colombia | 145715.57 (128270.47-166406.57) | 260735.48 (231632.13-292733.7) | 0.79 | 861.43 (758.3-983.75) | 998.48 (887.03-1121.02) | 0.53 (0.51-0.56) |
| Comoros | 1921.94 (1662.56-2181.66) | 4032.64 (3548.64-4573.05) | 1.1 | 938.2 (811.59-1064.99) | 1029.5 (905.94-1167.46) | 0.27 (0.25-0.28) |
| Congo | 7121.96 (6178.48-8124.7) | 19917.95 (17366.95-22570.38) | 1.8 | 644.02 (558.7-734.69) | 706.79 (616.27-800.91) | 0.27 (0.25-0.3) |
| Cook Islands | 88.17 (77.22-99.38) | 86.27 (76.32-96.33) | -0.02 | 928.26 (812.98-1046.34) | 1069.86 (946.38-1194.63) | 0.48 (0.44-0.52) |
| Costa Rica | 13569.54 (11857.34-15465.53) | 24629.63 (21679.83-27673.04) | 0.82 | 880.41 (769.32-1003.43) | 983.73 (865.91-1105.29) | 0.44 (0.4-0.47) |
| C么te d'Ivoire | 39208.02 (34013.7-45119.95) | 110365.33 (95138.91-127300.02) | 1.81 | 704.68 (611.33-810.94) | 805.94 (694.75-929.61) | 0.42 (0.41-0.43) |
| Croatia | 14849.31 (12959.33-16773.38) | 12200.22 (10717.39-13723.66) | -0.18 | 608.24 (530.83-687.06) | 666.58 (585.57-749.82) | 0.39 (0.36-0.42) |
| Cuba | 49798.92 (43716.8-56179.48) | 46310.16 (41106.52-51937.43) | -0.07 | 809.39 (710.53-913.09) | 911.21 (808.82-1021.94) | 0.46 (0.38-0.54) |
| Cyprus | 1875.9 (1632.34-2143.21) | 3851.33 (3369.03-4392.22) | 1.05 | 464.57 (404.25-530.77) | 545.35 (477.05-621.94) | 0.55 (0.46-0.65) |
| Czechia | 27542.31 (24337.3-30914.07) | 26623.11 (23337.42-29900.58) | -0.03 | 528.95 (467.4-593.7) | 564.4 (494.75-633.88) | 0.17 (0.12-0.23) |
| Democratic People's Republic of Korea | 75190.18 (66270.36-85224.22) | 110693.13 (96529.14-126358.87) | 0.47 | 699.82 (616.8-793.22) | 800.39 (697.97-913.66) | 0.43 (0.4-0.46) |
| Democratic Republic of the Congo | 116315.79 (100327.71-132519.32) | 318285.36 (276877.17-360019.8) | 1.74 | 692.92 (597.67-789.44) | 734.8 (639.21-831.15) | 0.15 (0.11-0.2) |
| Denmark | 9929.69 (8697.41-11258.71) | 12163.01 (10679.68-13686.32) | 0.22 | 371.55 (325.44-421.28) | 472.98 (415.3-532.22) | 0.85 (0.82-0.87) |
| Djibouti | 1557.59 (1354.4-1765.24) | 5888.87 (5160.35-6647.14) | 2.78 | 748.83 (651.14-848.66) | 852.76 (747.26-962.56) | 0.47 (0.43-0.52) |
| Dominica | 290.9 (255.3-331.39) | 326.55 (290.76-367.05) | 0.12 | 822.55 (721.9-937.06) | 966.45 (860.55-1086.33) | 0.56 (0.54-0.58) |
| Dominican Republic | 25378.78 (22058.23-28843.22) | 47554.09 (42081.16-53416.5) | 0.87 | 698.03 (606.7-793.31) | 811.24 (717.87-911.25) | 0.61 (0.57-0.65) |
| Ecuador | 49499.23 (43326.8-55929.14) | 109556.08 (96856.16-122767.99) | 1.21 | 999.09 (874.51-1128.87) | 1166.39 (1031.18-1307.06) | 0.5 (0.48-0.52) |
| Egypt | 481484.79 (422635.49-539528.6) | 1104820.83 (995779.49-1223906.75) | 1.29 | 1793.97 (1574.7-2010.24) | 2067.16 (1863.14-2289.97) | 0.45 (0.41-0.49) |
| El Salvador | 21899.01 (19165.49-24975.88) | 35045.62 (30610.65-39711.76) | 0.6 | 875.24 (765.99-998.22) | 1058.58 (924.62-1199.52) | 0.62 (0.56-0.68) |
| Equatorial Guinea | 1119.69 (964.96-1286.57) | 7525.36 (6474.63-8612.62) | 5.72 | 619.29 (533.71-711.59) | 927.6 (798.08-1061.62) | 1.56 (1.48-1.64) |
| Eritrea | 10052.56 (8752.85-11523.9) | 24430.81 (21302.34-27893.99) | 1.43 | 652.14 (567.82-747.59) | 713.69 (622.3-814.86) | 0.26 (0.24-0.29) |
| Estonia | 3949.79 (3460.04-4467.24) | 3322.33 (2925.76-3764.56) | -0.16 | 520.16 (455.66-588.3) | 578.77 (509.69-655.81) | 0.44 (0.39-0.49) |
| Eswatini | 3582.72 (3119.93-4084.38) | 7098.73 (6190.49-8013.14) | 0.98 | 1006.01 (876.06-1146.87) | 1151.16 (1003.87-1299.44) | 0.38 (0.32-0.44) |
| Ethiopia | 144281.11 (125335.83-164704.08) | 422520.25 (365627.53-479588.14) | 1.93 | 664.56 (577.3-758.63) | 767.73 (664.35-871.42) | 0.47 (0.44-0.49) |
| Fiji | 4258.83 (3702.53-4832.82) | 6041.85 (5364.32-6748.12) | 0.42 | 1077.78 (937-1223.04) | 1291.22 (1146.42-1442.16) | 0.54 (0.49-0.59) |
| Finland | 8731.05 (7555.39-9905.19) | 10139.67 (8963.8-11459.41) | 0.16 | 338.12 (292.59-383.59) | 433.48 (383.21-489.9) | 0.87 (0.82-0.92) |
| France | 125502.12 (109496.33-142264.37) | 151641.23 (132717.39-169940.41) | 0.21 | 430.08 (375.23-487.52) | 536.05 (469.15-600.74) | 0.78 (0.76-0.8) |
| Gabon | 3088.85 (2692.49-3576.39) | 7988.69 (7026.92-9099.94) | 1.59 | 690.69 (602.06-799.7) | 861.33 (757.63-981.14) | 0.75 (0.72-0.77) |
| Gambia | 3532.2 (3040.82-4057.95) | 11072.47 (9640.65-12726.02) | 2.13 | 796.85 (686-915.46) | 933.54 (812.82-1072.95) | 0.52 (0.51-0.53) |
| Georgia | 22896.86 (19988.79-25906.04) | 14463.03 (12736.9-16397.02) | -0.37 | 853.78 (745.34-965.98) | 904.06 (796.16-1024.95) | 0.28 (0.23-0.33) |
| Germany | 162844.45 (141061.32-187304.61) | 183015.89 (161881.76-207070.95) | 0.12 | 408.28 (353.66-469.6) | 514.41 (455.01-582.02) | 0.9 (0.83-0.97) |
| Ghana | 49968.48 (43439.99-56692.99) | 166110.9 (146290.59-189637.94) | 2.32 | 730.81 (635.33-829.16) | 947.73 (834.64-1081.96) | 0.87 (0.84-0.9) |
| Greece | 22990.46 (20134.62-25850.42) | 22939 (20140.67-26041.16) | 0 | 455.23 (398.68-511.86) | 530.01 (465.35-601.69) | 0.44 (0.38-0.5) |
| Greenland | 138.81 (119.95-160.57) | 125.07 (109.4-143.22) | -0.1 | 414.79 (358.42-479.81) | 472.53 (413.32-541.12) | 0.6 (0.53-0.67) |
| Grenada | 289.38 (250.72-330.9) | 480.26 (420.93-541.29) | 0.66 | 739.5 (640.71-845.6) | 899.76 (788.62-1014.11) | 0.65 (0.63-0.68) |
| Guam | 702.12 (612.9-796.76) | 770.12 (688.26-862.48) | 0.1 | 907.97 (792.59-1030.36) | 1031.23 (921.61-1154.9) | 0.53 (0.48-0.58) |
| Guatemala | 31883.7 (28063.14-36186.9) | 91236.67 (80382.91-103585.54) | 1.86 | 901.42 (793.41-1023.09) | 1084.42 (955.41-1231.19) | 0.63 (0.58-0.67) |
| Guinea | 20958.25 (18247.1-23838.78) | 57919.96 (50370-66029.43) | 1.76 | 832.47 (724.78-946.88) | 944.63 (821.5-1076.89) | 0.39 (0.38-0.4) |
| Guinea-Bissau | 3372.37 (2945.27-3868.83) | 8621.79 (7521.33-9846.15) | 1.56 | 771.86 (674.11-885.49) | 862.04 (752.01-984.46) | 0.35 (0.33-0.36) |
| Guyana | 3032.66 (2648.09-3482.47) | 3599.38 (3187.1-4069.49) | 0.19 | 752.6 (657.16-864.23) | 900.19 (797.08-1017.76) | 0.63 (0.61-0.65) |
| Haiti | 21753.95 (19158.69-24960.4) | 55822.42 (48963.94-62797.76) | 1.57 | 741.3 (652.86-850.57) | 814.29 (714.24-916.04) | 0.37 (0.35-0.39) |
| Honduras | 20031.09 (17521.56-22650.45) | 62192.13 (54705.6-69798.22) | 2.1 | 977.96 (855.44-1105.85) | 1148.75 (1010.46-1289.24) | 0.55 (0.51-0.59) |
| Hungary | 31365.88 (27547.83-35657.96) | 28660.04 (25101.26-32417.42) | -0.09 | 616.29 (541.27-700.62) | 660.15 (578.17-746.69) | 0.17 (0.13-0.22) |
| Iceland | 615 (535.86-702.38) | 918.21 (807.33-1027.07) | 0.49 | 464.33 (404.58-530.3) | 557.37 (490.06-623.45) | 0.76 (0.71-0.81) |
| India | 2722098.8 (2379293.85-3091260.74) | 6285205.04 (5545990.71-7156079.69) | 1.31 | 647.5 (565.96-735.32) | 806.13 (711.32-917.83) | 0.68 (0.59-0.77) |
| Indonesia | 853320.83 (751168.03-968983.15) | 1608637.27 (1420228.8-1820951.45) | 0.89 | 902.79 (794.72-1025.16) | 1049.05 (926.18-1187.5) | 0.52 (0.5-0.54) |
| Iran (Islamic Republic of) | 382416.8 (336498.68-430535.73) | 852496.88 (766863.7-946918.9) | 1.23 | 1497.92 (1318.06-1686.4) | 1798.6 (1617.93-1997.82) | 0.69 (0.45-0.93) |
| Iraq | 128506.32 (111930.61-145190.12) | 374815.68 (332927.57-419674.71) | 1.92 | 1517.97 (1322.17-1715.05) | 1691.88 (1502.8-1894.37) | 0.38 (0.34-0.43) |
| Ireland | 8601.26 (7512.1-9761.11) | 13228.86 (11775.77-14882.95) | 0.54 | 482.82 (421.68-547.92) | 573.02 (510.08-644.66) | 0.5 (0.47-0.53) |
| Israel | 14739.78 (12845.43-16640.07) | 33203.86 (29294.16-37129.25) | 1.25 | 607.27 (529.22-685.56) | 738.95 (651.94-826.31) | 0.62 (0.57-0.67) |
| Italy | 188586.91 (165893.49-211212.67) | 183595.29 (162939.81-205631.18) | -0.03 | 656.61 (577.6-735.39) | 746.04 (662.11-835.59) | 0.52 (0.42-0.61) |
| Jamaica | 9606.56 (8386.67-10972.3) | 14680.93 (13015.88-16556.77) | 0.53 | 825.2 (720.41-942.51) | 959.15 (850.37-1081.7) | 0.59 (0.55-0.63) |
| Japan | 273058.03 (239780.73-306859.9) | 216633.02 (191221.44-243758.08) | -0.21 | 420.63 (369.37-472.7) | 427.46 (377.32-480.98) | -0.03 (-0.13-0.06) |
| Jordan | 27930.96 (24432.55-31700.16) | 126424.74 (112846.43-140996.93) | 3.53 | 1567.53 (1371.2-1779.07) | 1847.92 (1649.45-2060.92) | 0.58 (0.56-0.6) |
| Kazakhstan | 62350.52 (54665.81-71450.66) | 80650.81 (71203.15-90752.99) | 0.29 | 758.83 (665.3-869.58) | 862.05 (761.07-970.03) | 0.49 (0.43-0.55) |
| Kenya | 83698.9 (72858.08-95270.82) | 247055.01 (216483.67-280246.16) | 1.95 | 824.17 (717.43-938.12) | 944.96 (828.03-1071.92) | 0.44 (0.41-0.48) |
| Kiribati | 382.85 (331.73-433.61) | 735.28 (647.98-826.87) | 0.92 | 1043.98 (904.58-1182.38) | 1184.6 (1043.95-1332.16) | 0.36 (0.29-0.44) |
| Kuwait | 16485.12 (14435.83-18645.21) | 52072.53 (46165.89-58449.75) | 2.16 | 1608.98 (1408.97-1819.81) | 1709.8 (1515.85-1919.19) | 0.31 (0.25-0.38) |
| Kyrgyzstan | 18362.42 (16014.41-20864.61) | 31884.35 (27918.16-36090.98) | 0.74 | 876.59 (764.5-996.04) | 927.45 (812.08-1049.81) | 0.28 (0.24-0.33) |
| Lao People's Democratic Republic | 11344.49 (9942.87-13068.17) | 28168.09 (24828.37-32284.49) | 1.48 | 610.43 (535.01-703.18) | 703.79 (620.35-806.64) | 0.48 (0.47-0.5) |
| Latvia | 6778.69 (5962.35-7603.74) | 4502.25 (4002.52-5076.54) | -0.34 | 527.38 (463.87-591.57) | 571.17 (507.77-644.02) | 0.29 (0.26-0.33) |
| Lebanon | 20084.98 (17638.21-22563.46) | 48508.03 (43043.21-54484.42) | 1.42 | 1396.8 (1226.64-1569.16) | 1609.36 (1428.05-1807.64) | 0.58 (0.54-0.62) |
| Lesotho | 4861.01 (4205.52-5550.62) | 9232.23 (7997.04-10532.73) | 0.9 | 732.76 (633.95-836.71) | 919.25 (796.26-1048.74) | 0.78 (0.76-0.8) |
| Liberia | 9083.15 (7893.15-10417.16) | 26395.33 (22924.03-29871.56) | 1.91 | 838.75 (728.86-961.93) | 941.44 (817.63-1065.43) | 0.64 (0.56-0.72) |
| Libya | 31196.9 (27226.68-35244.49) | 71551.22 (64069.64-79783.58) | 1.29 | 1576.25 (1375.65-1780.76) | 1735.54 (1554.07-1935.22) | 0.3 (0.24-0.35) |
| Lithuania | 9504.11 (8282.65-10710.44) | 6498.93 (5711.33-7302.19) | -0.32 | 519.13 (452.41-585.02) | 562.53 (494.36-632.06) | 0.33 (0.3-0.36) |
| Luxembourg | 851.7 (744.09-967.41) | 1664.08 (1460.76-1878.84) | 0.95 | 428.25 (374.14-486.44) | 524.42 (460.35-592.1) | 0.78 (0.74-0.81) |
| Madagascar | 37408.18 (32701.93-42667.79) | 107559.55 (93791.67-122927.17) | 1.88 | 705.28 (616.55-804.44) | 761.8 (664.28-870.64) | 0.28 (0.26-0.31) |
| Malawi | 35500.15 (30977.98-41056.81) | 87863.04 (75520.28-100434.79) | 1.48 | 806.52 (703.78-932.76) | 907.58 (780.09-1037.44) | 0.41 (0.4-0.43) |
| Malaysia | 94519.37 (83287.23-106665.59) | 216244.78 (191089.34-245740.74) | 1.29 | 1049.21 (924.52-1184.03) | 1218.33 (1076.6-1384.51) | 0.54 (0.52-0.57) |
| Maldives | 828.13 (721.38-939.28) | 3072.41 (2686.87-3518.66) | 2.71 | 869.65 (757.55-986.38) | 907.55 (793.66-1039.36) | 0.26 (0.18-0.34) |
| Mali | 31148.23 (27095.41-35482.82) | 106920.66 (93559.42-121070.95) | 2.43 | 860.88 (748.87-980.68) | 1017.95 (890.74-1152.66) | 0.57 (0.56-0.57) |
| Malta | 876.52 (769.87-994) | 1105.23 (970.9-1249.86) | 0.26 | 457.49 (401.83-518.81) | 567.96 (498.93-642.29) | 0.73 (0.61-0.84) |
| Marshall Islands | 171.53 (148.47-194.18) | 303.05 (266.43-341.01) | 0.77 | 854.57 (739.67-967.39) | 993.88 (873.78-1118.37) | 0.44 (0.4-0.47) |
| Mauritania | 9383.14 (8160.39-10643.73) | 25328.58 (22137.68-28629.19) | 1.7 | 1029.12 (895.01-1167.38) | 1231.68 (1076.51-1392.18) | 0.57 (0.55-0.58) |
| Mauritius | 4567.53 (3979.65-5217.94) | 5755.18 (5044.5-6475.25) | 0.26 | 754.48 (657.37-861.92) | 899.53 (788.45-1012.07) | 0.63 (0.61-0.66) |
| Mexico | 413910.14 (363423.67-469632.43) | 734949.7 (653741.74-821153.65) | 0.78 | 973.14 (854.44-1104.15) | 1073.44 (954.83-1199.35) | 0.34 (0.32-0.35) |
| Micronesia (Federated States of) | 422.02 (366.14-476.68) | 574.3 (507.97-646.58) | 0.36 | 891.45 (773.42-1006.91) | 1066.38 (943.22-1200.59) | 0.52 (0.42-0.62) |
| Monaco | 62.46 (54.51-70.49) | 82.12 (72.79-92.16) | 0.31 | 453.01 (395.35-511.27) | 584.44 (518.04-655.91) | 0.83 (0.82-0.85) |
| Mongolia | 8072.35 (6992.84-9210.44) | 13473.71 (11848.75-15228.73) | 0.67 | 786.09 (680.97-896.92) | 798.85 (702.5-902.9) | 0.12 (0.06-0.17) |
| Montenegro | 2312.11 (2019.33-2598.99) | 2225.54 (1941.34-2518.81) | -0.04 | 724.93 (633.14-814.88) | 762.02 (664.71-862.43) | 0.25 (0.17-0.33) |
| Morocco | 164328.49 (145077.26-185641.58) | 313490.86 (279939.35-350388.4) | 0.91 | 1320.44 (1165.75-1491.7) | 1613.78 (1441.07-1803.72) | 0.7 (0.68-0.72) |
| Mozambique | 36839.09 (31993.85-42314.06) | 107449.94 (92939.08-124666.79) | 1.92 | 637.2 (553.39-731.9) | 753.4 (651.65-874.11) | 0.56 (0.53-0.58) |
| Myanmar | 172219.71 (149454.02-195210.59) | 281524.55 (247359.54-318605.08) | 0.63 | 843.51 (732.01-956.12) | 957.58 (841.37-1083.7) | 0.46 (0.4-0.52) |
| Namibia | 4657.87 (4086.86-5289.71) | 10148.75 (8938.98-11533.34) | 1.18 | 707.44 (620.71-803.41) | 786.31 (692.58-893.58) | 0.35 (0.33-0.36) |
| Nauru | 43.26 (38.03-49.57) | 58.43 (51.03-66.36) | 0.35 | 870.85 (765.5-997.9) | 1017.25 (888.4-1155.21) | 0.43 (0.35-0.52) |
| Nepal | 49826.75 (43264.87-56703.97) | 125132.45 (109185.59-143079.01) | 1.51 | 559.78 (486.06-637.05) | 751.74 (655.94-859.55) | 1 (0.96-1.05) |
| Netherlands | 34994.87 (30574.22-39713.67) | 39192.4 (34390.1-44076.96) | 0.12 | 431.72 (377.18-489.93) | 529.29 (464.43-595.26) | 0.72 (0.68-0.76) |
| New Zealand | 8299.21 (7281.46-9364.69) | 13597.87 (12070.36-15355.97) | 0.64 | 459.92 (403.52-518.96) | 558.95 (496.16-631.21) | 0.72 (0.69-0.75) |
| Nicaragua | 15419.93 (13360.7-17648.35) | 36946.71 (32231.13-41514.17) | 1.4 | 892.04 (772.92-1020.96) | 1028.53 (897.26-1155.68) | 0.49 (0.45-0.53) |
| Niger | 26855.06 (23161.46-30562.56) | 94102.54 (81845.13-106977.34) | 2.5 | 802.04 (691.73-912.77) | 907.27 (789.09-1031.4) | 0.41 (0.39-0.43) |
| Nigeria | 346441.81 (303687.78-392030.65) | 1074240.39 (941092.17-1210927.91) | 2.1 | 844.64 (740.4-955.79) | 995.9 (872.46-1122.62) | 0.52 (0.5-0.53) |
| Niue | 9.37 (8.24-10.58) | 8.58 (7.6-9.61) | -0.08 | 910.92 (801.27-1028.76) | 1096.95 (972.22-1229.71) | 0.56 (0.53-0.59) |
| North Macedonia | 7338.8 (6464.33-8279) | 8386.57 (7406.6-9528.32) | 0.14 | 710.7 (626.01-801.75) | 761.92 (672.89-865.65) | 0.35 (0.3-0.39) |
| Northern Mariana Islands | 301.72 (263.56-341.21) | 268.14 (237.5-301.77) | -0.11 | 1039.22 (907.79-1175.25) | 1130.51 (1001.34-1272.3) | 0.24 (0.18-0.29) |
| Norway | 8986.5 (7899.98-10141.78) | 12763.5 (11270.53-14276.62) | 0.42 | 414.78 (364.63-468.1) | 511.11 (451.32-571.7) | 0.79 (0.76-0.82) |
| Oman | 12812.68 (11130.94-14547.05) | 49388.35 (43591.44-55778.91) | 2.85 | 1287.73 (1118.71-1462.04) | 1649.58 (1455.96-1863.03) | 0.94 (0.81-1.07) |
| Pakistan | 375395.39 (328150.9-425512.04) | 1146573.83 (1006628.54-1297524.95) | 2.05 | 759.22 (663.67-860.58) | 939.89 (825.17-1063.63) | 0.71 (0.7-0.73) |
| Palau | 80.84 (70.39-91.25) | 95.17 (84.83-107.34) | 0.18 | 943.67 (821.67-1065.17) | 1049.81 (935.73-1184.03) | 0.38 (0.32-0.43) |
| Palestine | 13037.06 (11455.4-14863.51) | 45345.52 (39861.19-50930.6) | 2.48 | 1469.51 (1291.23-1675.38) | 1707.78 (1501.23-1918.12) | 0.52 (0.49-0.55) |
| Panama | 10300.27 (8925.62-11738.56) | 21482.13 (18700.92-24141.45) | 1.09 | 836.01 (724.43-952.74) | 992.51 (864.02-1115.38) | 0.56 (0.53-0.59) |
| Papua New Guinea | 15191.9 (13228.9-17330.79) | 45312.8 (39908.09-51453.01) | 1.98 | 764.28 (665.53-871.89) | 848.54 (747.33-963.53) | 0.36 (0.33-0.4) |
| Paraguay | 13310.96 (11644.07-14997.34) | 31195.81 (27298.83-35019.16) | 1.34 | 704.05 (615.88-793.24) | 808.44 (707.45-907.52) | 0.6 (0.55-0.65) |
| Peru | 74647.98 (65248.71-84738.53) | 156622.43 (139191.62-175255.78) | 1.1 | 698.26 (610.34-792.64) | 810.32 (720.14-906.72) | 0.53 (0.51-0.55) |
| Philippines | 225023.31 (196976.28-254455.63) | 481501.84 (424011.44-544385.93) | 1.14 | 723.36 (633.2-817.97) | 802.06 (706.29-906.81) | 0.33 (0.31-0.35) |
| Poland | 112758.22 (99355.72-126901.86) | 114590.22 (101619.02-128883.2) | 0.02 | 594.59 (523.92-669.18) | 639.37 (566.99-719.11) | 0.27 (0.24-0.31) |
| Portugal | 26004.88 (22759.78-29467.25) | 28324.1 (24949.6-31880.16) | 0.09 | 519.99 (455.1-589.23) | 618.43 (544.75-696.07) | 0.47 (0.44-0.51) |
| Puerto Rico | 16659.64 (14632.09-18822.31) | 15288.95 (13645.3-16964.35) | -0.08 | 903.07 (793.16-1020.3) | 1043.65 (931.45-1158.01) | 0.49 (0.46-0.53) |
| Qatar | 4289.49 (3749.29-4899.87) | 36367.86 (31961.65-41455.62) | 7.48 | 1471.74 (1286.4-1681.17) | 1658.3 (1457.38-1890.29) | 0.62 (0.54-0.71) |
| Republic of Korea | 135311.86 (118229.58-153876.64) | 128912.39 (111941.37-147542.38) | -0.05 | 523.26 (457.2-595.05) | 530.8 (460.92-607.51) | 0.6 (0.29-0.91) |
| Republic of Moldova | 12683.08 (11010.84-14376.17) | 11265.41 (9948.34-12772.54) | -0.11 | 577.27 (501.16-654.33) | 629.39 (555.8-713.59) | 0.41 (0.35-0.47) |
| Romania | 67978.72 (59785.81-77122.92) | 54266.56 (48040.85-60980.13) | -0.2 | 599.56 (527.3-680.21) | 651.4 (576.67-731.99) | 0.24 (0.23-0.26) |
| Russian Federation | 457559.5 (403448.9-520039.39) | 455757 (401814.56-514727.81) | 0 | 616.48 (543.58-700.66) | 675.82 (595.83-763.27) | 0.37 (0.33-0.4) |
| Rwanda | 18088.68 (15769.25-20891.16) | 43663.43 (38274.42-50191.46) | 1.41 | 573.17 (499.68-661.97) | 637.31 (558.65-732.59) | 0.41 (0.35-0.47) |
| Saint Kitts and Nevis | 168.66 (147.28-191.67) | 295.58 (259.01-334.74) | 0.75 | 847.71 (740.23-963.36) | 935.69 (819.92-1059.63) | 0.33 (0.3-0.35) |
| Saint Lucia | 473.94 (419.31-538.55) | 742.56 (656.95-834.27) | 0.57 | 711.75 (629.72-808.79) | 802.87 (710.3-902.02) | 0.42 (0.38-0.45) |
| Saint Vincent and the Grenadines | 411.15 (355.65-470.12) | 502.38 (440.41-564.99) | 0.22 | 769.8 (665.89-880.21) | 889.46 (779.75-1000.31) | 0.56 (0.53-0.59) |
| Samoa | 777.04 (671.91-882.98) | 1060.21 (933.7-1188.65) | 0.36 | 991.92 (857.72-1127.14) | 1057.13 (930.99-1185.2) | 0.16 (0.13-0.18) |
| San Marino | 59.77 (52.6-67.85) | 76.82 (68-86.43) | 0.29 | 480.08 (422.49-545.05) | 559.26 (495.05-629.2) | 0.49 (0.42-0.56) |
| Sao Tome and Principe | 428.38 (369.61-489.48) | 1090.97 (956.46-1233.02) | 1.55 | 856.52 (739.02-978.69) | 966.39 (847.23-1092.21) | 0.38 (0.37-0.39) |
| Saudi Arabia | 115989.59 (101162.14-131076.46) | 440667.48 (392508.3-491098.55) | 2.8 | 1446.32 (1261.43-1634.45) | 1741.78 (1551.42-1941.11) | 0.64 (0.57-0.71) |
| Senegal | 30873.36 (27023.74-35383.11) | 83513.38 (72864.45-94893.59) | 1.71 | 945.68 (827.77-1083.82) | 1079.52 (941.87-1226.63) | 0.42 (0.41-0.42) |
| Serbia | 39599.28 (34821.37-44715.48) | 38372.75 (34158.59-42825.25) | -0.03 | 831.2 (730.91-938.59) | 904.99 (805.6-1010) | 0.33 (0.3-0.36) |
| Seychelles | 323.56 (280.8-366.85) | 518.66 (458.71-586.34) | 0.6 | 873.47 (758.04-990.34) | 958.15 (847.41-1083.19) | 0.26 (0.24-0.28) |
| Sierra Leone | 14431.49 (12600.65-16427.78) | 37699.74 (32988.75-42712.3) | 1.61 | 759.46 (663.11-864.51) | 847.83 (741.88-960.55) | 0.41 (0.36-0.45) |
| Singapore | 12194.87 (10639.21-13961.58) | 19022 (16636.86-21969.19) | 0.56 | 641.33 (559.52-734.25) | 637.78 (557.81-736.59) | 0.02 (-0.06-0.09) |
| Slovakia | 14418.26 (12768.07-16328.67) | 14823.29 (13066.18-16694.03) | 0.03 | 539.66 (477.9-611.17) | 573.13 (505.2-645.47) | 0.22 (0.16-0.27) |
| Slovenia | 5658.79 (4985.68-6367.78) | 5181.24 (4555.48-5860.73) | -0.08 | 557.91 (491.55-627.81) | 590.62 (519.29-668.07) | 0.25 (0.23-0.28) |
| Solomon Islands | 1240.73 (1078.33-1423.65) | 3265.17 (2854.09-3736.67) | 1.63 | 818.82 (711.64-939.54) | 948.15 (828.78-1085.07) | 0.44 (0.39-0.49) |
| Somalia | 29978.2 (26311.09-34096.73) | 90882.81 (79217.38-102974.61) | 2.03 | 858.07 (753.11-975.95) | 912.22 (795.13-1033.59) | 0.23 (0.19-0.26) |
| South Africa | 178687.06 (157152.24-202371.88) | 336791.91 (298612.41-379439.58) | 0.88 | 947.12 (832.98-1072.66) | 1081.06 (958.51-1217.96) | 0.54 (0.5-0.58) |
| South Sudan | 22615.63 (19596.33-25879.52) | 42126.04 (36617.69-47798.71) | 0.86 | 837.62 (725.8-958.51) | 946.91 (823.09-1074.42) | 0.4 (0.38-0.41) |
| Spain | 99085.88 (86596.55-112411.19) | 115925.37 (102615.72-130809.95) | 0.17 | 510.19 (445.88-578.8) | 579.93 (513.35-654.39) | 0.26 (0.2-0.32) |
| Sri Lanka | 80811.7 (70805.6-91593.11) | 110037.56 (97270.88-123523.52) | 0.36 | 879.81 (770.87-997.19) | 998.07 (882.27-1120.39) | 0.4 (0.37-0.44) |
| Sudan | 78561.77 (68287.66-89226.18) | 248772.95 (216191.43-281107.69) | 2.17 | 862.15 (749.4-979.18) | 1112.66 (966.94-1257.28) | 0.85 (0.79-0.91) |
| Suriname | 1552.75 (1350.8-1763.43) | 2587.19 (2255.1-2910.17) | 0.67 | 785.35 (683.21-891.91) | 900.83 (785.2-1013.29) | 0.51 (0.48-0.54) |
| Sweden | 17949.02 (15880.89-20300.96) | 24480.93 (21835.35-27692.02) | 0.36 | 427.14 (377.92-483.11) | 540.81 (482.36-611.74) | 0.85 (0.81-0.89) |
| Switzerland | 15328.25 (13404.57-17281.98) | 20119.69 (17761.84-22637.48) | 0.31 | 422.51 (369.48-476.36) | 502.33 (443.46-565.19) | 0.68 (0.65-0.71) |
| Syrian Arab Republic | 83808.37 (73080.13-95690.48) | 122255.4 (107992.12-137705.63) | 0.46 | 1495.7 (1304.24-1707.76) | 1722.11 (1521.19-1939.74) | 0.37 (0.3-0.43) |
| Taiwan (Province of China) | 91801.44 (79191.61-104643.1) | 106331.89 (93529.7-121406.67) | 0.16 | 815.46 (703.45-929.53) | 935.01 (822.43-1067.56) | 0.67 (0.56-0.77) |
| Tajikistan | 19917.39 (17282.49-22732.73) | 45250.75 (39679.45-51538.38) | 1.27 | 826.31 (717-943.11) | 880.25 (771.87-1002.56) | 0.3 (0.24-0.35) |
| Thailand | 239920.16 (210407.97-272617.44) | 279816.72 (244338.81-317517.11) | 0.17 | 757.87 (664.65-861.16) | 876.68 (765.53-994.8) | 0.45 (0.43-0.47) |
| Timor-Leste | 3025.32 (2662.6-3444.47) | 6134.95 (5375.37-6958.73) | 1.03 | 790.25 (695.51-899.74) | 889.63 (779.48-1009.08) | 0.39 (0.36-0.41) |
| Togo | 12461.04 (10742.52-14244.89) | 36573.7 (31785.76-41344.19) | 1.94 | 774.88 (668.01-885.8) | 879.92 (764.72-994.69) | 0.38 (0.37-0.4) |
| Tokelau | 5.99 (5.24-6.84) | 6.81 (6.01-7.66) | 0.14 | 857.72 (749.73-978.46) | 1036.83 (915.56-1165.3) | 0.67 (0.64-0.7) |
| Tonga | 451.91 (396.66-510.48) | 576.41 (509.46-650.35) | 0.28 | 1020.64 (895.84-1152.92) | 1171.57 (1035.5-1321.87) | 0.3 (0.22-0.38) |
| Trinidad and Tobago | 4906.7 (4292.07-5548.7) | 6075.29 (5319.57-6886.07) | 0.24 | 793.17 (693.81-896.95) | 880.4 (770.88-997.89) | 0.45 (0.4-0.5) |
| Tunisia | 53184.86 (46532.74-59675.42) | 89509.18 (79969.69-99959.15) | 0.68 | 1298.25 (1135.87-1456.68) | 1480.87 (1323.05-1653.76) | 0.48 (0.46-0.51) |
| Turkey | 378393.16 (335262.7-428633.87) | 662816.98 (590358.77-730636.09) | 0.75 | 1305.53 (1156.72-1478.87) | 1508.63 (1343.71-1662.99) | 0.48 (0.46-0.5) |
| Turkmenistan | 15026.35 (13031.81-17094.03) | 27276.26 (23871.23-30803.36) | 0.82 | 854.64 (741.19-972.24) | 1019.01 (891.8-1150.77) | 0.67 (0.61-0.73) |
| Tuvalu | 36.25 (31.85-41.48) | 63.74 (56.06-71.99) | 0.76 | 802.62 (705.16-918.4) | 1020 (897-1152) | 0.74 (0.7-0.78) |
| Uganda | 45462.06 (39250.88-51820.37) | 142338.73 (123822.91-163198.6) | 2.13 | 611.86 (528.26-697.43) | 708.11 (615.99-811.88) | 0.55 (0.52-0.58) |
| Ukraine | 154790.73 (135414.2-174620.42) | 128199.07 (112798.17-145589.14) | -0.17 | 620.36 (542.71-699.84) | 633.83 (557.68-719.8) | 0.11 (0.07-0.15) |
| United Arab Emirates | 16422.77 (14407.47-18708.03) | 90968.93 (78467.19-106161.49) | 4.54 | 1393.29 (1222.32-1587.17) | 1325.28 (1143.15-1546.61) | 0.02 (-0.24-0.29) |
| United Kingdom | 130899.17 (115511.96-147513.09) | 177034.75 (157998.06-197253.14) | 0.35 | 459.92 (405.86-518.3) | 583.42 (520.68-650.05) | 0.87 (0.83-0.91) |
| United Republic of Tanzania | 90980.84 (78242.33-103545.76) | 255640.48 (220096.02-292122.42) | 1.81 | 804.62 (691.96-915.74) | 906.59 (780.54-1035.97) | 0.39 (0.36-0.43) |
| United States of America | 603779.74 (528121.28-686276.34) | 845995.47 (743952.61-949949.19) | 0.4 | 449.76 (393.4-511.21) | 556.58 (489.44-624.97) | 0.85 (0.79-0.9) |
| United States Virgin Islands | 409.72 (358.38-459.68) | 298.01 (265.34-333.22) | -0.27 | 746.71 (653.14-837.76) | 881.6 (784.96-985.77) | 0.61 (0.58-0.65) |
| Uruguay | 6655.14 (5791.6-7546.94) | 9109.23 (7986.62-10365.91) | 0.37 | 447.92 (389.8-507.94) | 555.06 (486.66-631.63) | 0.72 (0.69-0.75) |
| Uzbekistan | 85366.15 (73948.48-98174.81) | 170684.79 (150179.61-192481.95) | 1 | 872.85 (756.11-1003.82) | 957.08 (842.1-1079.3) | 0.47 (0.41-0.54) |
| Vanuatu | 690.88 (606.35-779.05) | 1793.01 (1577.37-2003.63) | 1.6 | 983.78 (863.42-1109.33) | 1149.11 (1010.92-1284.1) | 0.52 (0.51-0.53) |
| Venezuela (Bolivarian Republic of) | 85194.16 (74340.12-96281.26) | 123471.63 (108458.83-138900.84) | 0.45 | 888.37 (775.19-1003.98) | 939.04 (824.86-1056.38) | 0.31 (0.25-0.37) |
| Viet Nam | 231856.82 (199560.67-266077.82) | 388826.9 (342654.97-441475.07) | 0.68 | 707.2 (608.69-811.57) | 746.62 (657.96-847.71) | 0.36 (0.3-0.42) |
| Yemen | 62498.84 (54839.57-70558.9) | 224609.66 (196478.37-251902.35) | 2.59 | 1148.51 (1007.76-1296.63) | 1342.99 (1174.79-1506.18) | 0.53 (0.51-0.55) |
| Zambia | 27146.24 (23481.53-31120.07) | 82643.18 (71880.38-93977.77) | 2.04 | 768.38 (664.65-880.86) | 855.39 (743.99-972.71) | 0.31 (0.27-0.34) |
| Zimbabwe | 37333.29 (32588.46-42635.58) | 66721.03 (58009.63-75240.81) | 0.79 | 809.84 (706.91-924.86) | 861.42 (748.95-971.42) | 0.18 (0.13-0.22) |
