# Supplementary material for: Global, regional and national burden of Metabolic dysfunction-associated steatotic liver disease in adolescents and adults aged 15–49 years from 1990 to 2021: results from the 2021 Global Burden of Disease study
Source: Front Med (Lausanne). 2025 Jun 25;12:1568211. doi: 10.3389/fmed.2025.1568211 (PMC12237898; doi:10.3389/fmed.2025.1568211)
Supplement: Supplementary file 1 [file Supplementary_file_1.ZIP › Supplementary Table 6 .docx]

**Supplementary Table 6** The DALYs of MASLD cases and rates in the adolescents and adults aged 15-49 years in 1990 and 2021 across 204 countries, and the trends from 1990 to 2021

| **location** | **DALYs cases** | | | **DALYs rates** | | |
| --- | --- | --- | --- | --- | --- | --- |
|  | **1990**  **(95%UI)** | **2021**  **(95%UI)** | **percentage**  **Change**  **(100%)** | **1990**  **Per 100,000**  **(95%UI)** | **2021**  **Per 100,000**  **(95%UI)** | **EAPC**  **(95% CI)** |
| Afghanistan | 483.97 (228.96-903.28) | 1274.8 (524.5-2465.5) | 1.63 | 12.1 (5.73-22.59) | 8.63 (3.55-16.68) | -0.53 (-0.96--0.11) |
| Albania | 90.59 (53.74-144.9) | 90.43 (50.14-154.69) | 0 | 5.29 (3.14-8.47) | 7.18 (3.98-12.28) | 1.33 (1.07-1.59) |
| Algeria | 508.28 (291.72-808.63) | 1613.15 (913.4-2717.66) | 2.17 | 4.32 (2.48-6.87) | 7.12 (4.03-12) | 1.85 (1.65-2.05) |
| American Samoa | 6.47 (3.7-10.33) | 9.22 (5.24-14.89) | 0.43 | 26.56 (15.2-42.45) | 38.47 (21.85-62.08) | 0.9 (0.75-1.06) |
| Andorra | 8.19 (4.11-14.84) | 11.27 (5.47-19.95) | 0.38 | 25.27 (12.69-45.78) | 27.34 (13.27-48.38) | 0.75 (0.48-1.02) |
| Angola | 907.46 (518.51-1556.48) | 2668.26 (1431.97-4378.37) | 1.94 | 19.5 (11.14-33.44) | 18.2 (9.77-29.86) | 0.04 (-0.15-0.23) |
| Antigua and Barbuda | 6.26 (3.82-10.04) | 9.43 (5.65-14.81) | 0.51 | 19.92 (12.15-31.94) | 19.9 (11.92-31.25) | 0.94 (0.47-1.41) |
| Argentina | 2940.19 (1712.15-4667.61) | 2969.74 (1740.87-4813.44) | 0.01 | 18.5 (10.77-29.37) | 12.62 (7.4-20.45) | -0.82 (-1.14--0.5) |
| Armenia | 191.68 (120.66-293.11) | 271.24 (154.12-416.37) | 0.42 | 11.23 (7.07-17.17) | 18.63 (10.58-28.59) | 2.13 (1.35-2.92) |
| Australia | 1041.13 (632.02-1558.15) | 1762.97 (1124.29-2593.31) | 0.69 | 11.59 (7.03-17.34) | 14.68 (9.36-21.6) | 1.05 (0.73-1.38) |
| Austria | 1714.16 (992.61-2686.86) | 853.85 (516.07-1316.65) | -0.5 | 42.58 (24.66-66.74) | 21.16 (12.79-32.64) | -2.1 (-2.42--1.79) |
| Azerbaijan | 823.4 (517.99-1272.87) | 1683.04 (959.9-2767.4) | 1.04 | 22.43 (14.11-34.68) | 30.21 (17.23-49.67) | 0.72 (0.41-1.02) |
| Bahamas | 66.14 (40.8-101.84) | 121.74 (71.98-199.16) | 0.84 | 46.46 (28.66-71.54) | 58.06 (34.33-94.98) | 0.82 (0.58-1.06) |
| Bahrain | 17.87 (10.53-28.51) | 98.17 (56.27-159.32) | 4.49 | 5.92 (3.49-9.45) | 10.03 (5.75-16.27) | 1.07 (0.72-1.42) |
| Bangladesh | 6346.01 (4026.55-9870.26) | 8890.4 (4959.47-14733.88) | 0.4 | 12.7 (8.06-19.75) | 10.11 (5.64-16.75) | -0.59 (-0.76--0.41) |
| Barbados | 42.27 (25.28-66.39) | 34.01 (18.56-55.45) | -0.2 | 31.39 (18.77-49.3) | 24.34 (13.28-39.69) | -0.99 (-1.27--0.71) |
| Belarus | 349.96 (206.88-552.28) | 1997.66 (1072.87-3311.81) | 4.71 | 6.94 (4.1-10.95) | 46.95 (25.22-77.84) | 6.13 (4.7-7.58) |
| Belgium | 1630.33 (903.9-2619.64) | 1387.95 (826.62-2032.66) | -0.15 | 32.87 (18.23-52.82) | 27.79 (16.55-40.7) | -1.35 (-1.82--0.88) |
| Belize | 18.37 (11.35-28.2) | 135.33 (81.33-212.36) | 6.37 | 21.58 (13.34-33.14) | 57.08 (34.3-89.57) | 2.91 (2.47-3.36) |
| Benin | 289.26 (162.1-486.01) | 1078.32 (563.24-1874.39) | 2.73 | 14.44 (8.09-24.26) | 17.25 (9.01-29.98) | 0.65 (0.53-0.77) |
| Bermuda | 12.15 (7.5-18.01) | 8.3 (4.97-13.25) | -0.32 | 35.92 (22.19-53.25) | 30.93 (18.5-49.34) | -0.39 (-1-0.21) |
| Bhutan | 45.26 (22.47-81.06) | 93.54 (48.08-164.71) | 1.07 | 14.51 (7.2-25.98) | 21.38 (10.99-37.64) | 1.04 (0.95-1.13) |
| Bolivia (Plurinational State of) | 1530.45 (622.07-2653.89) | 3328.54 (1723.53-5654.29) | 1.17 | 51.18 (20.8-88.75) | 53.2 (27.55-90.37) | -0.33 (-0.57--0.08) |
| Bosnia and Herzegovina | 228.89 (132.73-368.18) | 183.27 (97.59-317.22) | -0.2 | 9.56 (5.54-15.38) | 12.31 (6.55-21.31) | 0.63 (0.37-0.9) |
| Botswana | 87.29 (39.4-178.09) | 237.25 (131.59-417.62) | 1.72 | 14.48 (6.54-29.55) | 17.45 (9.68-30.72) | 0.51 (0.27-0.75) |
| Brazil | 16241.72 (10605.91-24252.42) | 25326.95 (15936.42-37239.34) | 0.56 | 21.2 (13.84-31.65) | 21.84 (13.74-32.11) | -0.02 (-0.22-0.17) |
| Brunei Darussalam | 6.49 (3.46-10.65) | 14.88 (8.03-26.01) | 1.29 | 4.43 (2.37-7.27) | 5.48 (2.96-9.58) | 0.84 (0.58-1.11) |
| Bulgaria | 768.47 (446.98-1209.47) | 1085.16 (581.62-1858.09) | 0.41 | 18.49 (10.75-29.1) | 36.96 (19.81-63.28) | 1.9 (1.41-2.39) |
| Burkina Faso | 325.69 (193.44-537.88) | 1052.44 (560.27-1684.85) | 2.23 | 8.5 (5.05-14.03) | 10.21 (5.43-16.34) | 0.97 (0.83-1.11) |
| Burundi | 367.9 (203.51-645.5) | 695.12 (385.66-1110.56) | 0.89 | 15.17 (8.39-26.61) | 11.13 (6.18-17.79) | -1.69 (-1.94--1.43) |
| Cabo Verde | 19.14 (11.59-31.04) | 51.99 (29.22-85.94) | 1.72 | 12.99 (7.87-21.07) | 16.56 (9.31-27.38) | 0.67 (0.47-0.87) |
| Cambodia | 1063.39 (609.67-1840.31) | 1806.19 (991.4-3175.81) | 0.7 | 23.15 (13.27-40.06) | 20.03 (11-35.23) | -0.73 (-0.82--0.63) |
| Cameroon | 869.85 (482.5-1403.14) | 3577.26 (1867.55-6213.43) | 3.11 | 19.21 (10.66-30.99) | 23.19 (12.11-40.28) | 0.75 (0.64-0.87) |
| Canada | 2066.55 (1232.32-3268.37) | 4406.15 (2660.36-6441.9) | 1.13 | 14.02 (8.36-22.17) | 26.49 (15.99-38.72) | 2.17 (1.92-2.43) |
| Central African Republic | 253.33 (135.48-422.86) | 614.94 (336.79-1087) | 1.43 | 20.4 (10.91-34.06) | 23.07 (12.63-40.77) | 0.41 (0.32-0.5) |
| Chad | 281.37 (158.91-478.74) | 1014.05 (560.5-1785.05) | 2.6 | 11.29 (6.38-19.22) | 13.67 (7.56-24.07) | 0.81 (0.7-0.91) |
| Chile | 3243.31 (1980.88-5191.76) | 3039.8 (1816.22-4726.07) | -0.06 | 45.62 (27.86-73.02) | 31.98 (19.11-49.73) | -0.16 (-0.5-0.18) |
| China | 41305 (25714.01-62653.71) | 29976.43 (16882.64-46825.4) | -0.27 | 6.19 (3.86-9.4) | 4.52 (2.55-7.06) | -0.96 (-1.1--0.81) |
| Colombia | 1592.12 (973.4-2487.3) | 2591.34 (1462.95-4110.29) | 0.63 | 9.41 (5.75-14.7) | 9.92 (5.6-15.74) | -0.09 (-0.22-0.03) |
| Comoros | 31 (15.3-51.84) | 72.81 (40.54-119.16) | 1.35 | 15.13 (7.47-25.31) | 18.59 (10.35-30.42) | 0.3 (-0.1-0.71) |
| Congo | 264.14 (141.46-450.63) | 833.95 (433.73-1526.43) | 2.16 | 23.89 (12.79-40.75) | 29.59 (15.39-54.17) | 0.75 (0.59-0.9) |
| Cook Islands | 0.32 (0.17-0.54) | 0.39 (0.2-0.68) | 0.22 | 3.33 (1.78-5.69) | 4.86 (2.43-8.43) | 1.44 (1.23-1.66) |
| Costa Rica | 389.11 (238.95-590.1) | 1004.91 (590.37-1584.38) | 1.58 | 25.25 (15.5-38.29) | 40.14 (23.58-63.28) | 0.67 (0.39-0.96) |
| C么te d'Ivoire | 781.19 (452.11-1272.61) | 2377.32 (1310.67-4122.84) | 2.04 | 14.04 (8.13-22.87) | 17.36 (9.57-30.11) | 0.88 (0.77-0.99) |
| Croatia | 578.66 (329.08-929.63) | 264.12 (146.09-438.51) | -0.54 | 23.7 (13.48-38.08) | 14.43 (7.98-23.96) | -1.91 (-2.23--1.59) |
| Cuba | 1006.51 (627.42-1555.85) | 2170.46 (1231.69-3499.44) | 1.16 | 16.36 (10.2-25.29) | 42.71 (24.24-68.86) | 3.52 (3.17-3.88) |
| Cyprus | 62.74 (33.14-104.23) | 84.38 (47.4-141.05) | 0.34 | 15.54 (8.21-25.81) | 11.95 (6.71-19.97) | -1.37 (-1.65--1.09) |
| Czechia | 1002.09 (577.41-1616) | 1172.89 (660.52-1919.5) | 0.17 | 19.24 (11.09-31.04) | 24.86 (14-40.69) | 0.6 (0.37-0.83) |
| Democratic People's Republic of Korea | 649.82 (350.33-1199.44) | 780.22 (333.5-1485.06) | 0.2 | 6.05 (3.26-11.16) | 5.64 (2.41-10.74) | -0.02 (-0.07-0.04) |
| Democratic Republic of the Congo | 2354.33 (1338.8-3839.39) | 5971.9 (3232.04-9523.29) | 1.54 | 14.03 (7.98-22.87) | 13.79 (7.46-21.99) | -0.08 (-0.19-0.02) |
| Denmark | 782.12 (442.68-1246.52) | 368.08 (213.07-571.08) | -0.53 | 29.27 (16.56-46.64) | 14.31 (8.29-22.21) | -3.25 (-4.03--2.47) |
| Djibouti | 20.32 (10.74-36.77) | 103.89 (54.16-184.41) | 4.11 | 9.77 (5.16-17.68) | 15.04 (7.84-26.7) | 1.37 (1.19-1.54) |
| Dominica | 7.38 (4.42-11.5) | 11.73 (6.67-19.47) | 0.59 | 20.88 (12.49-32.53) | 34.73 (19.74-57.62) | 1.86 (1.68-2.04) |
| Dominican Republic | 1281.02 (794.11-2059.89) | 3137.44 (1550.63-5447.63) | 1.45 | 35.23 (21.84-56.66) | 53.52 (26.45-92.93) | 1.87 (1.5-2.24) |
| Ecuador | 3057.76 (1978.56-4547.94) | 4683.4 (2989.75-7122.6) | 0.53 | 61.72 (39.94-91.8) | 49.86 (31.83-75.83) | -0.81 (-1.14--0.48) |
| Egypt | 5200.86 (3337.73-8143.2) | 9398.87 (5501.26-15586.06) | 0.81 | 19.38 (12.44-30.34) | 17.59 (10.29-29.16) | -0.46 (-0.65--0.27) |
| El Salvador | 1110.15 (669.48-1738.52) | 2325.21 (1263.89-3871.26) | 1.09 | 44.37 (26.76-69.48) | 70.23 (38.18-116.93) | 1.51 (1.3-1.72) |
| Equatorial Guinea | 32.44 (16.5-54.53) | 168.96 (80.31-316.16) | 4.21 | 17.94 (9.13-30.16) | 20.83 (9.9-38.97) | 0.7 (0.29-1.11) |
| Eritrea | 225.58 (125.63-374.89) | 507.5 (276.79-922.71) | 1.25 | 14.63 (8.15-24.32) | 14.83 (8.09-26.95) | -0.02 (-0.1-0.07) |
| Estonia | 115.92 (66.42-188.48) | 265.7 (147-428.43) | 1.29 | 15.27 (8.75-24.82) | 46.29 (25.61-74.64) | 3.04 (2.23-3.86) |
| Eswatini | 90.56 (47.77-166.6) | 286.09 (148.89-537.78) | 2.16 | 25.43 (13.41-46.78) | 46.39 (24.15-87.21) | 2 (1.33-2.68) |
| Ethiopia | 2906.9 (1366.31-4957.63) | 5331.67 (3362.45-8232.34) | 0.83 | 13.39 (6.29-22.83) | 9.69 (6.11-14.96) | -1.49 (-1.69--1.29) |
| Fiji | 43.13 (26.78-69.09) | 64.96 (34.79-109.11) | 0.51 | 10.91 (6.78-17.48) | 13.88 (7.44-23.32) | 0.56 (0.46-0.67) |
| Finland | 835.65 (492.66-1346.3) | 619.16 (371.22-943.4) | -0.26 | 32.36 (19.08-52.14) | 26.47 (15.87-40.33) | -0.23 (-0.96-0.5) |
| France | 8830.9 (5013.38-14307.24) | 4236.73 (2463.24-6845.19) | -0.52 | 30.26 (17.18-49.03) | 14.98 (8.71-24.2) | -2.53 (-2.94--2.12) |
| Gabon | 95.92 (54.72-156.81) | 323.95 (171.47-600.31) | 2.38 | 21.45 (12.24-35.06) | 34.93 (18.49-64.72) | 1.6 (1.35-1.84) |
| Gambia | 45.75 (24.03-75.05) | 174.88 (89.28-292.37) | 2.82 | 10.32 (5.42-16.93) | 14.74 (7.53-24.65) | 1.19 (0.89-1.49) |
| Georgia | 705.35 (436.13-1097.06) | 587.12 (332.82-958.1) | -0.17 | 26.3 (16.26-40.91) | 36.7 (20.8-59.89) | 1.92 (1.36-2.47) |
| Germany | 19430.61 (11323.5-30504.36) | 10158.44 (5882.7-15212.47) | -0.48 | 48.72 (28.39-76.48) | 28.55 (16.53-42.76) | -2.13 (-2.56--1.71) |
| Ghana | 1017.86 (621.31-1643.19) | 3973.19 (2188.36-6543.77) | 2.9 | 14.89 (9.09-24.03) | 22.67 (12.49-37.33) | 1.45 (1.32-1.59) |
| Greece | 600.57 (368.48-936.56) | 703.5 (417.3-1068.46) | 0.17 | 11.89 (7.3-18.54) | 16.25 (9.64-24.69) | 1.39 (0.94-1.83) |
| Greenland | 10.58 (6-17.3) | 8.78 (4.95-15.42) | -0.17 | 31.62 (17.94-51.71) | 33.17 (18.71-58.26) | 0.61 (0.13-1.08) |
| Grenada | 9.85 (6.02-15.4) | 17.36 (9.72-26.73) | 0.76 | 25.18 (15.39-39.36) | 32.52 (18.22-50.08) | 0.83 (0.58-1.07) |
| Guam | 17.36 (10.71-27.94) | 28.76 (17.37-47.23) | 0.66 | 22.45 (13.85-36.13) | 38.51 (23.27-63.25) | 2.14 (1.97-2.3) |
| Guatemala | 2497.9 (1527.75-3892.76) | 7115.94 (4092.46-11484.2) | 1.85 | 70.62 (43.19-110.06) | 84.58 (48.64-136.5) | 0.21 (-0.12-0.54) |
| Guinea | 360.66 (204.51-581.11) | 911.08 (542.82-1558.41) | 1.53 | 14.33 (8.12-23.08) | 14.86 (8.85-25.42) | 0.26 (0.2-0.33) |
| Guinea-Bissau | 92.74 (51.57-159.15) | 220.14 (111.64-376.1) | 1.37 | 21.23 (11.8-36.43) | 22.01 (11.16-37.6) | 0.21 (0.06-0.36) |
| Guyana | 238.22 (142.64-375.95) | 357.18 (189.49-586.74) | 0.5 | 59.12 (35.4-93.3) | 89.33 (47.39-146.74) | 1.84 (1.51-2.17) |
| Haiti | 1150.68 (565.13-1876.25) | 2093.85 (1095.44-3695.57) | 0.82 | 39.21 (19.26-63.94) | 30.54 (15.98-53.91) | -0.77 (-0.96--0.57) |
| Honduras | 1029.34 (604.22-1644.26) | 2558.76 (1134.86-4742.04) | 1.49 | 50.25 (29.5-80.28) | 47.26 (20.96-87.59) | -0.63 (-0.84--0.42) |
| Hungary | 3466.52 (1958.31-5435.38) | 1112.15 (632.33-1888.56) | -0.68 | 68.11 (38.48-106.8) | 25.62 (14.56-43.5) | -5.55 (-6.28--4.81) |
| Iceland | 7.63 (4.54-12.4) | 10.82 (6.36-17.42) | 0.42 | 5.76 (3.43-9.36) | 6.57 (3.86-10.58) | -0.16 (-0.41-0.1) |
| India | 58941.67 (37468.11-91121.23) | 114739.54 (66420.83-182993.64) | 0.95 | 14.02 (8.91-21.67) | 14.72 (8.52-23.47) | 0.15 (-0.02-0.31) |
| Indonesia | 17339.38 (10828.09-27774.46) | 34354.02 (21684.89-54915.43) | 0.98 | 18.34 (11.46-29.38) | 22.4 (14.14-35.81) | 0.86 (0.74-0.98) |
| Iran (Islamic Republic of) | 2479.45 (1571.32-3844.59) | 6107.81 (3881.58-9140.12) | 1.46 | 9.71 (6.15-15.06) | 12.89 (8.19-19.28) | 0.99 (0.86-1.13) |
| Iraq | 643.13 (371.88-1042.2) | 1501.24 (814.62-2577.37) | 1.33 | 7.6 (4.39-12.31) | 6.78 (3.68-11.63) | -0.16 (-0.32--0.01) |
| Ireland | 179.54 (108.66-285.41) | 388.7 (226.54-585.5) | 1.16 | 10.08 (6.1-16.02) | 16.84 (9.81-25.36) | 1.86 (1.15-2.57) |
| Israel | 280.77 (167-433.43) | 448.82 (277.24-681.33) | 0.6 | 11.57 (6.88-17.86) | 9.99 (6.17-15.16) | -0.69 (-1.18--0.19) |
| Italy | 8929.22 (5681.8-13202.36) | 3058.51 (1913.58-4558.76) | -0.66 | 31.09 (19.78-45.97) | 12.43 (7.78-18.52) | -2.8 (-2.98--2.62) |
| Jamaica | 100.68 (62.06-155.01) | 198.85 (111.34-325.73) | 0.98 | 8.65 (5.33-13.32) | 12.99 (7.27-21.28) | 0.86 (0.5-1.22) |
| Japan | 5317.97 (3178.69-8423.26) | 2764.01 (1612.08-4481.92) | -0.48 | 8.19 (4.9-12.98) | 5.45 (3.18-8.84) | -1.31 (-1.51--1.12) |
| Jordan | 82.49 (46.75-135.77) | 335.11 (186.71-541.76) | 3.06 | 4.63 (2.62-7.62) | 4.9 (2.73-7.92) | -0.05 (-0.32-0.22) |
| Kazakhstan | 942.09 (568.91-1503.25) | 6170.21 (3471.94-10211.82) | 5.55 | 11.47 (6.92-18.3) | 65.95 (37.11-109.15) | 5.74 (4.54-6.95) |
| Kenya | 1311.39 (681.76-2436.55) | 5983.95 (3452.05-10524.88) | 3.56 | 12.91 (6.71-23.99) | 22.89 (13.2-40.26) | 2.23 (2.07-2.4) |
| Kiribati | 11.16 (6.29-18.65) | 20.98 (11.93-37.36) | 0.88 | 30.44 (17.15-50.86) | 33.79 (19.22-60.2) | -0.08 (-0.39-0.22) |
| Kuwait | 45.32 (26.62-71.29) | 152.29 (83.42-242.1) | 2.36 | 4.42 (2.6-6.96) | 5 (2.74-7.95) | 0.63 (-0.3-1.57) |
| Kyrgyzstan | 547.99 (333.82-858.1) | 1708.97 (957.91-2840.59) | 2.12 | 26.16 (15.94-40.96) | 49.71 (27.86-82.63) | 1.62 (0.87-2.37) |
| Lao People's Democratic Republic | 213.9 (124.12-371.68) | 359.16 (191.73-601.58) | 0.68 | 11.51 (6.68-20) | 8.97 (4.79-15.03) | -1.04 (-1.14--0.94) |
| Latvia | 185.54 (108.66-297.85) | 437.35 (237.03-692.31) | 1.36 | 14.44 (8.45-23.17) | 55.48 (30.07-87.83) | 3.71 (2.91-4.52) |
| Lebanon | 82.82 (39.24-159.12) | 144.62 (80.51-239.14) | 0.75 | 5.76 (2.73-11.07) | 4.8 (2.67-7.93) | -0.61 (-0.91--0.3) |
| Lesotho | 88.28 (38.29-182.19) | 335.04 (176.72-567.99) | 2.8 | 13.31 (5.77-27.46) | 33.36 (17.6-56.55) | 3.56 (3.04-4.08) |
| Liberia | 181.91 (104.65-294.39) | 630.69 (356.81-1065.34) | 2.47 | 16.8 (9.66-27.18) | 22.49 (12.73-38) | 1.08 (0.63-1.53) |
| Libya | 153.84 (75.8-313.67) | 668.42 (351.23-1261.84) | 3.34 | 7.77 (3.83-15.85) | 16.21 (8.52-30.61) | 3.22 (2.66-3.78) |
| Lithuania | 262.07 (151-427.62) | 683.04 (387.5-1099.34) | 1.61 | 14.31 (8.25-23.36) | 59.12 (33.54-95.16) | 4.96 (3.74-6.19) |
| Luxembourg | 77.21 (44.63-123.34) | 53.24 (30.62-85.55) | -0.31 | 38.82 (22.44-62.02) | 16.78 (9.65-26.96) | -2.61 (-2.93--2.28) |
| Madagascar | 544.73 (335.5-847.24) | 1603.48 (892.5-2740.85) | 1.94 | 10.27 (6.33-15.97) | 11.36 (6.32-19.41) | 0.37 (0.3-0.45) |
| Malawi | 782.78 (475.55-1204.64) | 2086.54 (1232.4-3361.16) | 1.67 | 17.78 (10.8-27.37) | 21.55 (12.73-34.72) | 0.47 (0.27-0.67) |
| Malaysia | 373.17 (230.92-598.97) | 1301.9 (752.38-2126.34) | 2.49 | 4.14 (2.56-6.65) | 7.33 (4.24-11.98) | 1.21 (0.96-1.46) |
| Maldives | 5.3 (2.57-9.1) | 12.95 (6.81-21.41) | 1.44 | 5.56 (2.7-9.56) | 3.82 (2.01-6.32) | -1.33 (-1.63--1.03) |
| Mali | 457.38 (233.67-766.56) | 1410.06 (772.54-2270.49) | 2.08 | 12.64 (6.46-21.19) | 13.42 (7.35-21.62) | 0.59 (0.38-0.79) |
| Malta | 25.86 (14.88-42.06) | 30.39 (17.54-48.27) | 0.18 | 13.5 (7.76-21.95) | 15.61 (9.02-24.81) | 0.14 (-0.15-0.43) |
| Marshall Islands | 4.28 (2.6-6.64) | 8.33 (4.01-14.67) | 0.95 | 21.35 (12.95-33.06) | 27.33 (13.16-48.13) | 0.57 (0.47-0.68) |
| Mauritania | 149.55 (88.19-246.04) | 361.8 (205.3-618.01) | 1.42 | 16.4 (9.67-26.98) | 17.59 (9.98-30.05) | 0.18 (0.11-0.26) |
| Mauritius | 60.08 (35.13-94.88) | 83.83 (46.73-135.38) | 0.4 | 9.92 (5.8-15.67) | 13.1 (7.3-21.16) | -0.47 (-0.99-0.05) |
| Mexico | 31217.04 (19653.67-47027.6) | 79189.27 (50152.76-116156.21) | 1.54 | 73.39 (46.21-110.57) | 115.66 (73.25-169.65) | 1.46 (1.28-1.65) |
| Micronesia (Federated States of) | 14.32 (7.77-23.68) | 18.1 (9.51-32.17) | 0.26 | 30.25 (16.41-50.02) | 33.61 (17.66-59.74) | 0.04 (-0.18-0.27) |
| Monaco | 5.38 (3.14-9.03) | 7.22 (3.86-12.04) | 0.34 | 39.05 (22.8-65.46) | 51.38 (27.49-85.7) | 1.03 (0.7-1.36) |
| Mongolia | 292.52 (175.62-473.73) | 601.63 (342.65-985.14) | 1.06 | 28.49 (17.1-46.13) | 35.67 (20.32-58.41) | 0.97 (0.69-1.25) |
| Montenegro | 29.18 (16.85-47.52) | 32.48 (18.21-55.72) | 0.11 | 9.15 (5.28-14.9) | 11.12 (6.24-19.08) | 0.82 (0.59-1.04) |
| Morocco | 596.12 (324.84-982.65) | 1540.82 (802.18-2846.78) | 1.58 | 4.79 (2.61-7.9) | 7.93 (4.13-14.65) | 1.57 (1.5-1.63) |
| Mozambique | 329.36 (191.16-541.17) | 1169.62 (630-2022.3) | 2.55 | 5.7 (3.31-9.36) | 8.2 (4.42-14.18) | 1.83 (1.61-2.05) |
| Myanmar | 2804.36 (1386.65-5199.47) | 4319.85 (2096.58-7435.92) | 0.54 | 13.74 (6.79-25.47) | 14.69 (7.13-25.29) | 0.11 (0.06-0.15) |
| Namibia | 82.69 (38.18-167.32) | 218.62 (109.93-420.04) | 1.64 | 12.56 (5.8-25.41) | 16.94 (8.52-32.54) | 0.69 (0.34-1.04) |
| Nauru | 1.84 (0.84-3.34) | 1.97 (0.78-3.73) | 0.07 | 36.98 (16.82-67.25) | 34.26 (13.58-64.86) | -0.69 (-0.86--0.51) |
| Nepal | 1036.86 (590.86-1755.84) | 2948.19 (1665.59-4823.84) | 1.84 | 11.65 (6.64-19.73) | 17.71 (10.01-28.98) | 1.69 (1.46-1.91) |
| Netherlands | 1008.53 (586.19-1582.45) | 612.59 (360.57-926.2) | -0.39 | 12.44 (7.23-19.52) | 8.27 (4.87-12.51) | -1.94 (-2.41--1.47) |
| New Zealand | 104.43 (66.56-150.95) | 154.56 (102.24-221) | 0.48 | 5.79 (3.69-8.36) | 6.35 (4.2-9.08) | 0.55 (0.35-0.76) |
| Nicaragua | 462.61 (282.14-718) | 1795.16 (1027.82-2920.72) | 2.88 | 26.76 (16.32-41.54) | 49.97 (28.61-81.31) | 2.22 (1.99-2.45) |
| Niger | 443.59 (247.89-793.11) | 1168.05 (582.56-2192.2) | 1.63 | 13.25 (7.4-23.69) | 11.26 (5.62-21.14) | -0.29 (-0.39--0.19) |
| Nigeria | 5487.05 (2983.96-9255.68) | 13509.54 (7170.55-22908.61) | 1.46 | 13.38 (7.28-22.57) | 12.52 (6.65-21.24) | -0.01 (-0.11-0.1) |
| Niue | 0.25 (0.14-0.43) | 0.26 (0.15-0.44) | 0.04 | 23.88 (13.73-42.05) | 33.6 (18.61-56.75) | 0.51 (0.29-0.74) |
| North Macedonia | 77.99 (45.87-125.42) | 95.22 (51.58-170.85) | 0.22 | 7.55 (4.44-12.15) | 8.65 (4.69-15.52) | 0.3 (0-0.61) |
| Northern Mariana Islands | 14.75 (8.13-26.44) | 11.92 (6.89-19.04) | -0.19 | 50.8 (28-91.08) | 50.27 (29.06-80.27) | -0.22 (-0.49-0.06) |
| Norway | 188.01 (115.75-283.68) | 169.58 (106.06-251.8) | -0.1 | 8.68 (5.34-13.09) | 6.79 (4.25-10.08) | -1.07 (-1.45--0.68) |
| Oman | 42.96 (22.51-75.03) | 197.64 (100.63-364.03) | 3.6 | 4.32 (2.26-7.54) | 6.6 (3.36-12.16) | 1.96 (1.76-2.16) |
| Pakistan | 6634.27 (3120.48-13183.7) | 18101.04 (10383.28-28144.56) | 1.73 | 13.42 (6.31-26.66) | 14.84 (8.51-23.07) | 0.16 (0.05-0.27) |
| Palau | 2.2 (1.08-4.13) | 4.4 (2.16-7.44) | 1 | 25.65 (12.6-48.24) | 48.53 (23.88-82.07) | 1.86 (1.64-2.08) |
| Palestine | 32.22 (17-60.71) | 67.89 (39.16-108.47) | 1.11 | 3.63 (1.92-6.84) | 2.56 (1.47-4.09) | -1.26 (-1.42--1.11) |
| Panama | 173.23 (106.23-266.54) | 429.55 (242.8-701.37) | 1.48 | 14.06 (8.62-21.63) | 19.85 (11.22-32.4) | 1.2 (1.06-1.34) |
| Papua New Guinea | 112.51 (55.25-195.21) | 258.2 (143.48-430.3) | 1.29 | 5.66 (2.78-9.82) | 4.84 (2.69-8.06) | -0.88 (-1.09--0.66) |
| Paraguay | 165.67 (101.91-253.87) | 508.64 (276.61-818.48) | 2.07 | 8.76 (5.39-13.43) | 13.18 (7.17-21.21) | 1.59 (1.47-1.71) |
| Peru | 4815.68 (2874.02-7271.59) | 9429.23 (5520.87-14963.62) | 0.96 | 45.05 (26.88-68.02) | 48.78 (28.56-77.42) | 0.03 (-0.29-0.34) |
| Philippines | 1966.72 (1217.31-3091.4) | 4038.82 (2372.26-6422.79) | 1.05 | 6.32 (3.91-9.94) | 6.73 (3.95-10.7) | 0.11 (0.02-0.19) |
| Poland | 1855.25 (1158.94-2845.66) | 6692.65 (4173.45-10215.49) | 2.61 | 9.78 (6.11-15.01) | 37.34 (23.29-57) | 3.44 (2.93-3.96) |
| Portugal | 2394.45 (1428.53-3714.65) | 1197.81 (691.06-1914.85) | -0.5 | 47.88 (28.56-74.28) | 26.15 (15.09-41.81) | -2.41 (-2.98--1.84) |
| Puerto Rico | 1486 (900.1-2269.65) | 761.69 (459.16-1139.85) | -0.49 | 80.55 (48.79-123.03) | 51.99 (31.34-77.81) | -2.17 (-2.6--1.75) |
| Qatar | 27.67 (15.86-44.42) | 229.08 (119.3-382.13) | 7.28 | 9.49 (5.44-15.24) | 10.45 (5.44-17.42) | -0.26 (-0.88-0.37) |
| Republic of Korea | 2518.92 (1370.64-4186.33) | 1007.92 (519.84-1761.65) | -0.6 | 9.74 (5.3-16.19) | 4.15 (2.14-7.25) | -2.89 (-3--2.77) |
| Republic of Moldova | 1520.93 (881.83-2500.19) | 2054.56 (1172.62-3405.89) | 0.35 | 69.23 (40.14-113.8) | 114.79 (65.51-190.28) | 1.51 (1.09-1.92) |
| Romania | 2769.4 (1653.74-4358.81) | 3768.8 (2094.98-6353.37) | 0.36 | 24.43 (14.59-38.44) | 45.24 (25.15-76.26) | 0.9 (0.37-1.43) |
| Russian Federation | 7963.51 (4915.44-12393.48) | 77997.43 (46552.11-122123.96) | 8.79 | 10.73 (6.62-16.7) | 115.66 (69.03-181.09) | 7.32 (6.01-8.64) |
| Rwanda | 545.12 (285.84-946.31) | 1031.26 (539.92-1821.3) | 0.89 | 17.27 (9.06-29.99) | 15.05 (7.88-26.58) | -1.47 (-1.92--1.02) |
| Saint Kitts and Nevis | 8.09 (4.96-12.17) | 11.51 (6.26-19.5) | 0.42 | 40.64 (24.91-61.18) | 36.45 (19.83-61.73) | -0.88 (-1.67--0.08) |
| Saint Lucia | 23.74 (14.57-36.52) | 45.71 (25.58-72.59) | 0.93 | 35.66 (21.88-54.84) | 49.42 (27.65-78.49) | 1.2 (0.85-1.56) |
| Saint Vincent and the Grenadines | 9.46 (5.85-14.61) | 24.94 (14.19-40.21) | 1.64 | 17.72 (10.94-27.35) | 44.16 (25.13-71.2) | 3.01 (2.63-3.38) |
| Samoa | 15.08 (8.08-25.73) | 22.2 (11.69-39.54) | 0.47 | 19.25 (10.31-32.85) | 22.14 (11.65-39.42) | 0.23 (0.1-0.36) |
| San Marino | 3.11 (1.79-5.08) | 3.29 (1.58-6.51) | 0.06 | 24.96 (14.41-40.78) | 23.94 (11.48-47.42) | 1.11 (0.59-1.64) |
| Sao Tome and Principe | 12.22 (6.33-20.69) | 35.94 (17.46-66.83) | 1.94 | 24.43 (12.66-41.36) | 31.84 (15.47-59.2) | 0.73 (0.43-1.03) |
| Saudi Arabia | 923.74 (457.55-1805.15) | 6798.06 (3372.26-11439.9) | 6.36 | 11.52 (5.71-22.51) | 26.87 (13.33-45.22) | 3 (2.81-3.19) |
| Senegal | 428.34 (243-706.44) | 1068.92 (559.07-1852.92) | 1.5 | 13.12 (7.44-21.64) | 13.82 (7.23-23.95) | 0.43 (0.2-0.66) |
| Serbia | 547.11 (312.03-884.02) | 454.78 (249.62-786.76) | -0.17 | 11.48 (6.55-18.56) | 10.73 (5.89-18.56) | -0.36 (-0.56--0.16) |
| Seychelles | 5.98 (3.43-9.5) | 12.88 (7.06-21.57) | 1.15 | 16.14 (9.25-25.65) | 23.8 (13.04-39.84) | 0.94 (0.6-1.28) |
| Sierra Leone | 222.14 (117.86-387.99) | 588.86 (329.01-956.23) | 1.65 | 11.69 (6.2-20.42) | 13.24 (7.4-21.5) | 0.59 (0.48-0.71) |
| Singapore | 76.68 (42.85-126.75) | 55.08 (30.34-90.53) | -0.28 | 4.03 (2.25-6.67) | 1.85 (1.02-3.04) | -2.92 (-3.13--2.7) |
| Slovakia | 714.35 (395.25-1190.02) | 700.1 (398.27-1209.23) | -0.02 | 26.74 (14.79-44.54) | 27.07 (15.4-46.75) | 0.15 (-0.06-0.36) |
| Slovenia | 244.52 (139.17-390.44) | 100.67 (55.66-164.95) | -0.59 | 24.11 (13.72-38.49) | 11.48 (6.34-18.8) | -3.02 (-3.66--2.37) |
| Solomon Islands | 25.54 (11.97-47.04) | 95.11 (55.14-163.61) | 2.72 | 16.85 (7.9-31.04) | 27.62 (16.01-47.51) | 1.65 (1.46-1.83) |
| Somalia | 720.77 (373.21-1245.88) | 1831.46 (895.09-3397.5) | 1.54 | 20.63 (10.68-35.66) | 18.38 (8.98-34.1) | -0.9 (-1.1--0.7) |
| South Africa | 4326.09 (2844.54-6506.64) | 8898.63 (5557.43-13477.14) | 1.06 | 22.93 (15.08-34.49) | 28.56 (17.84-43.26) | 0.57 (-0.04-1.18) |
| South Sudan | 361 (189.49-635.68) | 975.8 (532.79-1697.26) | 1.7 | 13.37 (7.02-23.54) | 21.93 (11.98-38.15) | 1.71 (1.38-2.05) |
| Spain | 6062.1 (3705.36-9306.95) | 3070.6 (1813.23-4694.79) | -0.49 | 31.21 (19.08-47.92) | 15.36 (9.07-23.49) | -2.52 (-2.86--2.18) |
| Sri Lanka | 1101.57 (632.39-1815.56) | 708.58 (332.16-1295.97) | -0.36 | 11.99 (6.88-19.77) | 6.43 (3.01-11.75) | -2.99 (-3.52--2.45) |
| Sudan | 535.56 (284.74-989.01) | 1571.36 (748.7-3074.56) | 1.93 | 5.88 (3.12-10.85) | 7.03 (3.35-13.75) | 0.87 (0.69-1.05) |
| Suriname | 76.12 (44.53-117.23) | 142.75 (79.67-228.46) | 0.88 | 38.5 (22.52-59.29) | 49.7 (27.74-79.55) | 0.89 (0.65-1.13) |
| Sweden | 345.13 (189.07-574.7) | 156.58 (82.05-270.11) | -0.55 | 8.21 (4.5-13.68) | 3.46 (1.81-5.97) | -2.18 (-2.42--1.95) |
| Switzerland | 701.78 (422.03-1115.54) | 295.15 (174.48-464.34) | -0.58 | 19.34 (11.63-30.75) | 7.37 (4.36-11.59) | -2.94 (-3.35--2.53) |
| Syrian Arab Republic | 368.16 (211.69-622.92) | 590.21 (320.89-1132.47) | 0.6 | 6.57 (3.78-11.12) | 8.31 (4.52-15.95) | 1.03 (0.55-1.51) |
| Taiwan (Province of China) | 893.31 (522.98-1399.54) | 1142.66 (628.09-1902.01) | 0.28 | 7.94 (4.65-12.43) | 10.05 (5.52-16.72) | 0.75 (0.31-1.19) |
| Tajikistan | 405.27 (242.25-635.99) | 928.38 (516.1-1594.33) | 1.29 | 16.81 (10.05-26.39) | 18.06 (10.04-31.01) | -0.63 (-1--0.27) |
| Thailand | 3336.7 (1986.92-5384.12) | 10411.54 (5242.16-18104.73) | 2.12 | 10.54 (6.28-17.01) | 32.62 (16.42-56.72) | 3.78 (3.4-4.16) |
| Timor-Leste | 22.63 (11.44-43.78) | 33.67 (16.58-61.6) | 0.49 | 5.91 (2.99-11.44) | 4.88 (2.4-8.93) | -0.91 (-1.35--0.46) |
| Togo | 179.35 (101.93-297.27) | 649.13 (351.34-1181.74) | 2.62 | 11.15 (6.34-18.49) | 15.62 (8.45-28.43) | 1.4 (1.26-1.53) |
| Tokelau | 0.13 (0.07-0.23) | 0.17 (0.1-0.27) | 0.31 | 18.52 (10.01-32.6) | 25.44 (14.7-40.89) | 0.33 (0.09-0.57) |
| Tonga | 13.4 (7.86-21.59) | 18.88 (9.85-32.91) | 0.41 | 30.26 (17.76-48.76) | 38.37 (20.01-66.9) | 0.57 (0.31-0.84) |
| Trinidad and Tobago | 202.58 (124.35-314.24) | 287.26 (158.16-467.95) | 0.42 | 32.75 (20.1-50.8) | 41.63 (22.92-67.81) | 0.77 (0.48-1.07) |
| Tunisia | 225.93 (126.33-374.31) | 616.33 (318.97-1147.5) | 1.73 | 5.52 (3.08-9.14) | 10.2 (5.28-18.98) | 2.17 (2.08-2.25) |
| Turkey | 1021.06 (579.24-1669.74) | 1383.65 (776.8-2351.34) | 0.36 | 3.52 (2-5.76) | 3.15 (1.77-5.35) | -0.53 (-0.81--0.26) |
| Turkmenistan | 479.09 (297.21-754.42) | 4259.51 (2567.15-7212.64) | 7.89 | 27.25 (16.9-42.91) | 159.13 (95.91-269.45) | 5.98 (5.69-6.28) |
| Tuvalu | 1.15 (0.66-1.87) | 1.59 (0.86-2.69) | 0.38 | 25.55 (14.72-41.33) | 25.5 (13.73-43.02) | -0.32 (-0.48--0.16) |
| Uganda | 608.65 (349.85-1016.62) | 2278.31 (1264.93-3739.83) | 2.74 | 8.19 (4.71-13.68) | 11.33 (6.29-18.6) | 0.57 (0.37-0.76) |
| Ukraine | 4157.7 (2533.85-6529.98) | 21222.49 (11531.63-35814.09) | 4.1 | 16.66 (10.16-26.17) | 104.93 (57.01-177.07) | 5.19 (3.92-6.48) |
| United Arab Emirates | 64.9 (35.27-108.4) | 592.41 (314.73-971.7) | 8.13 | 5.51 (2.99-9.2) | 8.63 (4.59-14.16) | 1.47 (1.17-1.77) |
| United Kingdom | 6198.9 (3923.95-9092.23) | 15765.77 (10462.07-21651.8) | 1.54 | 21.78 (13.79-31.95) | 51.96 (34.48-71.35) | 2.72 (1.8-3.65) |
| United Republic of Tanzania | 1171.56 (701.61-1942.9) | 3919.86 (2122.04-6555.47) | 2.35 | 10.36 (6.2-17.18) | 13.9 (7.53-23.25) | 1.19 (1.03-1.35) |
| United States of America | 23247.98 (14382.59-35556.66) | 29091.46 (17922.47-45557.08) | 0.25 | 17.32 (10.71-26.49) | 19.14 (11.79-29.97) | 0.19 (0.05-0.33) |
| United States Virgin Islands | 35.07 (20.88-55.21) | 29.7 (17.81-46.93) | -0.15 | 63.92 (38.05-100.62) | 87.86 (52.67-138.82) | 1.51 (1.32-1.69) |
| Uruguay | 198.49 (114.46-320.26) | 199.09 (118.89-316.95) | 0 | 13.36 (7.7-21.55) | 12.13 (7.24-19.31) | -0.15 (-0.34-0.04) |
| Uzbekistan | 2309.19 (1453.34-3463.6) | 10128.67 (5660.35-16029.51) | 3.39 | 23.61 (14.86-35.41) | 56.79 (31.74-89.88) | 2.11 (1.67-2.56) |
| Vanuatu | 17.02 (7.95-31.54) | 45.09 (23.58-83.06) | 1.65 | 24.24 (11.32-44.91) | 28.9 (15.11-53.23) | 0.3 (0.21-0.38) |
| Venezuela (Bolivarian Republic of) | 2396.47 (1438.76-3772.89) | 4837.84 (2551.67-8254.4) | 1.02 | 24.99 (15-39.34) | 36.79 (19.41-62.78) | 0.89 (0.5-1.28) |
| Viet Nam | 1760.92 (1033.23-3027.28) | 3681.64 (1883.99-6449.17) | 1.09 | 5.37 (3.15-9.23) | 7.07 (3.62-12.38) | 0.97 (0.83-1.11) |
| Yemen | 242.52 (109.46-475.78) | 566.02 (307.07-1014.42) | 1.33 | 4.46 (2.01-8.74) | 3.38 (1.84-6.07) | -1.15 (-1.3--1) |
| Zambia | 648.98 (390.43-1016.77) | 1947.77 (934.59-3481.14) | 2 | 18.37 (11.05-28.78) | 20.16 (9.67-36.03) | -0.03 (-0.15-0.1) |
| Zimbabwe | 363.82 (219.28-586.45) | 1401.66 (587.83-2699.71) | 2.85 | 7.89 (4.76-12.72) | 18.1 (7.59-34.86) | 2.92 (2.23-3.61) |
