# Supplementary material for: Global, regional and national burden of Metabolic dysfunction-associated steatotic liver disease in adolescents and adults aged 15–49 years from 1990 to 2021: results from the 2021 Global Burden of Disease study
Source: Front Med (Lausanne). 2025 Jun 25;12:1568211. doi: 10.3389/fmed.2025.1568211 (PMC12237898; doi:10.3389/fmed.2025.1568211)
Supplement: Supplementary file 1 [file Supplementary_file_1.ZIP › Supplementary Table 7.docx]

**Supplementary Table 7** The death of MASLD cases and rates in the adolescents and adults aged 15-49 years in 1990 and 2021 across 204 countries, and the trends from 1990 to 2021.

| **location** | **Death cases** | | | **Death rates** | | |
| --- | --- | --- | --- | --- | --- | --- |
|  | **1990**  **(95%UI)** | **2021**  **(95%UI)** | **percentage**  **Change**  **(100%)** | **1990**  **Per 100,000**  **(95%UI)** | **2021**  **Per 100,000**  **(95%UI)** | **EAPC**  **(95% CI)** |
| Afghanistan | 9.7 (4.48-18.89) | 24.8 (9.72-50.47) | 1.56 | 0.24 (0.11-0.47) | 0.17 (0.07-0.34) | -0.53 (-1.06-0) |
| Albania | 1.74 (1.04-2.78) | 1.83 (0.99-3.18) | 0.05 | 0.1 (0.06-0.16) | 0.15 (0.08-0.25) | 1.57 (1.29-1.85) |
| Algeria | 9.58 (5.29-15.58) | 31.8 (17.39-55.26) | 2.32 | 0.08 (0.04-0.13) | 0.14 (0.08-0.24) | 2.02 (1.82-2.23) |
| American Samoa | 0.12 (0.07-0.2) | 0.18 (0.1-0.3) | 0.5 | 0.51 (0.29-0.83) | 0.77 (0.43-1.24) | 1.07 (0.92-1.23) |
| Andorra | 0.17 (0.08-0.31) | 0.24 (0.11-0.43) | 0.41 | 0.52 (0.26-0.95) | 0.58 (0.27-1.03) | 0.85 (0.58-1.13) |
| Angola | 17.9 (10.21-31.62) | 52.07 (27.21-86.07) | 1.91 | 0.38 (0.22-0.68) | 0.36 (0.19-0.59) | 0 (-0.18-0.18) |
| Antigua and Barbuda | 0.12 (0.07-0.2) | 0.19 (0.11-0.32) | 0.58 | 0.39 (0.23-0.63) | 0.41 (0.24-0.66) | 1.05 (0.57-1.54) |
| Argentina | 60.27 (34.55-96.67) | 61.41 (35.08-102.52) | 0.02 | 0.38 (0.22-0.61) | 0.26 (0.15-0.44) | -0.81 (-1.13--0.5) |
| Armenia | 3.63 (2.23-5.58) | 5.47 (3.03-8.52) | 0.51 | 0.21 (0.13-0.33) | 0.38 (0.21-0.59) | 2.36 (1.54-3.18) |
| Australia | 21.2 (12.57-32.16) | 36.96 (23.12-54.45) | 0.74 | 0.24 (0.14-0.36) | 0.31 (0.19-0.45) | 1.15 (0.8-1.5) |
| Austria | 36.13 (20.76-57.45) | 17.76 (10.71-27.47) | -0.51 | 0.9 (0.52-1.43) | 0.44 (0.27-0.68) | -2.09 (-2.42--1.76) |
| Azerbaijan | 15.45 (9.56-24.1) | 32.85 (18.21-54.68) | 1.13 | 0.42 (0.26-0.66) | 0.59 (0.33-0.98) | 0.92 (0.62-1.22) |
| Bahamas | 1.32 (0.8-2.03) | 2.48 (1.45-4.08) | 0.88 | 0.93 (0.57-1.42) | 1.18 (0.69-1.94) | 0.94 (0.7-1.18) |
| Bahrain | 0.35 (0.2-0.56) | 1.97 (1.12-3.28) | 4.63 | 0.11 (0.06-0.19) | 0.2 (0.11-0.34) | 1.14 (0.76-1.52) |
| Bangladesh | 120.02 (73.96-187.31) | 171.42 (95.66-288.45) | 0.43 | 0.24 (0.15-0.37) | 0.19 (0.11-0.33) | -0.51 (-0.67--0.34) |
| Barbados | 0.85 (0.5-1.36) | 0.7 (0.37-1.16) | -0.18 | 0.63 (0.37-1.01) | 0.5 (0.26-0.83) | -0.88 (-1.16--0.59) |
| Belarus | 6.97 (4.07-11.08) | 41.02 (21.67-69.01) | 4.89 | 0.14 (0.08-0.22) | 0.96 (0.51-1.62) | 6.19 (4.75-7.66) |
| Belgium | 33.36 (18.26-54.09) | 29.14 (17.19-42.99) | -0.13 | 0.67 (0.37-1.09) | 0.58 (0.34-0.86) | -1.29 (-1.78--0.79) |
| Belize | 0.36 (0.21-0.55) | 2.73 (1.61-4.28) | 6.58 | 0.42 (0.25-0.64) | 1.15 (0.68-1.81) | 3.05 (2.6-3.5) |
| Benin | 5.53 (3.08-9.33) | 20.45 (10.61-36.11) | 2.7 | 0.28 (0.15-0.47) | 0.33 (0.17-0.58) | 0.63 (0.51-0.75) |
| Bermuda | 0.25 (0.15-0.37) | 0.17 (0.1-0.28) | -0.32 | 0.73 (0.44-1.09) | 0.65 (0.37-1.05) | -0.31 (-0.91-0.29) |
| Bhutan | 0.85 (0.41-1.52) | 1.8 (0.91-3.22) | 1.12 | 0.27 (0.13-0.49) | 0.41 (0.21-0.74) | 1.08 (0.99-1.17) |
| Bolivia (Plurinational State of) | 30.49 (11.68-53.49) | 67.4 (34.77-116.12) | 1.21 | 1.02 (0.39-1.79) | 1.08 (0.56-1.86) | -0.28 (-0.53--0.04) |
| Bosnia and Herzegovina | 4.63 (2.6-7.57) | 3.79 (2-6.66) | -0.18 | 0.19 (0.11-0.32) | 0.25 (0.13-0.45) | 0.7 (0.42-0.98) |
| Botswana | 1.69 (0.77-3.48) | 4.67 (2.55-8.47) | 1.76 | 0.28 (0.13-0.58) | 0.34 (0.19-0.62) | 0.52 (0.28-0.75) |
| Brazil | 316.11 (202.87-474.88) | 518.68 (320.36-775.93) | 0.64 | 0.41 (0.26-0.62) | 0.45 (0.28-0.67) | 0.14 (-0.07-0.35) |
| Brunei Darussalam | 0.13 (0.07-0.21) | 0.31 (0.17-0.55) | 1.38 | 0.09 (0.05-0.14) | 0.11 (0.06-0.2) | 1.05 (0.79-1.31) |
| Bulgaria | 15.63 (8.78-25.27) | 22.81 (12.09-40.16) | 0.46 | 0.38 (0.21-0.61) | 0.78 (0.41-1.37) | 1.98 (1.48-2.48) |
| Burkina Faso | 6.24 (3.54-10.23) | 19.87 (10.45-32.48) | 2.18 | 0.16 (0.09-0.27) | 0.19 (0.1-0.32) | 0.93 (0.78-1.07) |
| Burundi | 7.15 (3.88-12.53) | 13.39 (7.38-21.43) | 0.87 | 0.29 (0.16-0.52) | 0.21 (0.12-0.34) | -1.77 (-2.04--1.5) |
| Cabo Verde | 0.35 (0.21-0.58) | 1.01 (0.55-1.72) | 1.89 | 0.24 (0.15-0.39) | 0.32 (0.18-0.55) | 0.87 (0.66-1.08) |
| Cambodia | 20.33 (11.59-35.6) | 35.16 (19.04-62.14) | 0.73 | 0.44 (0.25-0.77) | 0.39 (0.21-0.69) | -0.66 (-0.75--0.57) |
| Cameroon | 16.64 (9.16-27.29) | 67.87 (35.04-120.76) | 3.08 | 0.37 (0.2-0.6) | 0.44 (0.23-0.78) | 0.72 (0.61-0.83) |
| Canada | 42.12 (25.11-66.91) | 90.07 (54.57-133.7) | 1.14 | 0.29 (0.17-0.45) | 0.54 (0.33-0.8) | 2.19 (1.9-2.48) |
| Central African Republic | 5.01 (2.59-8.42) | 12.24 (6.65-21.55) | 1.44 | 0.4 (0.21-0.68) | 0.46 (0.25-0.81) | 0.45 (0.36-0.53) |
| Chad | 5.42 (3.04-9.26) | 19.29 (10.66-34.28) | 2.56 | 0.22 (0.12-0.37) | 0.26 (0.14-0.46) | 0.76 (0.64-0.87) |
| Chile | 66.95 (39.77-109.22) | 63.2 (37.49-100.25) | -0.06 | 0.94 (0.56-1.54) | 0.67 (0.39-1.05) | -0.11 (-0.46-0.25) |
| China | 821.8 (499.85-1261.82) | 615.25 (345.2-982.76) | -0.25 | 0.12 (0.07-0.19) | 0.09 (0.05-0.15) | -0.84 (-0.99--0.69) |
| Colombia | 31.3 (18.67-49.34) | 51.88 (28.96-84.1) | 0.66 | 0.19 (0.11-0.29) | 0.2 (0.11-0.32) | -0.04 (-0.18-0.1) |
| Comoros | 0.61 (0.3-1.04) | 1.44 (0.77-2.43) | 1.36 | 0.3 (0.15-0.51) | 0.37 (0.2-0.62) | 0.36 (-0.02-0.75) |
| Congo | 5.19 (2.76-8.95) | 16.62 (8.43-31.29) | 2.2 | 0.47 (0.25-0.81) | 0.59 (0.3-1.11) | 0.8 (0.64-0.96) |
| Cook Islands | 0.01 (0-0.01) | 0.01 (0-0.01) | 0 | 0.06 (0.03-0.11) | 0.1 (0.05-0.17) | 1.59 (1.35-1.83) |
| Costa Rica | 7.8 (4.67-11.99) | 20.64 (12-32.92) | 1.65 | 0.51 (0.3-0.78) | 0.82 (0.48-1.31) | 0.76 (0.46-1.05) |
| C么te d'Ivoire | 14.82 (8.58-24.35) | 45.32 (24.93-79.15) | 2.06 | 0.27 (0.15-0.44) | 0.33 (0.18-0.58) | 0.89 (0.78-1) |
| Croatia | 12.03 (6.72-19.78) | 5.52 (2.99-9.34) | -0.54 | 0.49 (0.28-0.81) | 0.3 (0.16-0.51) | -1.9 (-2.24--1.56) |
| Cuba | 19.87 (12.06-31.05) | 46.33 (25.84-76.04) | 1.33 | 0.32 (0.2-0.5) | 0.91 (0.51-1.5) | 3.85 (3.48-4.21) |
| Cyprus | 1.31 (0.68-2.19) | 1.77 (0.99-2.95) | 0.35 | 0.32 (0.17-0.54) | 0.25 (0.14-0.42) | -1.36 (-1.63--1.08) |
| Czechia | 20.87 (11.86-34.19) | 24.74 (13.8-41.07) | 0.19 | 0.4 (0.23-0.66) | 0.52 (0.29-0.87) | 0.59 (0.35-0.82) |
| Democratic People's Republic of Korea | 13.1 (7.01-24.63) | 15.99 (6.61-30.66) | 0.22 | 0.12 (0.07-0.23) | 0.12 (0.05-0.22) | 0.11 (0.03-0.19) |
| Democratic Republic of the Congo | 45.68 (25.13-75.35) | 116.56 (62.12-192.98) | 1.55 | 0.27 (0.15-0.45) | 0.27 (0.14-0.45) | -0.06 (-0.16-0.03) |
| Denmark | 16.36 (9.07-26.22) | 7.78 (4.39-12.19) | -0.52 | 0.61 (0.34-0.98) | 0.3 (0.17-0.47) | -3.23 (-4.04--2.42) |
| Djibouti | 0.4 (0.2-0.73) | 2.08 (1.05-3.71) | 4.2 | 0.19 (0.1-0.35) | 0.3 (0.15-0.54) | 1.4 (1.23-1.57) |
| Dominica | 0.15 (0.09-0.23) | 0.24 (0.13-0.4) | 0.6 | 0.42 (0.25-0.66) | 0.71 (0.4-1.19) | 1.92 (1.73-2.11) |
| Dominican Republic | 24.48 (14.68-39.5) | 61.85 (30.3-108.91) | 1.53 | 0.67 (0.4-1.09) | 1.06 (0.52-1.86) | 1.96 (1.61-2.31) |
| Ecuador | 60.01 (38.65-90.18) | 93.94 (58.38-143.36) | 0.57 | 1.21 (0.78-1.82) | 1 (0.62-1.53) | -0.73 (-1.06--0.4) |
| Egypt | 103.51 (63.83-165.51) | 190.02 (106.51-316.6) | 0.84 | 0.39 (0.24-0.62) | 0.36 (0.2-0.59) | -0.4 (-0.62--0.19) |
| El Salvador | 21.96 (12.95-34.87) | 46.96 (24.94-78.57) | 1.14 | 0.88 (0.52-1.39) | 1.42 (0.75-2.37) | 1.59 (1.38-1.79) |
| Equatorial Guinea | 0.65 (0.32-1.11) | 3.25 (1.52-6.28) | 4 | 0.36 (0.18-0.61) | 0.4 (0.19-0.77) | 0.57 (0.14-1.01) |
| Eritrea | 4.54 (2.45-7.71) | 10.05 (5.5-18.52) | 1.21 | 0.29 (0.16-0.5) | 0.29 (0.16-0.54) | -0.06 (-0.14-0.02) |
| Estonia | 2.3 (1.28-3.79) | 5.51 (3.06-9.02) | 1.4 | 0.3 (0.17-0.5) | 0.96 (0.53-1.57) | 3.13 (2.28-3.99) |
| Eswatini | 1.77 (0.92-3.2) | 5.61 (2.86-10.55) | 2.17 | 0.5 (0.26-0.9) | 0.91 (0.46-1.71) | 1.99 (1.32-2.67) |
| Ethiopia | 57.78 (26.73-100.89) | 103.42 (64.64-164.62) | 0.79 | 0.27 (0.12-0.46) | 0.19 (0.12-0.3) | -1.58 (-1.79--1.36) |
| Fiji | 0.83 (0.5-1.35) | 1.27 (0.66-2.19) | 0.53 | 0.21 (0.13-0.34) | 0.27 (0.14-0.47) | 0.63 (0.52-0.74) |
| Finland | 17.33 (10.12-28.08) | 12.92 (7.65-19.97) | -0.25 | 0.67 (0.39-1.09) | 0.55 (0.33-0.85) | -0.21 (-0.98-0.57) |
| France | 182.64 (102.84-297.92) | 88.77 (50.98-145) | -0.51 | 0.63 (0.35-1.02) | 0.31 (0.18-0.51) | -2.5 (-2.93--2.06) |
| Gabon | 1.87 (1.06-3.15) | 6.42 (3.39-11.97) | 2.43 | 0.42 (0.24-0.7) | 0.69 (0.37-1.29) | 1.65 (1.4-1.91) |
| Gambia | 0.87 (0.45-1.44) | 3.3 (1.63-5.65) | 2.79 | 0.2 (0.1-0.32) | 0.28 (0.14-0.48) | 1.18 (0.88-1.48) |
| Georgia | 13.94 (8.23-22.16) | 12.11 (6.79-20.03) | -0.13 | 0.52 (0.31-0.83) | 0.76 (0.42-1.25) | 2.15 (1.58-2.71) |
| Germany | 402.55 (229.45-631.98) | 212.41 (122.29-319.64) | -0.47 | 1.01 (0.58-1.58) | 0.6 (0.34-0.9) | -2.03 (-2.48--1.57) |
| Ghana | 19.15 (11.42-31.1) | 77.02 (41.66-127.65) | 3.02 | 0.28 (0.17-0.45) | 0.44 (0.24-0.73) | 1.56 (1.42-1.69) |
| Greece | 12.12 (7.28-19.04) | 14.7 (8.55-22.65) | 0.21 | 0.24 (0.14-0.38) | 0.34 (0.2-0.52) | 1.5 (1.06-1.95) |
| Greenland | 0.21 (0.12-0.35) | 0.18 (0.1-0.31) | -0.14 | 0.64 (0.35-1.05) | 0.67 (0.37-1.19) | 0.73 (0.21-1.26) |
| Grenada | 0.19 (0.12-0.3) | 0.36 (0.2-0.56) | 0.89 | 0.49 (0.3-0.77) | 0.67 (0.37-1.05) | 1.01 (0.76-1.26) |
| Guam | 0.33 (0.2-0.54) | 0.58 (0.34-0.97) | 0.76 | 0.42 (0.26-0.69) | 0.77 (0.45-1.3) | 2.34 (2.16-2.53) |
| Guatemala | 48.88 (29.55-75.79) | 141.51 (79.73-231.29) | 1.9 | 1.38 (0.84-2.14) | 1.68 (0.95-2.75) | 0.25 (-0.09-0.59) |
| Guinea | 6.99 (3.91-11.46) | 17.2 (10.18-29.86) | 1.46 | 0.28 (0.16-0.46) | 0.28 (0.17-0.49) | 0.18 (0.12-0.24) |
| Guinea-Bissau | 1.78 (0.97-3.11) | 4.19 (2.06-7.22) | 1.35 | 0.41 (0.22-0.71) | 0.42 (0.21-0.72) | 0.17 (0.02-0.33) |
| Guyana | 4.72 (2.76-7.55) | 7.24 (3.76-12.02) | 0.53 | 1.17 (0.68-1.87) | 1.81 (0.94-3.01) | 1.94 (1.59-2.3) |
| Haiti | 22.97 (11.08-37.74) | 42.13 (21.51-75.7) | 0.83 | 0.78 (0.38-1.29) | 0.61 (0.31-1.1) | -0.76 (-0.95--0.56) |
| Honduras | 20.17 (11.63-32.41) | 51.72 (22.6-96.07) | 1.56 | 0.98 (0.57-1.58) | 0.96 (0.42-1.77) | -0.51 (-0.71--0.31) |
| Hungary | 71.3 (39.7-113.96) | 23.54 (13.23-40.09) | -0.67 | 1.4 (0.78-2.24) | 0.54 (0.3-0.92) | -5.48 (-6.23--4.72) |
| Iceland | 0.16 (0.09-0.26) | 0.23 (0.13-0.37) | 0.44 | 0.12 (0.07-0.2) | 0.14 (0.08-0.22) | -0.19 (-0.46-0.07) |
| India | 1151.76 (732.35-1795.36) | 2265.44 (1297.29-3665.48) | 0.97 | 0.27 (0.17-0.43) | 0.29 (0.17-0.47) | 0.18 (0.03-0.33) |
| Indonesia | 328.22 (202.29-534.97) | 675.5 (422.93-1095.06) | 1.06 | 0.35 (0.21-0.57) | 0.44 (0.28-0.71) | 1.01 (0.88-1.14) |
| Iran (Islamic Republic of) | 47.73 (29.62-73.89) | 121.81 (76.48-184.89) | 1.55 | 0.19 (0.12-0.29) | 0.26 (0.16-0.39) | 1.09 (0.96-1.23) |
| Iraq | 12.61 (7.13-20.81) | 30.31 (15.53-53.79) | 1.4 | 0.15 (0.08-0.25) | 0.14 (0.07-0.24) | -0.07 (-0.24-0.09) |
| Ireland | 3.65 (2.2-5.83) | 8.13 (4.72-12.35) | 1.23 | 0.21 (0.12-0.33) | 0.35 (0.2-0.53) | 1.94 (1.22-2.67) |
| Israel | 5.66 (3.38-8.89) | 9.18 (5.55-14.02) | 0.62 | 0.23 (0.14-0.37) | 0.2 (0.12-0.31) | -0.67 (-1.18--0.16) |
| Italy | 183.49 (114.75-276.5) | 64.69 (39.99-98.39) | -0.65 | 0.64 (0.4-0.96) | 0.26 (0.16-0.4) | -2.67 (-2.84--2.49) |
| Jamaica | 1.98 (1.17-3.07) | 3.96 (2.1-6.7) | 1 | 0.17 (0.1-0.26) | 0.26 (0.14-0.44) | 0.97 (0.6-1.33) |
| Japan | 111.48 (65.01-180.14) | 58.01 (33.11-96.35) | -0.48 | 0.17 (0.1-0.28) | 0.11 (0.07-0.19) | -1.34 (-1.55--1.13) |
| Jordan | 1.59 (0.87-2.66) | 6.58 (3.56-10.91) | 3.14 | 0.09 (0.05-0.15) | 0.1 (0.05-0.16) | 0.04 (-0.23-0.31) |
| Kazakhstan | 18.31 (10.92-29.33) | 124.42 (69.86-206.31) | 5.8 | 0.22 (0.13-0.36) | 1.33 (0.75-2.21) | 5.86 (4.65-7.09) |
| Kenya | 25.39 (12.78-48.48) | 118.62 (66.84-211.02) | 3.67 | 0.25 (0.13-0.48) | 0.45 (0.26-0.81) | 2.31 (2.15-2.48) |
| Kiribati | 0.21 (0.12-0.36) | 0.4 (0.22-0.71) | 0.9 | 0.57 (0.32-0.97) | 0.64 (0.36-1.15) | -0.06 (-0.37-0.26) |
| Kuwait | 0.89 (0.51-1.45) | 2.98 (1.63-4.84) | 2.35 | 0.09 (0.05-0.14) | 0.1 (0.05-0.16) | 0.65 (-0.32-1.63) |
| Kyrgyzstan | 10.41 (6.4-16.49) | 34.06 (19.02-57.25) | 2.27 | 0.5 (0.31-0.79) | 0.99 (0.55-1.67) | 1.8 (1.03-2.58) |
| Lao People's Democratic Republic | 4.22 (2.44-7.45) | 7.03 (3.68-11.95) | 0.67 | 0.23 (0.13-0.4) | 0.18 (0.09-0.3) | -1.06 (-1.16--0.96) |
| Latvia | 3.72 (2.16-6.12) | 9.14 (4.87-14.7) | 1.46 | 0.29 (0.17-0.48) | 1.16 (0.62-1.86) | 3.83 (3.01-4.65) |
| Lebanon | 1.65 (0.76-3.17) | 2.85 (1.54-4.73) | 0.73 | 0.12 (0.05-0.22) | 0.09 (0.05-0.16) | -0.67 (-0.97--0.36) |
| Lesotho | 1.76 (0.77-3.59) | 6.58 (3.33-11.56) | 2.74 | 0.26 (0.12-0.54) | 0.65 (0.33-1.15) | 3.43 (2.91-3.96) |
| Liberia | 3.46 (1.94-5.6) | 12.2 (6.75-20.94) | 2.53 | 0.32 (0.18-0.52) | 0.44 (0.24-0.75) | 1.01 (0.54-1.47) |
| Libya | 2.94 (1.44-6.07) | 13.55 (6.98-26.25) | 3.61 | 0.15 (0.07-0.31) | 0.33 (0.17-0.64) | 3.45 (2.86-4.05) |
| Lithuania | 5.25 (2.96-8.56) | 14.2 (7.96-23.06) | 1.7 | 0.29 (0.16-0.47) | 1.23 (0.69-2) | 5.08 (3.84-6.33) |
| Luxembourg | 1.6 (0.92-2.58) | 1.11 (0.64-1.8) | -0.31 | 0.81 (0.46-1.3) | 0.35 (0.2-0.57) | -2.51 (-2.85--2.17) |
| Madagascar | 10.58 (6.44-16.75) | 31.08 (17.2-54.55) | 1.94 | 0.2 (0.12-0.32) | 0.22 (0.12-0.39) | 0.38 (0.31-0.45) |
| Malawi | 14.99 (9.11-23.54) | 40.04 (23.13-66.4) | 1.67 | 0.34 (0.21-0.53) | 0.41 (0.24-0.69) | 0.46 (0.27-0.66) |
| Malaysia | 7.42 (4.47-12.01) | 26.39 (14.91-43.86) | 2.56 | 0.08 (0.05-0.13) | 0.15 (0.08-0.25) | 1.32 (1.08-1.56) |
| Maldives | 0.1 (0.05-0.18) | 0.25 (0.13-0.43) | 1.5 | 0.11 (0.05-0.19) | 0.07 (0.04-0.13) | -1.34 (-1.64--1.04) |
| Mali | 8.9 (4.5-15.25) | 26.81 (13.7-44.74) | 2.01 | 0.25 (0.12-0.42) | 0.26 (0.13-0.43) | 0.51 (0.31-0.72) |
| Malta | 0.54 (0.3-0.88) | 0.64 (0.37-1.03) | 0.19 | 0.28 (0.16-0.46) | 0.33 (0.19-0.53) | 0.1 (-0.21-0.41) |
| Marshall Islands | 0.08 (0.05-0.12) | 0.16 (0.07-0.29) | 1 | 0.4 (0.23-0.62) | 0.53 (0.24-0.95) | 0.67 (0.55-0.79) |
| Mauritania | 2.86 (1.64-4.78) | 6.94 (3.86-12.22) | 1.43 | 0.31 (0.18-0.52) | 0.34 (0.19-0.59) | 0.19 (0.11-0.26) |
| Mauritius | 1.18 (0.69-1.89) | 1.72 (0.94-2.83) | 0.46 | 0.2 (0.11-0.31) | 0.27 (0.15-0.44) | -0.38 (-0.93-0.17) |
| Mexico | 629.94 (391.94-961.06) | 1634.38 (1022.43-2392.61) | 1.59 | 1.48 (0.92-2.26) | 2.39 (1.49-3.49) | 1.55 (1.37-1.74) |
| Micronesia (Federated States of) | 0.27 (0.14-0.45) | 0.35 (0.18-0.63) | 0.3 | 0.57 (0.3-0.96) | 0.65 (0.33-1.16) | 0.13 (-0.11-0.37) |
| Monaco | 0.11 (0.06-0.19) | 0.15 (0.08-0.25) | 0.36 | 0.81 (0.47-1.38) | 1.07 (0.57-1.78) | 1.06 (0.73-1.39) |
| Mongolia | 5.55 (3.28-8.94) | 12.09 (6.77-19.98) | 1.18 | 0.54 (0.32-0.87) | 0.72 (0.4-1.18) | 1.23 (0.94-1.52) |
| Montenegro | 0.59 (0.33-0.98) | 0.67 (0.37-1.17) | 0.14 | 0.18 (0.1-0.31) | 0.23 (0.13-0.4) | 0.91 (0.67-1.16) |
| Morocco | 11.44 (6.01-19.28) | 30.9 (15.83-58.18) | 1.7 | 0.09 (0.05-0.15) | 0.16 (0.08-0.3) | 1.72 (1.66-1.78) |
| Mozambique | 6.34 (3.66-10.53) | 21.75 (11.62-37.71) | 2.43 | 0.11 (0.06-0.18) | 0.15 (0.08-0.26) | 1.72 (1.5-1.95) |
| Myanmar | 54.81 (26.06-102.63) | 86.52 (41.68-149.64) | 0.58 | 0.27 (0.13-0.5) | 0.29 (0.14-0.51) | 0.2 (0.15-0.25) |
| Namibia | 1.62 (0.74-3.29) | 4.35 (2.16-8.36) | 1.69 | 0.25 (0.11-0.5) | 0.34 (0.17-0.65) | 0.75 (0.4-1.1) |
| Nauru | 0.04 (0.02-0.07) | 0.04 (0.01-0.07) | 0 | 0.71 (0.32-1.33) | 0.66 (0.25-1.26) | -0.71 (-0.89--0.53) |
| Nepal | 19.73 (11.24-33.81) | 56.22 (30.86-92.61) | 1.85 | 0.22 (0.13-0.38) | 0.34 (0.19-0.56) | 1.7 (1.46-1.95) |
| Netherlands | 20.66 (11.87-32.54) | 12.68 (7.35-19.35) | -0.39 | 0.25 (0.15-0.4) | 0.17 (0.1-0.26) | -1.89 (-2.39--1.4) |
| New Zealand | 2.14 (1.33-3.14) | 3.23 (2.09-4.71) | 0.51 | 0.12 (0.07-0.17) | 0.13 (0.09-0.19) | 0.66 (0.44-0.88) |
| Nicaragua | 9.11 (5.49-14.24) | 35.98 (20.38-59.03) | 2.95 | 0.53 (0.32-0.82) | 1 (0.57-1.64) | 2.28 (2.05-2.51) |
| Niger | 8.56 (4.66-15.84) | 21.96 (11.1-42.41) | 1.57 | 0.26 (0.14-0.47) | 0.21 (0.11-0.41) | -0.34 (-0.46--0.23) |
| Nigeria | 106.41 (57.1-182.4) | 258.62 (132.3-432.8) | 1.43 | 0.26 (0.14-0.44) | 0.24 (0.12-0.4) | -0.02 (-0.13-0.09) |
| Niue | 0 (0-0.01) | 0.01 (0-0.01) | Inf | 0.47 (0.26-0.85) | 0.65 (0.35-1.09) | 0.55 (0.33-0.77) |
| North Macedonia | 1.58 (0.92-2.6) | 1.97 (1.05-3.57) | 0.25 | 0.15 (0.09-0.25) | 0.18 (0.1-0.32) | 0.36 (0.04-0.69) |
| Northern Mariana Islands | 0.29 (0.16-0.51) | 0.24 (0.14-0.39) | -0.17 | 0.99 (0.54-1.77) | 1.02 (0.58-1.66) | 0.05 (-0.26-0.36) |
| Norway | 3.87 (2.35-5.91) | 3.5 (2.13-5.27) | -0.1 | 0.18 (0.11-0.27) | 0.14 (0.09-0.21) | -1.07 (-1.47--0.66) |
| Oman | 0.85 (0.43-1.52) | 3.89 (1.92-7.4) | 3.58 | 0.08 (0.04-0.15) | 0.13 (0.06-0.25) | 1.89 (1.72-2.07) |
| Pakistan | 125.17 (59.77-253.58) | 338.2 (194.09-540.41) | 1.7 | 0.25 (0.12-0.51) | 0.28 (0.16-0.44) | 0.14 (0.04-0.24) |
| Palau | 0.04 (0.02-0.08) | 0.09 (0.04-0.15) | 1.25 | 0.49 (0.23-0.93) | 0.96 (0.47-1.66) | 1.97 (1.74-2.21) |
| Palestine | 0.62 (0.32-1.17) | 1.31 (0.73-2.17) | 1.11 | 0.07 (0.04-0.13) | 0.05 (0.03-0.08) | -1.24 (-1.38--1.1) |
| Panama | 3.45 (2.07-5.4) | 8.66 (4.72-14.51) | 1.51 | 0.28 (0.17-0.44) | 0.4 (0.22-0.67) | 1.26 (1.12-1.4) |
| Papua New Guinea | 2.15 (1.04-3.82) | 5 (2.73-8.45) | 1.33 | 0.11 (0.05-0.19) | 0.09 (0.05-0.16) | -0.81 (-1.01--0.6) |
| Paraguay | 3.2 (1.89-5.01) | 10.13 (5.42-16.56) | 2.17 | 0.17 (0.1-0.27) | 0.26 (0.14-0.43) | 1.69 (1.57-1.81) |
| Peru | 94.26 (55.59-144.07) | 189.97 (108.12-302.56) | 1.02 | 0.88 (0.52-1.35) | 0.98 (0.56-1.57) | 0.1 (-0.22-0.42) |
| Philippines | 37.78 (23.54-60.05) | 80.51 (46.17-128.12) | 1.13 | 0.12 (0.08-0.19) | 0.13 (0.08-0.21) | 0.23 (0.15-0.32) |
| Poland | 36.82 (22.24-57.07) | 138.32 (84.11-212.77) | 2.76 | 0.19 (0.12-0.3) | 0.77 (0.47-1.19) | 3.49 (2.93-4.05) |
| Portugal | 48.99 (28.84-77.24) | 25.6 (14.59-41.15) | -0.48 | 0.98 (0.58-1.54) | 0.56 (0.32-0.9) | -2.23 (-2.8--1.66) |
| Puerto Rico | 30.14 (17.78-46.29) | 15.6 (9.22-23.51) | -0.48 | 1.63 (0.96-2.51) | 1.07 (0.63-1.61) | -2.17 (-2.6--1.74) |
| Qatar | 0.54 (0.3-0.89) | 4.52 (2.32-7.59) | 7.37 | 0.18 (0.1-0.31) | 0.21 (0.11-0.35) | -0.29 (-0.96-0.38) |
| Republic of Korea | 51.05 (27.17-86.19) | 21.17 (10.63-38.14) | -0.59 | 0.2 (0.11-0.33) | 0.09 (0.04-0.16) | -2.75 (-2.84--2.65) |
| Republic of Moldova | 31.19 (17.78-51.7) | 42.53 (24.33-70.75) | 0.36 | 1.42 (0.81-2.35) | 2.38 (1.36-3.95) | 1.53 (1.09-1.97) |
| Romania | 55.49 (32.61-90.02) | 79.99 (44.05-136.32) | 0.44 | 0.49 (0.29-0.79) | 0.96 (0.53-1.64) | 1.03 (0.47-1.6) |
| Russian Federation | 158.01 (96.67-246.21) | 1593.98 (944.34-2488.09) | 9.09 | 0.21 (0.13-0.33) | 2.36 (1.4-3.69) | 7.32 (6-8.65) |
| Rwanda | 10.52 (5.4-18.45) | 20.07 (10.25-35.63) | 0.91 | 0.33 (0.17-0.58) | 0.29 (0.15-0.52) | -1.51 (-1.97--1.04) |
| Saint Kitts and Nevis | 0.16 (0.1-0.24) | 0.24 (0.13-0.42) | 0.5 | 0.8 (0.48-1.21) | 0.77 (0.41-1.33) | -0.63 (-1.39-0.14) |
| Saint Lucia | 0.47 (0.29-0.74) | 0.94 (0.52-1.53) | 1 | 0.71 (0.44-1.11) | 1.01 (0.56-1.65) | 1.33 (0.97-1.7) |
| Saint Vincent and the Grenadines | 0.19 (0.11-0.29) | 0.52 (0.29-0.86) | 1.74 | 0.35 (0.21-0.55) | 0.92 (0.51-1.52) | 3.23 (2.85-3.61) |
| Samoa | 0.28 (0.15-0.49) | 0.43 (0.22-0.77) | 0.54 | 0.36 (0.19-0.62) | 0.43 (0.22-0.77) | 0.35 (0.22-0.48) |
| San Marino | 0.06 (0.04-0.1) | 0.07 (0.03-0.14) | 0.17 | 0.51 (0.29-0.83) | 0.5 (0.23-1.02) | 1.23 (0.7-1.77) |
| Sao Tome and Principe | 0.23 (0.12-0.4) | 0.7 (0.33-1.31) | 2.04 | 0.47 (0.25-0.8) | 0.62 (0.29-1.16) | 0.79 (0.52-1.07) |
| Saudi Arabia | 18.32 (9.17-36.42) | 139.05 (67.26-233.72) | 6.59 | 0.23 (0.11-0.45) | 0.55 (0.27-0.92) | 3.05 (2.87-3.24) |
| Senegal | 8.15 (4.52-13.55) | 20.4 (10.73-35.13) | 1.5 | 0.25 (0.14-0.41) | 0.26 (0.14-0.45) | 0.43 (0.2-0.66) |
| Serbia | 11.17 (6.2-18.09) | 9.53 (5.15-16.86) | -0.15 | 0.23 (0.13-0.38) | 0.22 (0.12-0.4) | -0.3 (-0.53--0.08) |
| Seychelles | 0.12 (0.07-0.19) | 0.26 (0.14-0.45) | 1.17 | 0.32 (0.18-0.52) | 0.49 (0.26-0.84) | 1.11 (0.77-1.45) |
| Sierra Leone | 4.25 (2.26-7.51) | 11.15 (6.13-18.41) | 1.62 | 0.22 (0.12-0.4) | 0.25 (0.14-0.41) | 0.54 (0.43-0.65) |
| Singapore | 1.54 (0.84-2.55) | 1.09 (0.59-1.84) | -0.29 | 0.08 (0.04-0.13) | 0.04 (0.02-0.06) | -2.97 (-3.2--2.73) |
| Slovakia | 14.72 (8.08-25) | 14.64 (8.18-25.55) | -0.01 | 0.55 (0.3-0.94) | 0.57 (0.32-0.99) | 0.17 (-0.06-0.41) |
| Slovenia | 5.01 (2.83-8.19) | 2.06 (1.11-3.44) | -0.59 | 0.49 (0.28-0.81) | 0.23 (0.13-0.39) | -3.04 (-3.72--2.36) |
| Solomon Islands | 0.49 (0.23-0.91) | 1.86 (1.05-3.28) | 2.8 | 0.32 (0.15-0.6) | 0.54 (0.31-0.95) | 1.72 (1.55-1.89) |
| Somalia | 14.39 (7.3-25.58) | 35.93 (17.69-67.98) | 1.5 | 0.41 (0.21-0.73) | 0.36 (0.18-0.68) | -1.06 (-1.29--0.83) |
| South Africa | 83.24 (54.25-126.67) | 177.46 (108.74-271.09) | 1.13 | 0.44 (0.29-0.67) | 0.57 (0.35-0.87) | 0.68 (0.12-1.25) |
| South Sudan | 7.1 (3.66-12.53) | 19.45 (10.51-34.98) | 1.74 | 0.26 (0.14-0.46) | 0.44 (0.24-0.79) | 1.76 (1.4-2.12) |
| Spain | 121.97 (73.34-189.68) | 65.05 (38.08-100.99) | -0.47 | 0.63 (0.38-0.98) | 0.33 (0.19-0.51) | -2.3 (-2.65--1.94) |
| Sri Lanka | 21.73 (12.46-35.98) | 14.33 (6.64-27.16) | -0.34 | 0.24 (0.14-0.39) | 0.13 (0.06-0.25) | -2.88 (-3.42--2.34) |
| Sudan | 10.34 (5.35-19.45) | 30.06 (14.23-59.38) | 1.91 | 0.11 (0.06-0.21) | 0.13 (0.06-0.27) | 0.84 (0.65-1.03) |
| Suriname | 1.51 (0.86-2.38) | 2.91 (1.61-4.74) | 0.93 | 0.76 (0.43-1.21) | 1.01 (0.56-1.65) | 1.04 (0.8-1.28) |
| Sweden | 7.28 (3.94-12.3) | 3.23 (1.63-5.66) | -0.56 | 0.17 (0.09-0.29) | 0.07 (0.04-0.13) | -2.24 (-2.49--1.99) |
| Switzerland | 14.54 (8.59-23.37) | 6.05 (3.58-9.61) | -0.58 | 0.4 (0.24-0.64) | 0.15 (0.09-0.24) | -2.91 (-3.34--2.48) |
| Syrian Arab Republic | 6.95 (3.95-11.59) | 11.92 (6.2-23.2) | 0.72 | 0.12 (0.07-0.21) | 0.17 (0.09-0.33) | 1.25 (0.71-1.79) |
| Taiwan (Province of China) | 17.46 (10.11-27.41) | 23.82 (12.92-40.3) | 0.36 | 0.16 (0.09-0.24) | 0.21 (0.11-0.35) | 0.98 (0.53-1.43) |
| Tajikistan | 7.56 (4.54-11.73) | 17.62 (9.72-30.48) | 1.33 | 0.31 (0.19-0.49) | 0.34 (0.19-0.59) | -0.51 (-0.86--0.17) |
| Thailand | 66.27 (38.63-110.07) | 215.7 (107.95-379.16) | 2.25 | 0.21 (0.12-0.35) | 0.68 (0.34-1.19) | 4 (3.64-4.37) |
| Timor-Leste | 0.44 (0.22-0.88) | 0.66 (0.32-1.24) | 0.5 | 0.12 (0.06-0.23) | 0.1 (0.05-0.18) | -0.9 (-1.32--0.47) |
| Togo | 3.41 (1.93-5.66) | 12.6 (6.71-22.92) | 2.7 | 0.21 (0.12-0.35) | 0.3 (0.16-0.55) | 1.47 (1.34-1.61) |
| Tokelau | 0 (0-0) | 0 (0-0.01) | NA | 0.35 (0.19-0.62) | 0.48 (0.27-0.8) | 0.36 (0.15-0.58) |
| Tonga | 0.26 (0.15-0.42) | 0.37 (0.19-0.65) | 0.42 | 0.59 (0.34-0.96) | 0.75 (0.39-1.32) | 0.61 (0.36-0.87) |
| Trinidad and Tobago | 4.08 (2.43-6.43) | 5.92 (3.15-9.74) | 0.45 | 0.66 (0.39-1.04) | 0.86 (0.46-1.41) | 0.87 (0.58-1.16) |
| Tunisia | 4.35 (2.38-7.34) | 12.33 (6.08-23.3) | 1.83 | 0.11 (0.06-0.18) | 0.2 (0.1-0.39) | 2.29 (2.21-2.37) |
| Turkey | 19.78 (11.05-33.44) | 27.4 (14.64-48.17) | 0.39 | 0.07 (0.04-0.12) | 0.06 (0.03-0.11) | -0.46 (-0.72--0.2) |
| Turkmenistan | 8.91 (5.46-14.09) | 82.52 (49.94-140.08) | 8.26 | 0.51 (0.31-0.8) | 3.08 (1.87-5.23) | 6.14 (5.84-6.44) |
| Tuvalu | 0.02 (0.01-0.04) | 0.03 (0.02-0.05) | 0.5 | 0.5 (0.28-0.83) | 0.49 (0.27-0.84) | -0.34 (-0.52--0.17) |
| Uganda | 11.88 (6.59-20.03) | 43.49 (23.59-73.53) | 2.66 | 0.16 (0.09-0.27) | 0.22 (0.12-0.37) | 0.52 (0.34-0.7) |
| Ukraine | 85 (50.6-135.26) | 430.88 (232.51-727.69) | 4.07 | 0.34 (0.2-0.54) | 2.13 (1.15-3.6) | 5.15 (3.88-6.44) |
| United Arab Emirates | 1.28 (0.69-2.17) | 11.96 (6.23-20.13) | 8.34 | 0.11 (0.06-0.18) | 0.17 (0.09-0.29) | 1.44 (1.09-1.78) |
| United Kingdom | 127.5 (80.24-188.92) | 326.79 (214.79-450.07) | 1.56 | 0.45 (0.28-0.66) | 1.08 (0.71-1.48) | 2.76 (1.82-3.7) |
| United Republic of Tanzania | 23.07 (13.61-39.35) | 76.52 (41.4-130.35) | 2.32 | 0.2 (0.12-0.35) | 0.27 (0.15-0.46) | 1.16 (1-1.31) |
| United States of America | 468.59 (288.24-727.5) | 601.48 (363.51-954.1) | 0.28 | 0.35 (0.21-0.54) | 0.4 (0.24-0.63) | 0.28 (0.12-0.44) |
| United States Virgin Islands | 0.72 (0.42-1.15) | 0.6 (0.36-0.95) | -0.17 | 1.31 (0.76-2.09) | 1.78 (1.05-2.82) | 1.46 (1.28-1.65) |
| Uruguay | 4.15 (2.38-6.85) | 4.09 (2.4-6.56) | -0.01 | 0.28 (0.16-0.46) | 0.25 (0.15-0.4) | -0.2 (-0.39--0.01) |
| Uzbekistan | 43.52 (27.52-67.66) | 197 (108.93-311.2) | 3.53 | 0.44 (0.28-0.69) | 1.1 (0.61-1.74) | 2.26 (1.82-2.69) |
| Vanuatu | 0.33 (0.15-0.63) | 0.87 (0.45-1.59) | 1.64 | 0.46 (0.21-0.89) | 0.56 (0.29-1.02) | 0.33 (0.25-0.41) |
| Venezuela (Bolivarian Republic of) | 47.84 (27.96-73.94) | 100.38 (51.96-175.08) | 1.1 | 0.5 (0.29-0.77) | 0.76 (0.4-1.33) | 0.97 (0.59-1.36) |
| Viet Nam | 34.17 (19.58-60.15) | 75.68 (37.67-134.05) | 1.21 | 0.1 (0.06-0.18) | 0.15 (0.07-0.26) | 1.19 (1.08-1.31) |
| Yemen | 4.85 (2.16-9.65) | 11.19 (5.99-20.48) | 1.31 | 0.09 (0.04-0.18) | 0.07 (0.04-0.12) | -1.23 (-1.39--1.07) |
| Zambia | 12.48 (7.39-19.58) | 37.61 (17.54-68.43) | 2.01 | 0.35 (0.21-0.55) | 0.39 (0.18-0.71) | -0.01 (-0.13-0.1) |
| Zimbabwe | 6.96 (4.04-11.4) | 27.34 (11.25-55.06) | 2.93 | 0.15 (0.09-0.25) | 0.35 (0.15-0.71) | 2.96 (2.28-3.64) |
