# Supplementary material for: Global, regional and national burden of Metabolic dysfunction-associated steatotic liver disease in adolescents and adults aged 15–49 years from 1990 to 2021: results from the 2021 Global Burden of Disease study
Source: Front Med (Lausanne). 2025 Jun 25;12:1568211. doi: 10.3389/fmed.2025.1568211 (PMC12237898; doi:10.3389/fmed.2025.1568211)
Supplement: Supplementary file 1 [file Supplementary_file_1.ZIP › Supplementary Table 8.docx]

**Supplementary Table 8** The incidence cases and rates for MASLD among the adolescents and adults aged 15-49 years in age patterns from 1990 to 2021

| **location** | **Age (year)** | **Prevalence cases** | | | **Prevalence rates** | | |
| --- | --- | --- | --- | --- | --- | --- | --- |
|  |  | **1990 thousand**  **(95%UI)** | **2021** **thousand**  **(95%UI)** | **percentage**  **Change**  **(100%)** | **1990**  **Per 100,000**  **(95%UI)** | **2021**  **Per 100,000**  **(95%UI)** | **EAPC**  **(95% CI)** |
| Global | 15-19 years | 39.5 (18.2-69.54) | 46.12 (21.92-80.06) | 0.17 (0.2-0.15) | 7.6 (3.5-13.39) | 7.39 (3.51-12.83) | -0.27 (-0.34--0.21) |
| Global | 15-49 years | 565.74 (436.63-725.49) | 906.53 (709.48-1124.51) | 0.6 (0.62-0.55) | 20.87 (16.11-26.77) | 22.96 (17.97-28.48) | 0.2 (0.16-0.25) |
| Global | 20-24 years | 44.83 (24.22-73.62) | 53.3 (29.93-85.55) | 0.19 (0.24-0.16) | 9.11 (4.92-14.96) | 8.93 (5.01-14.33) | -0.22 (-0.27--0.17) |
| Global | 25-29 years | 50.54 (26.75-85.5) | 64.64 (34.36-106.14) | 0.28 (0.28-0.24) | 11.42 (6.04-19.32) | 10.99 (5.84-18.04) | -0.21 (-0.25--0.17) |
| Global | 30-34 years | 62.79 (38.98-95.27) | 94.66 (60.26-142.06) | 0.51 (0.55-0.49) | 16.29 (10.11-24.72) | 15.66 (9.97-23.5) | -0.18 (-0.24--0.12) |
| Global | 35-39 years | 81.15 (45.06-131.83) | 128.74 (73.98-206.18) | 0.59 (0.64-0.56) | 23.04 (12.79-37.43) | 22.95 (13.19-36.76) | -0.12 (-0.19--0.05) |
| Global | 40-44 years | 119.14 (80.77-165.11) | 200.13 (139.72-268.42) | 0.68 (0.73-0.63) | 41.59 (28.2-57.63) | 40.01 (27.93-53.66) | -0.24 (-0.3--0.18) |
| Global | 45-49 years | 167.78 (111.78-241.49) | 318.92 (224.57-438.75) | 0.9 (1.01-0.82) | 72.26 (48.14-104) | 67.35 (47.43-92.66) | -0.3 (-0.36--0.25) |
| Low SDI | 15-19 years | 4.23 (1.98-7.46) | 9.88 (4.86-16.98) | 1.34 (1.45-1.28) | 8.35 (3.91-14.72) | 7.97 (3.92-13.7) | -0.25 (-0.32--0.18) |
| Low SDI | 15-49 years | 48.96 (37.48-63.69) | 111.01 (86.63-141.53) | 1.27 (1.31-1.22) | 22.15 (16.96-28.81) | 20.47 (15.97-26.09) | -0.3 (-0.37--0.24) |
| Low SDI | 20-24 years | 4.34 (2.38-7.08) | 10.12 (5.86-16.03) | 1.33 (1.46-1.26) | 10.19 (5.58-16.61) | 9.71 (5.62-15.37) | -0.25 (-0.3--0.2) |
| Low SDI | 25-29 years | 4.62 (2.43-7.69) | 10.39 (5.69-16.94) | 1.25 (1.34-1.2) | 12.89 (6.79-21.48) | 12.06 (6.6-19.66) | -0.31 (-0.37--0.25) |
| Low SDI | 30-34 years | 5.58 (3.48-8.49) | 12.51 (7.98-18.71) | 1.24 (1.29-1.2) | 18.79 (11.71-28.58) | 17.28 (11.03-25.84) | -0.34 (-0.4--0.28) |
| Low SDI | 35-39 years | 7.04 (3.81-11.54) | 15.77 (9.07-25.37) | 1.24 (1.38-1.2) | 27.59 (14.95-45.23) | 25.32 (14.56-40.74) | -0.33 (-0.37--0.29) |
| Low SDI | 40-44 years | 9.58 (6.46-13.26) | 22.38 (15.31-30.32) | 1.34 (1.37-1.29) | 48.06 (32.41-66.53) | 43.36 (29.66-58.73) | -0.37 (-0.41--0.33) |
| Low SDI | 45-49 years | 13.58 (9.36-19.44) | 29.96 (21.04-40.5) | 1.21 (1.25-1.08) | 80.81 (55.68-115.7) | 71.86 (50.46-97.12) | -0.44 (-0.49--0.39) |
| Low-middle SDI | 15-19 years | 8.58 (3.84-15.28) | 13.74 (6.54-23.81) | 0.6 (0.7-0.56) | 7.22 (3.23-12.85) | 7.44 (3.54-12.9) | -0.01 (-0.08-0.06) |
| Low-middle SDI | 15-49 years | 108.17 (82.78-138.58) | 221.4 (173.95-278.93) | 1.05 (1.1-1.01) | 19.63 (15.02-25.15) | 21.79 (17.12-27.45) | 0.33 (0.29-0.38) |
| Low-middle SDI | 20-24 years | 9.24 (4.95-15.21) | 16.03 (9.15-25.41) | 0.73 (0.85-0.67) | 8.86 (4.75-14.58) | 9.17 (5.23-14.54) | 0.05 (0-0.1) |
| Low-middle SDI | 25-29 years | 10.06 (5.29-16.96) | 18.51 (10.17-29.99) | 0.84 (0.92-0.77) | 11.22 (5.9-18.93) | 11.43 (6.28-18.53) | 0.03 (-0.04-0.1) |
| Low-middle SDI | 30-34 years | 12.33 (7.63-18.65) | 24.68 (16.01-36.9) | 1 (1.1-0.98) | 16.33 (10.1-24.7) | 16.7 (10.83-24.96) | 0.05 (-0.02-0.12) |
| Low-middle SDI | 35-39 years | 15.66 (8.65-25.33) | 33.28 (19.24-53.09) | 1.13 (1.22-1.1) | 24.07 (13.29-38.92) | 24.94 (14.42-39.78) | 0.09 (0.03-0.15) |
| Low-middle SDI | 40-44 years | 21.88 (14.73-30.59) | 48.33 (33.56-65.02) | 1.21 (1.28-1.13) | 41.23 (27.76-57.65) | 41.93 (29.11-56.41) | 0.04 (0.01-0.07) |
| Low-middle SDI | 45-49 years | 30.42 (20.3-44.12) | 66.83 (47.24-90.71) | 1.2 (1.33-1.06) | 68.16 (45.48-98.87) | 67.86 (47.97-92.1) | 0.01 (-0.01-0.03) |
| Middle SDI | 15-19 years | 14.46 (6.72-25.24) | 13.56 (6.39-23.41) | -0.06 (-0.05--0.07) | 7.72 (3.59-13.47) | 7.44 (3.5-12.84) | -0.33 (-0.4--0.27) |
| Middle SDI | 15-49 years | 169.1 (129.05-218.74) | 297.05 (232.84-371.35) | 0.76 (0.8-0.7) | 18.57 (14.17-24.02) | 23.67 (18.55-29.59) | 0.73 (0.69-0.76) |
| Middle SDI | 20-24 years | 16.1 (8.66-26.37) | 15.75 (8.71-25.35) | -0.02 (0.01--0.04) | 9.03 (4.85-14.79) | 8.89 (4.92-14.3) | -0.24 (-0.3--0.18) |
| Middle SDI | 25-29 years | 16.69 (8.95-28.13) | 19.85 (10.51-32.79) | 0.19 (0.17-0.17) | 11.06 (5.93-18.63) | 10.8 (5.72-17.84) | -0.2 (-0.25--0.14) |
| Middle SDI | 30-34 years | 18.99 (11.72-28.8) | 30.31 (19.06-45.71) | 0.6 (0.63-0.59) | 15.49 (9.56-23.5) | 15.19 (9.56-22.91) | -0.15 (-0.22--0.09) |
| Middle SDI | 35-39 years | 23.99 (13.46-39.08) | 40.86 (23.33-66.01) | 0.7 (0.73-0.69) | 21.14 (11.86-34.44) | 22.13 (12.64-35.75) | -0.03 (-0.11-0.05) |
| Middle SDI | 40-44 years | 33.06 (22.21-46.19) | 65.34 (45.65-88.18) | 0.98 (1.06-0.91) | 37.74 (25.35-52.74) | 39.62 (27.68-53.47) | 0.04 (-0.01-0.08) |
| Middle SDI | 45-49 years | 45.81 (29.64-66.65) | 111.39 (78.63-153.2) | 1.43 (1.65-1.3) | 65.08 (42.11-94.69) | 68.48 (48.34-94.19) | 0.13 (0.07-0.18) |
| High-middle SDI | 15-19 years | 7.93 (3.68-13.82) | 5.42 (2.51-9.43) | -0.32 (-0.32--0.32) | 8.21 (3.81-14.32) | 7.48 (3.46-13.01) | -0.52 (-0.59--0.46) |
| High-middle SDI | 15-49 years | 135.51 (105.8-172.14) | 169.54 (133.96-213.61) | 0.25 (0.27-0.24) | 24.01 (18.74-30.5) | 26.93 (21.28-33.93) | 0.2 (0.09-0.3) |
| High-middle SDI | 20-24 years | 9.7 (5.36-15.6) | 6.79 (3.78-10.97) | -0.3 (-0.29--0.3) | 9.94 (5.49-15.99) | 9.05 (5.04-14.63) | -0.51 (-0.59--0.42) |
| High-middle SDI | 25-29 years | 11.76 (6.54-19.2) | 9.43 (5.06-15.6) | -0.2 (-0.23--0.19) | 12.66 (7.04-20.66) | 11.14 (5.98-18.42) | -0.47 (-0.53--0.4) |
| High-middle SDI | 30-34 years | 15.24 (9.89-22.89) | 16.82 (10.79-25.23) | 0.1 (0.09-0.1) | 17.87 (11.6-26.84) | 15.77 (10.11-23.65) | -0.42 (-0.48--0.37) |
| High-middle SDI | 35-39 years | 19.87 (11.4-32.17) | 23.09 (13.21-37.02) | 0.16 (0.16-0.15) | 24.76 (14.21-40.1) | 22.76 (13.02-36.49) | -0.4 (-0.48--0.32) |
| High-middle SDI | 40-44 years | 29.37 (20.08-40.01) | 38.28 (26.96-51.49) | 0.3 (0.34-0.29) | 47.02 (32.14-64.04) | 41.43 (29.18-55.72) | -0.62 (-0.73--0.51) |
| High-middle SDI | 45-49 years | 41.64 (28.01-60.01) | 69.71 (48.84-96.32) | 0.67 (0.74-0.61) | 84.29 (56.69-121.46) | 71.93 (50.4-99.39) | -0.65 (-0.79--0.52) |
| High SDI | 15-19 years | 4.27 (1.83-7.8) | 3.5 (1.51-6.29) | -0.18 (-0.17--0.19) | 6.51 (2.79-11.91) | 5.81 (2.51-10.46) | -0.57 (-0.64--0.51) |
| High SDI | 15-49 years | 103.39 (79.39-135.1) | 106.82 (82.87-138.38) | 0.03 (0.04-0.02) | 22.43 (17.23-29.32) | 21.27 (16.5-27.55) | -0.42 (-0.52--0.32) |
| High SDI | 20-24 years | 5.41 (2.73-9.06) | 4.58 (2.38-7.77) | -0.15 (-0.13--0.14) | 7.85 (3.97-13.16) | 7 (3.64-11.88) | -0.48 (-0.52--0.43) |
| High SDI | 25-29 years | 7.36 (3.68-13.04) | 6.41 (3.2-11.14) | -0.13 (-0.13--0.15) | 10.1 (5.05-17.89) | 8.98 (4.48-15.61) | -0.45 (-0.5--0.4) |
| High SDI | 30-34 years | 10.59 (6.38-16.44) | 10.27 (6.33-15.56) | -0.03 (-0.01--0.05) | 14.7 (8.85-22.82) | 13.23 (8.15-20.05) | -0.43 (-0.51--0.35) |
| High SDI | 35-39 years | 14.51 (7.61-23.69) | 15.65 (8.77-24.97) | 0.08 (0.15-0.05) | 21.45 (11.25-35.02) | 19.9 (11.14-31.75) | -0.38 (-0.48--0.29) |
| High SDI | 40-44 years | 25.12 (17.13-35.09) | 25.64 (17.83-35.27) | 0.02 (0.04-0.01) | 39.77 (27.13-55.57) | 33.91 (23.58-46.64) | -0.69 (-0.79--0.59) |
| High SDI | 45-49 years | 36.14 (23.82-51.58) | 40.77 (28.13-56.78) | 0.13 (0.18-0.1) | 71.23 (46.96-101.65) | 55.57 (38.35-77.4) | -0.98 (-1.08--0.88) |
