# Supplementary material for: Global, regional and national burden of Metabolic dysfunction-associated steatotic liver disease in adolescents and adults aged 15–49 years from 1990 to 2021: results from the 2021 Global Burden of Disease study
Source: Front Med (Lausanne). 2025 Jun 25;12:1568211. doi: 10.3389/fmed.2025.1568211 (PMC12237898; doi:10.3389/fmed.2025.1568211)
Supplement: Supplementary file 1 [file Supplementary_file_1.ZIP › Supplementary Table 9.docx]

**Supplementary Table 9** The DALY cases and rates for MASLD in the adolescents and adults aged 15-49 years in age patterns from 1990 to 2021

| **location** | **Age (year)** | **Prevalence cases** | | | **Prevalence rates** | | |
| --- | --- | --- | --- | --- | --- | --- | --- |
|  |  | **1990**  **(95%UI)** | **2021**  **(95%UI)** | **percentage**  **Change**  **(100%)** | **1990**  **Per 100,000**  **(95%UI)** | **2021**  **Per 100,000**  **(95%UI)** | **EAPC**  **(95% CI)** |
| Global | 15-19 years | 9.14 (3.2-18.37) | 10.81 (4.11-21.94) | 0.18 (0.28-0.19) | 1.76 (0.62-3.54) | 1.73 (0.66-3.52) | -0.09 (-0.19-0) |
| Global | 15-49 years | 398.85 (260.59-610.81) | 750.63 (475.83-1124.35) | 0.88 (0.83-0.84) | 14.72 (9.61-22.54) | 19.01 (12.05-28.47) | 0.77 (0.6-0.93) |
| Global | 20-24 years | 17.28 (8.54-30.26) | 24.62 (12.56-42.59) | 0.42 (0.47-0.41) | 3.51 (1.74-6.15) | 4.12 (2.1-7.13) | 0.43 (0.24-0.62) |
| Global | 25-29 years | 30.7 (14.23-56.43) | 48.2 (23-86.98) | 0.57 (0.62-0.54) | 6.94 (3.21-12.75) | 8.19 (3.91-14.78) | 0.75 (0.43-1.07) |
| Global | 30-34 years | 48.26 (26.6-79.7) | 86.36 (47.11-140.09) | 0.79 (0.77-0.76) | 12.52 (6.9-20.68) | 14.29 (7.79-23.18) | 0.75 (0.5-1) |
| Global | 35-39 years | 69.58 (30.47-117.27) | 127.62 (55.21-219.67) | 0.83 (0.81-0.87) | 19.75 (8.65-33.29) | 22.75 (9.84-39.17) | 0.51 (0.44-0.59) |
| Global | 40-44 years | 96.31 (52.24-160.05) | 189.57 (101.17-315.37) | 0.97 (0.94-0.97) | 33.62 (18.24-55.87) | 37.9 (20.22-63.04) | 0.15 (0-0.3) |
| Global | 45-49 years | 127.57 (66.91-228.77) | 263.45 (142.42-454.7) | 1.07 (1.13-0.99) | 54.94 (28.82-98.52) | 55.64 (30.08-96.03) | -0.07 (-0.3-0.15) |
| Low SDI | 15-19 years | 1.29 (0.44-2.75) | 2.87 (0.99-6.08) | 1.22 (1.25-1.21) | 2.56 (0.86-5.43) | 2.32 (0.8-4.91) | -0.31 (-0.43--0.19) |
| Low SDI | 15-49 years | 27.77 (17.77-43.06) | 63.48 (40.25-95.97) | 1.29 (1.27-1.23) | 12.56 (8.04-19.48) | 11.7 (7.42-17.69) | -0.27 (-0.33--0.21) |
| Low SDI | 20-24 years | 1.96 (0.89-3.5) | 5.08 (2.49-9.11) | 1.59 (1.8-1.6) | 4.59 (2.08-8.2) | 4.87 (2.38-8.74) | 0.25 (0.1-0.4) |
| Low SDI | 25-29 years | 2.62 (1.1-5.16) | 6.59 (2.81-12.48) | 1.52 (1.55-1.42) | 7.32 (3.08-14.4) | 7.65 (3.26-14.49) | 0.18 (0.05-0.3) |
| Low SDI | 30-34 years | 3.29 (1.72-5.62) | 8.04 (4.17-13.49) | 1.44 (1.42-1.4) | 11.07 (5.78-18.91) | 11.1 (5.76-18.63) | -0.04 (-0.12-0.05) |
| Low SDI | 35-39 years | 4.14 (1.72-7.35) | 9.38 (3.87-16.64) | 1.27 (1.25-1.26) | 16.23 (6.76-28.81) | 15.06 (6.21-26.72) | -0.21 (-0.3--0.12) |
| Low SDI | 40-44 years | 6.31 (3.26-10.84) | 14.55 (7.48-24.97) | 1.31 (1.29-1.3) | 31.67 (16.34-54.4) | 28.19 (14.49-48.37) | -0.52 (-0.6--0.45) |
| Low SDI | 45-49 years | 8.16 (4.09-15.15) | 16.97 (8.52-31.45) | 1.08 (1.08-1.08) | 48.56 (24.34-90.14) | 40.7 (20.44-75.44) | -0.69 (-0.79--0.59) |
| Low-middle SDI | 15-19 years | 2.77 (0.93-5.62) | 4.13 (1.48-8.64) | 0.49 (0.59-0.54) | 2.33 (0.78-4.73) | 2.24 (0.8-4.68) | -0.11 (-0.32-0.1) |
| Low-middle SDI | 15-49 years | 79.03 (51.93-119.36) | 169.27 (103.17-262.03) | 1.14 (0.99-1.2) | 14.34 (9.42-21.66) | 16.66 (10.15-25.78) | 0.56 (0.46-0.66) |
| Low-middle SDI | 20-24 years | 5.2 (2.46-9.53) | 9.21 (4.6-16.46) | 0.77 (0.87-0.73) | 4.99 (2.36-9.14) | 5.27 (2.63-9.42) | 0.1 (-0.1-0.31) |
| Low-middle SDI | 25-29 years | 8.09 (3.62-15.43) | 15.24 (7.19-28.24) | 0.88 (0.99-0.83) | 9.02 (4.04-17.22) | 9.41 (4.44-17.44) | 0.26 (0.07-0.46) |
| Low-middle SDI | 30-34 years | 10.33 (5.57-17.17) | 21.53 (11.31-35.79) | 1.08 (1.03-1.08) | 13.68 (7.38-22.74) | 14.56 (7.65-24.21) | 0.27 (0.14-0.4) |
| Low-middle SDI | 35-39 years | 13.22 (5.77-23.36) | 27.68 (11.37-49.39) | 1.09 (0.97-1.11) | 20.32 (8.87-35.89) | 20.75 (8.52-37.02) | 0.21 (0.09-0.32) |
| Low-middle SDI | 40-44 years | 16.97 (9.13-28.76) | 39.59 (19.84-68.08) | 1.33 (1.17-1.37) | 31.98 (17.2-54.19) | 34.35 (17.21-59.06) | 0.25 (0.21-0.29) |
| Low-middle SDI | 45-49 years | 22.45 (11.18-42.62) | 51.89 (26.6-92.18) | 1.31 (1.38-1.16) | 50.31 (25.05-95.51) | 52.69 (27.01-93.59) | 0.26 (0.13-0.38) |
| Middle SDI | 15-19 years | 3.66 (1.32-7.25) | 3 (1.21-5.95) | -0.18 (-0.08--0.18) | 1.96 (0.7-3.87) | 1.65 (0.66-3.26) | -0.64 (-0.76--0.53) |
| Middle SDI | 15-49 years | 136.83 (91.53-205.53) | 264.84 (167.62-391.68) | 0.94 (0.83-0.91) | 15.03 (10.05-22.57) | 21.1 (13.36-31.21) | 1.02 (0.95-1.09) |
| Middle SDI | 20-24 years | 6.99 (3.57-12.03) | 7.83 (4-13.16) | 0.12 (0.12-0.09) | 3.92 (2-6.75) | 4.42 (2.25-7.42) | 0.23 (0.06-0.4) |
| Middle SDI | 25-29 years | 12.36 (5.69-22.31) | 17.63 (8.58-31.47) | 0.43 (0.51-0.41) | 8.19 (3.77-14.78) | 9.59 (4.67-17.12) | 0.45 (0.28-0.62) |
| Middle SDI | 30-34 years | 18.01 (9.94-29.46) | 30.53 (16.88-49.08) | 0.7 (0.7-0.67) | 14.7 (8.11-24.04) | 15.3 (8.46-24.6) | 0.33 (0.18-0.48) |
| Middle SDI | 35-39 years | 24.43 (10.75-42.16) | 44.38 (19.28-75.09) | 0.82 (0.79-0.78) | 21.54 (9.47-37.16) | 24.04 (10.44-40.67) | 0.35 (0.21-0.49) |
| Middle SDI | 40-44 years | 31.3 (17.08-51.05) | 66 (36.48-106.35) | 1.11 (1.14-1.08) | 35.74 (19.51-58.29) | 40.02 (22.12-64.48) | 0.19 (-0.01-0.39) |
| Middle SDI | 45-49 years | 40.07 (20.59-71.93) | 95.47 (51.89-161.28) | 1.38 (1.52-1.24) | 56.93 (29.25-102.19) | 58.7 (31.9-99.15) | -0.06 (-0.22-0.1) |
| High-middle SDI | 15-19 years | 1.03 (0.37-1.99) | 0.56 (0.24-1.06) | -0.46 (-0.35--0.47) | 1.07 (0.38-2.06) | 0.77 (0.33-1.47) | -1.23 (-1.36--1.11) |
| High-middle SDI | 15-49 years | 69.85 (44.1-106.78) | 157.56 (95.87-244.2) | 1.26 (1.17-1.29) | 12.38 (7.81-18.92) | 25.03 (15.23-38.79) | 2.25 (1.75-2.76) |
| High-middle SDI | 20-24 years | 2.07 (1.13-3.52) | 1.55 (0.82-2.63) | -0.25 (-0.27--0.25) | 2.13 (1.16-3.6) | 2.06 (1.09-3.5) | -0.09 (-0.73-0.56) |
| High-middle SDI | 25-29 years | 4.18 (1.97-7.67) | 5.42 (2.59-9.73) | 0.3 (0.31-0.27) | 4.5 (2.12-8.25) | 6.41 (3.06-11.49) | 2.13 (0.89-3.4) |
| High-middle SDI | 30-34 years | 7.82 (4.36-12.76) | 17.65 (9.64-28.15) | 1.26 (1.21-1.21) | 9.18 (5.12-14.97) | 16.54 (9.03-26.38) | 2.94 (2.01-3.89) |
| High-middle SDI | 35-39 years | 12.19 (5.2-20.79) | 30.98 (13.69-52.15) | 1.54 (1.63-1.51) | 15.19 (6.48-25.92) | 30.54 (13.5-51.41) | 2.58 (2.21-2.95) |
| High-middle SDI | 40-44 years | 17.95 (9.5-29.47) | 44.09 (23.82-72.23) | 1.46 (1.51-1.45) | 28.74 (15.2-47.17) | 47.71 (25.78-78.17) | 1.28 (0.89-1.68) |
| High-middle SDI | 45-49 years | 24.61 (12.84-43.63) | 57.31 (29.97-100.98) | 1.33 (1.33-1.31) | 49.81 (25.99-88.31) | 59.14 (30.92-104.21) | 0.3 (-0.23-0.84) |
| High SDI | 15-19 years | 0.38 (0.15-0.74) | 0.24 (0.11-0.44) | -0.37 (-0.27--0.41) | 0.58 (0.23-1.13) | 0.4 (0.18-0.74) | -1.31 (-1.36--1.26) |
| High SDI | 15-49 years | 84.83 (52.19-130.1) | 94.7 (59.17-141.45) | 0.12 (0.13-0.09) | 18.41 (11.32-28.23) | 18.86 (11.78-28.16) | -0.2 (-0.45-0.05) |
| High SDI | 20-24 years | 1.03 (0.54-1.76) | 0.92 (0.49-1.59) | -0.11 (-0.09--0.1) | 1.5 (0.78-2.56) | 1.41 (0.75-2.44) | 0.04 (-0.05-0.14) |
| High SDI | 25-29 years | 3.42 (1.62-6.23) | 3.28 (1.61-5.7) | -0.04 (-0.01--0.09) | 4.69 (2.22-8.54) | 4.6 (2.26-7.98) | 0.4 (0.09-0.72) |
| High SDI | 30-34 years | 8.75 (4.91-13.92) | 8.55 (4.85-13.38) | -0.02 (-0.01--0.04) | 12.14 (6.82-19.32) | 11.01 (6.25-17.24) | -0.32 (-0.67-0.03) |
| High SDI | 35-39 years | 15.51 (6.82-26.27) | 15.08 (6.91-24.92) | -0.03 (0.01--0.05) | 22.93 (10.08-38.84) | 19.17 (8.78-31.68) | -0.95 (-1.17--0.72) |
| High SDI | 40-44 years | 23.64 (12.6-38.78) | 25.14 (13.88-39.99) | 0.06 (0.1-0.03) | 37.44 (19.95-61.41) | 33.24 (18.36-52.88) | -0.85 (-1.12--0.58) |
| High SDI | 45-49 years | 32.1 (16.77-55.95) | 41.49 (22.98-69.83) | 0.29 (0.37-0.25) | 63.26 (33.05-110.28) | 56.55 (31.32-95.18) | -0.35 (-0.66--0.03) |
