# Supplementary material for: Global, regional and national burden of Metabolic dysfunction-associated steatotic liver disease in adolescents and adults aged 15–49 years from 1990 to 2021: results from the 2021 Global Burden of Disease study
Source: Front Med (Lausanne). 2025 Jun 25;12:1568211. doi: 10.3389/fmed.2025.1568211 (PMC12237898; doi:10.3389/fmed.2025.1568211)
Supplement: Supplementary file 1 [file Supplementary_file_1.ZIP › Supplementary Table 10.docx]

**Supplementary Table 10** The death cases and rates for MASLD among the adolescents and adults aged 15-49 years in age patterns from 1990 to 2021

| **location** | **Age (year)** | **Prevalence cases** | | | **Prevalence rates** | | |
| --- | --- | --- | --- | --- | --- | --- | --- |
|  |  | **1990 thousand**  **(95%UI)** | **2021 thousand**  **(95%UI)** | **percentage**  **Change**  **(100%)** | **1990 per**  **(95%UI)** | **2021 per**  **(95%UI)** | **EAPC**  **(95% CI)** |
| Global | 15-19 years | 121.69 (42.56-244.53) | 143.02 (53.76-290.55) | 0.18 (0.26-0.19) | 0.02 (0.01-0.05) | 0.02 (0.01-0.05) | -0.11 (-0.21--0.01) |
| Global | 15-49 years | 7919.77 (5125.49-12455.41) | 15107.88 (9519.61-22599.46) | 0.91 (0.86-0.81) | 0.29 (0.19-0.46) | 0.38 (0.24-0.57) | 0.8 (0.63-0.97) |
| Global | 20-24 years | 249.63 (122.65-438.59) | 354.61 (180.4-613.92) | 0.42 (0.47-0.4) | 0.05 (0.02-0.09) | 0.06 (0.03-0.1) | 0.42 (0.23-0.61) |
| Global | 25-29 years | 481.95 (224.07-884.45) | 755.77 (360.42-1360.44) | 0.57 (0.61-0.54) | 0.11 (0.05-0.2) | 0.13 (0.06-0.23) | 0.75 (0.42-1.08) |
| Global | 30-34 years | 825.28 (454.88-1360.14) | 1474.72 (805.72-2381.06) | 0.79 (0.77-0.75) | 0.21 (0.12-0.35) | 0.24 (0.13-0.39) | 0.75 (0.49-1) |
| Global | 35-39 years | 1303.86 (569.04-2204.21) | 2389.62 (1034.93-4125.86) | 0.83 (0.82-0.87) | 0.37 (0.16-0.63) | 0.43 (0.18-0.74) | 0.51 (0.43-0.59) |
| Global | 40-44 years | 1992.4 (1080.29-3311.89) | 3920.67 (2081.96-6515.01) | 0.97 (0.93-0.97) | 0.7 (0.38-1.16) | 0.78 (0.42-1.3) | 0.15 (-0.01-0.3) |
| Global | 45-49 years | 2944.95 (1545.24-5278.66) | 6069.48 (3280.3-10493.93) | 1.06 (1.12-0.99) | 1.27 (0.67-2.27) | 1.28 (0.69-2.22) | -0.07 (-0.29-0.15) |
| Low SDI | 15-19 years | 17.47 (5.85-37.11) | 38.59 (13.26-82.38) | 1.21 (1.27-1.22) | 0.03 (0.01-0.07) | 0.03 (0.01-0.07) | -0.32 (-0.44--0.2) |
| Low SDI | 15-49 years | 541.89 (343.85-856.89) | 1224.21 (755.83-1868.18) | 1.26 (1.2-1.18) | 0.25 (0.16-0.39) | 0.23 (0.14-0.34) | -0.31 (-0.37--0.26) |
| Low SDI | 20-24 years | 28.51 (12.9-50.75) | 73.96 (36.05-132.84) | 1.59 (1.79-1.62) | 0.07 (0.03-0.12) | 0.07 (0.03-0.13) | 0.25 (0.09-0.4) |
| Low SDI | 25-29 years | 41.34 (17.41-81.29) | 103.76 (44.11-196.67) | 1.51 (1.53-1.42) | 0.12 (0.05-0.23) | 0.12 (0.05-0.23) | 0.17 (0.05-0.3) |
| Low SDI | 30-34 years | 56.39 (29.42-96.39) | 137.79 (71.45-231.09) | 1.44 (1.43-1.4) | 0.19 (0.1-0.32) | 0.19 (0.1-0.32) | -0.04 (-0.13-0.05) |
| Low SDI | 35-39 years | 77.93 (32.37-138.33) | 176.14 (72.55-312.76) | 1.26 (1.24-1.26) | 0.31 (0.13-0.54) | 0.28 (0.12-0.5) | -0.22 (-0.31--0.13) |
| Low SDI | 40-44 years | 131.28 (67.68-225.62) | 302.01 (155.03-518.47) | 1.3 (1.29-1.3) | 0.66 (0.34-1.13) | 0.59 (0.3-1) | -0.53 (-0.6--0.45) |
| Low SDI | 45-49 years | 188.97 (94.68-351.14) | 391.96 (196.66-727.09) | 1.07 (1.08-1.07) | 1.12 (0.56-2.09) | 0.94 (0.47-1.74) | -0.7 (-0.8--0.6) |
| Low-middle SDI | 15-19 years | 37.19 (12.39-75.62) | 55 (19.62-115.65) | 0.48 (0.58-0.53) | 0.03 (0.01-0.06) | 0.03 (0.01-0.06) | -0.13 (-0.35-0.09) |
| Low-middle SDI | 15-49 years | 1538.59 (1005.91-2364.89) | 3333.46 (2035.56-5209) | 1.17 (1.02-1.2) | 0.28 (0.18-0.43) | 0.33 (0.2-0.51) | 0.61 (0.52-0.71) |
| Low-middle SDI | 20-24 years | 75.63 (35.75-139.31) | 133.38 (66.65-236.51) | 0.76 (0.86-0.7) | 0.07 (0.03-0.13) | 0.08 (0.04-0.14) | 0.09 (-0.12-0.3) |
| Low-middle SDI | 25-29 years | 127.56 (57.24-243.2) | 239.59 (112.95-444.81) | 0.88 (0.97-0.83) | 0.14 (0.06-0.27) | 0.15 (0.07-0.27) | 0.26 (0.06-0.45) |
| Low-middle SDI | 30-34 years | 177.21 (95.69-294.4) | 368.29 (192.76-611.58) | 1.08 (1.01-1.08) | 0.23 (0.13-0.39) | 0.25 (0.13-0.41) | 0.26 (0.13-0.39) |
| Low-middle SDI | 35-39 years | 248.75 (108.59-439.59) | 519.38 (213.18-926.7) | 1.09 (0.96-1.11) | 0.38 (0.17-0.68) | 0.39 (0.16-0.69) | 0.2 (0.08-0.32) |
| Low-middle SDI | 40-44 years | 352.51 (189.61-598.43) | 820.28 (410.85-1409.16) | 1.33 (1.17-1.35) | 0.66 (0.36-1.13) | 0.71 (0.36-1.22) | 0.25 (0.2-0.29) |
| Low-middle SDI | 45-49 years | 519.74 (258.88-986.42) | 1197.54 (612.34-2129.84) | 1.3 (1.37-1.16) | 1.16 (0.58-2.21) | 1.22 (0.62-2.16) | 0.25 (0.13-0.38) |
| Middle SDI | 15-19 years | 48.68 (17.47-96.3) | 39.51 (15.87-78.78) | -0.19 (-0.09--0.18) | 0.03 (0.01-0.05) | 0.02 (0.01-0.04) | -0.67 (-0.78--0.55) |
| Middle SDI | 15-49 years | 2684.83 (1746.87-4082.14) | 5350.6 (3334.77-8021.31) | 0.99 (0.91-0.96) | 0.29 (0.19-0.45) | 0.43 (0.27-0.64) | 1.12 (1.05-1.19) |
| Middle SDI | 20-24 years | 101.15 (51.38-174.21) | 112.84 (57.5-189.85) | 0.12 (0.12-0.09) | 0.06 (0.03-0.1) | 0.06 (0.03-0.11) | 0.21 (0.04-0.38) |
| Middle SDI | 25-29 years | 194.34 (89.73-352.02) | 276.86 (135.29-495.03) | 0.42 (0.51-0.41) | 0.13 (0.06-0.23) | 0.15 (0.07-0.27) | 0.44 (0.27-0.61) |
| Middle SDI | 30-34 years | 308.53 (169.91-503.53) | 521.88 (289.15-839.05) | 0.69 (0.7-0.67) | 0.25 (0.14-0.41) | 0.26 (0.14-0.42) | 0.33 (0.18-0.47) |
| Middle SDI | 35-39 years | 458.16 (200.92-791.52) | 831.78 (361.04-1404.59) | 0.82 (0.8-0.77) | 0.4 (0.18-0.7) | 0.45 (0.2-0.76) | 0.35 (0.21-0.49) |
| Middle SDI | 40-44 years | 648.66 (354.41-1058.39) | 1366.62 (754.02-2207.09) | 1.11 (1.13-1.09) | 0.74 (0.4-1.21) | 0.83 (0.46-1.34) | 0.19 (-0.01-0.38) |
| Middle SDI | 45-49 years | 925.3 (475.13-1664.23) | 2201.12 (1195.59-3726.38) | 1.38 (1.52-1.24) | 1.31 (0.68-2.36) | 1.35 (0.74-2.29) | -0.06 (-0.23-0.1) |
| High-middle SDI | 15-19 years | 13.44 (4.81-25.99) | 7 (3.03-13.27) | -0.48 (-0.37--0.49) | 0.01 (0-0.03) | 0.01 (0-0.02) | -1.36 (-1.49--1.23) |
| High-middle SDI | 15-49 years | 1407.18 (874.25-2194.14) | 3222.04 (1943.44-5069.24) | 1.29 (1.22-1.31) | 0.25 (0.15-0.39) | 0.51 (0.31-0.81) | 2.26 (1.76-2.77) |
| High-middle SDI | 20-24 years | 29.62 (16.04-50.29) | 21.66 (11.3-36.85) | -0.27 (-0.3--0.27) | 0.03 (0.02-0.05) | 0.03 (0.02-0.05) | -0.13 (-0.8-0.53) |
| High-middle SDI | 25-29 years | 65.16 (30.83-119.88) | 84.55 (40.27-151.93) | 0.3 (0.31-0.27) | 0.07 (0.03-0.13) | 0.1 (0.05-0.18) | 2.15 (0.89-3.42) |
| High-middle SDI | 30-34 years | 133.3 (74.54-217.51) | 300.94 (164.5-477.86) | 1.26 (1.21-1.2) | 0.16 (0.09-0.26) | 0.28 (0.15-0.45) | 2.95 (2-3.9) |
| High-middle SDI | 35-39 years | 227.68 (96.63-389.28) | 579.43 (255.52-980.04) | 1.54 (1.64-1.52) | 0.28 (0.12-0.49) | 0.57 (0.25-0.97) | 2.58 (2.21-2.95) |
| High-middle SDI | 40-44 years | 370.26 (195.82-606.74) | 910.39 (488.81-1493.86) | 1.46 (1.5-1.46) | 0.59 (0.31-0.97) | 0.99 (0.53-1.62) | 1.28 (0.88-1.68) |
| High-middle SDI | 45-49 years | 567.72 (296.58-1009.25) | 1318.09 (689.95-2327.25) | 1.32 (1.33-1.31) | 1.15 (0.6-2.04) | 1.36 (0.71-2.4) | 0.3 (-0.24-0.84) |
| High SDI | 15-19 years | 4.78 (1.84-9.3) | 2.78 (1.23-5.14) | -0.42 (-0.33--0.45) | 0.01 (0-0.01) | 0 (0-0.01) | -1.54 (-1.6--1.49) |
| High SDI | 15-49 years | 1736.61 (1041.99-2677.95) | 1961.63 (1204.92-2962.46) | 0.13 (0.16-0.11) | 0.38 (0.23-0.58) | 0.39 (0.24-0.59) | -0.16 (-0.43-0.12) |
| High SDI | 20-24 years | 14.41 (7.49-24.71) | 12.42 (6.42-21.38) | -0.14 (-0.14--0.13) | 0.02 (0.01-0.04) | 0.02 (0.01-0.03) | -0.03 (-0.13-0.07) |
| High SDI | 25-29 years | 52.93 (25.24-95.96) | 50.28 (24.78-87.17) | -0.05 (-0.02--0.09) | 0.07 (0.03-0.13) | 0.07 (0.03-0.12) | 0.39 (0.06-0.71) |
| High SDI | 30-34 years | 148.76 (83.25-237.24) | 144.51 (82.25-225.56) | -0.03 (-0.01--0.05) | 0.21 (0.12-0.33) | 0.19 (0.11-0.29) | -0.34 (-0.69-0.02) |
| High SDI | 35-39 years | 289.62 (127.41-490.03) | 280.74 (128.28-465.1) | -0.03 (0.01--0.05) | 0.43 (0.19-0.72) | 0.36 (0.16-0.59) | -0.95 (-1.18--0.73) |
| High SDI | 40-44 years | 487.01 (258.15-797.53) | 517.39 (284.97-823.96) | 0.06 (0.1-0.03) | 0.77 (0.41-1.26) | 0.68 (0.38-1.09) | -0.85 (-1.12--0.58) |
| High SDI | 45-49 years | 739.09 (385.32-1287.46) | 953.5 (527.12-1606.06) | 0.29 (0.37-0.25) | 1.46 (0.76-2.54) | 1.3 (0.72-2.19) | -0.34 (-0.65--0.02) |
