# Supplementary material for: Global, regional and national burden of Metabolic dysfunction-associated steatotic liver disease in adolescents and adults aged 15–49 years from 1990 to 2021: results from the 2021 Global Burden of Disease study
Source: Front Med (Lausanne). 2025 Jun 25;12:1568211. doi: 10.3389/fmed.2025.1568211 (PMC12237898; doi:10.3389/fmed.2025.1568211)
Supplement: Supplementary file 1 [file Supplementary_file_1.ZIP › Supplementary Table 1 .docx]

**Supplementary Table 1** The incidence cases and rates for MASLD among the adolescents and adults aged 15-49 years from 1990 to 2021

| **location** | **Incidence cases** | | | **Incidence rates** | | |
| --- | --- | --- | --- | --- | --- | --- |
|  | **1990 thousand (95%UI)** | **2021 thousand**  **(95%UI)** | **percentage**  **Change**  **(100%)** | **1990**  **Per 100,000**  **(95%UI)** | **2021**  **Per 100,000**  **(95%UI)** | **EAPC**  **(95% CI)** |
| Andean Latin America | 145.2 (127.28-164.19) | 317.97 (283.64-354.28) | 1.19 | 779.13 (683.02-881.05) | 909.06 (810.91-1012.86) | 0.53 (0.52-0.54) |
| Australasia | 45.85 (40.24-51.57) | 75.42 (66.51-85.1) | 0.64 | 424.93 (372.88-477.9) | 522.3 (460.57-589.31) | 0.74 (0.71-0.77) |
| Caribbean | 143.16 (126.44-161.76) | 208.02 (184.26-233.66) | 0.45 | 783.75 (692.17-885.54) | 868.77 (769.53-975.84) | 0.42 (0.39-0.45) |
| Central Asia | 279.76 (245.26-318.09) | 454.27 (401.46-511.36) | 0.62 | 838.95 (735.48-953.89) | 931.67 (823.36-1048.75) | 0.47 (0.41-0.53) |
| Central Europe | 389.82 (344.52-439.39) | 353.78 (315.19-397.44) | -0.09 | 627.76 (554.82-707.58) | 671.44 (598.19-754.3) | 0.24 (0.23-0.26) |
| Central Latin America | 757.92 (667.79-856.83) | 1390.69 (1228.77-1557.49) | 0.83 | 928.5 (818.09-1049.67) | 1044.64 (923.01-1169.93) | 0.42 (0.4-0.43) |
| Central Sub-Saharan Africa | 164.04 (142.34-187.26) | 474.7 (413.57-536.69) | 1.89 | 671.86 (582.95-766.96) | 728.05 (634.29-823.13) | 0.24 (0.19-0.28) |
| East Asia | 5149.2 (4512.72-5842.09) | 6227.05 (5499.02-7039.54) | 0.21 | 747.5 (655.11-848.09) | 904.43 (798.69-1022.44) | 0.66 (0.46-0.87) |
| Eastern Europe | 674.18 (595.75-761.88) | 635.19 (561.76-716.25) | -0.06 | 611.29 (540.18-690.81) | 660.1 (583.8-744.34) | 0.31 (0.28-0.34) |
| Eastern Sub-Saharan Africa | 602.6 (525.25-682.4) | 1712 (1494.56-1940.87) | 1.84 | 722.43 (629.7-818.11) | 817.59 (713.74-926.89) | 0.41 (0.39-0.43) |
| Global | 19815.35 (17410.85-22363.93) | 35600.57 (31675.9-39968.41) | 0.8 | 731.07 (642.35-825.09) | 901.6 (802.21-1012.22) | 0.72 (0.67-0.77) |
| High-income Asia Pacific | 421.54 (370.4-475) | 366.49 (322.02-415.68) | -0.13 | 454.13 (399.03-511.72) | 468.54 (411.68-531.42) | 0.27 (0.18-0.35) |
| High-income North America | 658.21 (576.41-748.25) | 916.02 (805.35-1030.36) | 0.39 | 441.67 (386.78-502.09) | 543.1 (477.49-610.9) | 0.82 (0.77-0.87) |
| High-middle SDI | 4152.03 (3660.45-4720.21) | 5640.76 (5021.42-6350.92) | 0.36 | 735.64 (648.54-836.31) | 895.96 (797.59-1008.76) | 0.68 (0.58-0.79) |
| High SDI | 2306.68 (2028.5-2602.3) | 3289.26 (2932.05-3694.01) | 0.43 | 500.52 (440.16-564.67) | 654.93 (583.8-735.52) | 1.05 (0.99-1.12) |
| Low-middle SDI | 4296.04 (3775.28-4846.37) | 9628.75 (8515.4-10851.83) | 1.24 | 779.54 (685.05-879.41) | 947.48 (837.92-1067.83) | 0.64 (0.6-0.67) |
| Low SDI | 1598.34 (1396.43-1811.08) | 4607.74 (4036.43-5189.77) | 1.88 | 723.1 (631.75-819.34) | 849.54 (744.21-956.85) | 0.52 (0.49-0.55) |
| Middle SDI | 7443.4 (6539.33-8386.35) | 12405.52 (11066.26-13971.08) | 0.67 | 817.42 (718.13-920.97) | 988.44 (881.73-1113.18) | 0.65 (0.6-0.7) |
| North Africa and Middle East | 2287.77 (2008.91-2569.54) | 5554.19 (4989.54-6154.73) | 1.43 | 1427.42 (1253.43-1603.23) | 1661.28 (1492.39-1840.91) | 0.54 (0.47-0.6) |
| Oceania | 26.88 (23.52-30.45) | 64.23 (56.87-72.91) | 1.39 | 841.19 (736.07-953.04) | 907.88 (803.78-1030.61) | 0.25 (0.21-0.29) |
| South Asia | 3491.02 (3054.96-3959.6) | 8291.26 (7315.94-9429.13) | 1.38 | 659.88 (577.46-748.46) | 823.6 (726.72-936.63) | 0.7 (0.63-0.77) |
| Southeast Asia | 1952.3 (1715.02-2204.47) | 3482.74 (3084.63-3935.19) | 0.78 | 825.16 (724.87-931.74) | 939.24 (831.88-1061.26) | 0.46 (0.43-0.49) |
| Southern Latin America | 111.93 (97.6-127.08) | 194.75 (172.15-220.8) | 0.74 | 457.02 (398.52-518.9) | 561.46 (496.28-636.55) | 0.69 (0.64-0.74) |
| Southern Sub-Saharan Africa | 233.84 (204.5-264.71) | 442.87 (392.7-498.51) | 0.89 | 907.88 (793.97-1027.71) | 1025.86 (909.66-1154.75) | 0.48 (0.45-0.51) |
| Tropical Latin America | 664.94 (588.38-752.83) | 1163.42 (1036.17-1312.02) | 0.75 | 846.85 (749.34-958.79) | 970.9 (864.71-1094.9) | 0.53 (0.5-0.56) |
| Western Europe | 920.5 (809.28-1036.29) | 1086.4 (963.94-1216.59) | 0.18 | 475.91 (418.41-535.78) | 576.3 (511.34-645.37) | 0.69 (0.66-0.72) |
| Western Sub-Saharan Africa | 694.18 (608.4-787.68) | 2188.7 (1917.76-2471.33) | 2.15 | 810.9 (710.69-920.13) | 954.45 (836.3-1077.7) | 0.52 (0.51-0.53) |
