# Supplementary material for: Global, regional and national burden of Metabolic dysfunction-associated steatotic liver disease in adolescents and adults aged 15–49 years from 1990 to 2021: results from the 2021 Global Burden of Disease study
Source: Front Med (Lausanne). 2025 Jun 25;12:1568211. doi: 10.3389/fmed.2025.1568211 (PMC12237898; doi:10.3389/fmed.2025.1568211)
Supplement: Supplementary file 1 [file Supplementary_file_1.ZIP › Supplementary Table 2 .docx]

**Supplementary Table 2** The DALYs cases and rates for MASLD among the adolescents and adults aged 15-49 years from 1990 to 2021

| **location** | **DALYs cases** | | | **DALYs rates** | | |
| --- | --- | --- | --- | --- | --- | --- |
|  | **1990 thousand**  **(95%UI)** | **2021 thousand**  **(95%UI)** | **percentage**  **Change**  **(100%)** | **1990**  **Per 100,000**  **(95%UI)** | **2021**  **Per 100,000**  **(95%UI)** | **EAPC**  **(95% CI)** |
| Andean Latin America | 9.4 (5.64-14.25) | 17.44 (10.56-27.22) | 0.86 | 50.46 (30.27-76.46) | 49.86 (30.19-77.83) | -0.28 (-0.45--0.12) |
| Australasia | 1.15 (0.7-1.7) | 1.92 (1.22-2.8) | 0.67 | 10.62 (6.46-15.76) | 13.28 (8.47-19.41) | 1.01 (0.71-1.32) |
| Caribbean | 5.98 (3.64-9.16) | 9.94 (5.68-15.88) | 0.66 | 32.76 (19.93-50.17) | 41.49 (23.72-66.32) | 0.84 (0.57-1.12) |
| Central Asia | 6.7 (4.26-10.3) | 26.34 (15.49-41.18) | 2.93 | 20.08 (12.77-30.9) | 54.02 (31.77-84.45) | 2.94 (2.46-3.42) |
| Central Europe | 12.57 (7.36-19.94) | 15.99 (9.36-25.39) | 0.27 | 20.25 (11.85-32.11) | 30.34 (17.76-48.18) | 0.3 (-0.08-0.68) |
| Central Latin America | 40.87 (26.31-61.27) | 101.85 (64.02-151.98) | 1.49 | 50.07 (32.23-75.06) | 76.5 (48.09-114.17) | 1.27 (1.09-1.46) |
| Central Sub-Saharan Africa | 3.91 (2.31-6.35) | 10.58 (6-16.7) | 1.71 | 16 (9.46-26.01) | 16.23 (9.21-25.62) | 0.09 (0.02-0.17) |
| East Asia | 42.85 (26.76-65.1) | 31.9 (18.04-50.78) | -0.26 | 6.22 (3.88-9.45) | 4.63 (2.62-7.38) | -0.89 (-1.02--0.76) |
| Eastern Europe | 14.56 (9-22.48) | 104.66 (62.57-163.74) | 6.19 | 13.2 (8.16-20.38) | 108.76 (65.02-170.16) | 6.44 (5.25-7.65) |
| Eastern Sub-Saharan Africa | 10.58 (6.62-16.27) | 29.56 (18.67-46.1) | 1.79 | 12.69 (7.94-19.51) | 14.12 (8.92-22.02) | 0.25 (0.19-0.3) |
| Global | 398.85 (260.59-610.81) | 750.63 (475.83-1124.35) | 0.88 | 14.72 (9.61-22.54) | 19.01 (12.05-28.47) | 0.77 (0.6-0.93) |
| High-income Asia Pacific | 7.92 (4.68-12.64) | 3.84 (2.21-6.41) | -0.52 | 8.53 (5.04-13.62) | 4.91 (2.83-8.2) | -1.84 (-2--1.68) |
| High-income North America | 25.33 (15.79-38.7) | 33.51 (20.73-51.99) | 0.32 | 16.99 (10.59-25.97) | 19.87 (12.29-30.82) | 0.39 (0.26-0.53) |
| High-middle SDI | 69.85 (44.1-106.78) | 157.56 (95.87-244.2) | 1.26 | 12.38 (7.81-18.92) | 25.03 (15.23-38.79) | 2.25 (1.75-2.76) |
| High SDI | 84.83 (52.19-130.1) | 94.7 (59.17-141.45) | 0.12 | 18.41 (11.32-28.23) | 18.86 (11.78-28.16) | -0.2 (-0.45-0.05) |
| Low-middle SDI | 79.03 (51.93-119.36) | 169.27 (103.17-262.03) | 1.14 | 14.34 (9.42-21.66) | 16.66 (10.15-25.78) | 0.56 (0.46-0.66) |
| Low SDI | 27.77 (17.77-43.06) | 63.48 (40.25-95.97) | 1.29 | 12.56 (8.04-19.48) | 11.7 (7.42-17.69) | -0.27 (-0.33--0.21) |
| Middle SDI | 136.83 (91.53-205.53) | 264.84 (167.62-391.68) | 0.94 | 15.03 (10.05-22.57) | 21.1 (13.36-31.21) | 1.02 (0.95-1.09) |
| North Africa and Middle East | 13.79 (8.78-21.04) | 35.48 (21.14-56.88) | 1.57 | 8.6 (5.48-13.13) | 10.61 (6.32-17.01) | 0.73 (0.67-0.78) |
| Oceania | 0.32 (0.19-0.52) | 0.64 (0.38-1.04) | 1 | 10.06 (5.8-16.21) | 9.04 (5.43-14.66) | -0.65 (-0.78--0.52) |
| South Asia | 73 (46.91-111.44) | 144.77 (86.01-226.56) | 0.98 | 13.8 (8.87-21.06) | 14.38 (8.54-22.51) | 0.13 (0-0.26) |
| Southeast Asia | 30.1 (19.12-47.99) | 61.21 (37.53-96.06) | 1.03 | 12.72 (8.08-20.29) | 16.51 (10.12-25.91) | 0.93 (0.84-1.03) |
| Southern Latin America | 6.38 (3.83-10.19) | 6.21 (3.65-9.84) | -0.03 | 26.06 (15.66-41.61) | 17.9 (10.53-28.36) | -0.54 (-0.74--0.35) |
| Southern Sub-Saharan Africa | 5.04 (3.31-7.49) | 11.38 (6.87-17.69) | 1.26 | 19.56 (12.85-29.09) | 26.35 (15.92-40.97) | 0.9 (0.32-1.49) |
| Tropical Latin America | 16.41 (10.72-24.49) | 25.84 (16.23-37.97) | 0.57 | 20.9 (13.65-31.2) | 21.56 (13.55-31.68) | -0.02 (-0.21-0.18) |
| Western Europe | 60.35 (37.09-91.8) | 43.73 (27.84-63.21) | -0.28 | 31.2 (19.17-47.46) | 23.2 (14.77-33.53) | -1.15 (-1.56--0.73) |
| Western Sub-Saharan Africa | 11.65 (6.99-18.33) | 33.85 (19.91-53.56) | 1.91 | 13.6 (8.16-21.42) | 14.76 (8.68-23.36) | 0.46 (0.39-0.53) |
