# Supplementary material for: Global, regional and national burden of Metabolic dysfunction-associated steatotic liver disease in adolescents and adults aged 15–49 years from 1990 to 2021: results from the 2021 Global Burden of Disease study
Source: Front Med (Lausanne). 2025 Jun 25;12:1568211. doi: 10.3389/fmed.2025.1568211 (PMC12237898; doi:10.3389/fmed.2025.1568211)
Supplement: Supplementary file 1 [file Supplementary_file_1.ZIP › Supplementary Table 3 .docx]

**Supplementary Table 3** The deaths cases and rates for MASLD among the adolescents and adults aged 15-49 years from 1990 to 2021

| **location** | **Deaths cases** | | | **Deaths rates** | | |
| --- | --- | --- | --- | --- | --- | --- |
|  | **1990**  **(95%UI)** | **2021**  **(95%UI)** | **percentage**  **Change**  **(100%)** | **1990**  **Per 100,000**  **(95%UI)** | **2021**  **Per 100,000**  **(95%UI)** | **EAPC**  **(95% CI)** |
| Andean Latin America | 184.77 (110-279.55) | 351.32 (211.22-555.68) | 0.9 | 0.99 (0.59-1.5) | 1 (0.6-1.59) | -0.21 (-0.38--0.05) |
| Australasia | 23.34 (13.9-35.16) | 40.18 (25.41-58.9) | 0.72 | 0.22 (0.13-0.33) | 0.28 (0.18-0.41) | 1.11 (0.78-1.44) |
| Caribbean | 118.53 (70.69-183.71) | 201.96 (112.48-330.27) | 0.7 | 0.65 (0.39-1.01) | 0.84 (0.47-1.38) | 0.93 (0.66-1.2) |
| Central Asia | 127.26 (80.65-198.62) | 518.14 (302.89-824.01) | 3.07 | 0.38 (0.24-0.6) | 1.06 (0.62-1.69) | 3.1 (2.6-3.59) |
| Central Europe | 255.67 (145.93-410.6) | 334.27 (196.03-538.94) | 0.31 | 0.41 (0.24-0.66) | 0.63 (0.37-1.02) | 0.34 (-0.06-0.74) |
| Central Latin America | 820.45 (510.54-1252.51) | 2092.12 (1296.13-3082.96) | 1.55 | 1.01 (0.63-1.53) | 1.57 (0.97-2.32) | 1.36 (1.18-1.54) |
| Central Sub-Saharan Africa | 76.3 (44.1-126.17) | 207.17 (115.11-336.53) | 1.72 | 0.31 (0.18-0.52) | 0.32 (0.18-0.52) | 0.11 (0.03-0.18) |
| East Asia | 852.35 (521.04-1306.55) | 655.06 (367.52-1040.78) | -0.23 | 0.12 (0.08-0.19) | 0.1 (0.05-0.15) | -0.77 (-0.9--0.63) |
| Eastern Europe | 292.43 (180.54-455.67) | 2137.26 (1277.05-3367.8) | 6.31 | 0.27 (0.16-0.41) | 2.22 (1.33-3.5) | 6.43 (5.23-7.64) |
| Eastern Sub-Saharan Africa | 207.38 (126.17-325.17) | 575.42 (359.64-900.54) | 1.77 | 0.25 (0.15-0.39) | 0.27 (0.17-0.43) | 0.21 (0.15-0.27) |
| Global | 7919.77 (5125.49-12455.41) | 15107.88 (9519.61-22599.46) | 0.91 | 0.29 (0.19-0.46) | 0.38 (0.24-0.57) | 0.8 (0.63-0.97) |
| High-income Asia Pacific | 164.2 (95.32-268.06) | 80.58 (45.92-136.7) | -0.51 | 0.18 (0.1-0.29) | 0.1 (0.06-0.17) | -1.82 (-1.98--1.66) |
| High-income North America | 510.93 (314.26-788.18) | 691.73 (421.85-1086.17) | 0.35 | 0.34 (0.21-0.53) | 0.41 (0.25-0.64) | 0.47 (0.31-0.63) |
| High-middle SDI | 1407.18 (874.25-2194.14) | 3222.04 (1943.44-5069.24) | 1.29 | 0.25 (0.15-0.39) | 0.51 (0.31-0.81) | 2.26 (1.76-2.77) |
| High SDI | 1736.61 (1041.99-2677.95) | 1961.63 (1204.92-2962.46) | 0.13 | 0.38 (0.23-0.58) | 0.39 (0.24-0.59) | -0.16 (-0.43-0.12) |
| Low-middle SDI | 1538.59 (1005.91-2364.89) | 3333.46 (2035.56-5209) | 1.17 | 0.28 (0.18-0.43) | 0.33 (0.2-0.51) | 0.61 (0.52-0.71) |
| Low SDI | 541.89 (343.85-856.89) | 1224.21 (755.83-1868.18) | 1.26 | 0.25 (0.16-0.39) | 0.23 (0.14-0.34) | -0.31 (-0.37--0.26) |
| Middle SDI | 2684.83 (1746.87-4082.14) | 5350.6 (3334.77-8021.31) | 0.99 | 0.29 (0.19-0.45) | 0.43 (0.27-0.64) | 1.12 (1.05-1.19) |
| North Africa and Middle East | 270.01 (167.67-418.92) | 711.87 (418.19-1156.59) | 1.64 | 0.17 (0.1-0.26) | 0.21 (0.13-0.35) | 0.79 (0.74-0.85) |
| Oceania | 6.14 (3.48-10.15) | 12.44 (7.29-20.25) | 1.03 | 0.19 (0.11-0.32) | 0.18 (0.1-0.29) | -0.57 (-0.7--0.45) |
| South Asia | 1417.53 (915.06-2192.58) | 2833.08 (1670.35-4477.67) | 1 | 0.27 (0.17-0.41) | 0.28 (0.17-0.44) | 0.16 (0.05-0.28) |
| Southeast Asia | 577.64 (369-923.46) | 1221.43 (734.44-1961.19) | 1.11 | 0.24 (0.16-0.39) | 0.33 (0.2-0.53) | 1.09 (0.99-1.19) |
| Southern Latin America | 131.38 (77.6-211.69) | 128.72 (74.34-205.75) | -0.02 | 0.54 (0.32-0.86) | 0.37 (0.21-0.59) | -0.51 (-0.71--0.32) |
| Southern Sub-Saharan Africa | 97.03 (62.85-146.78) | 226.01 (135.67-351.86) | 1.33 | 0.38 (0.24-0.57) | 0.52 (0.31-0.82) | 0.99 (0.45-1.54) |
| Tropical Latin America | 319.31 (204.99-479.57) | 528.82 (326.08-789.04) | 0.66 | 0.41 (0.26-0.61) | 0.44 (0.27-0.66) | 0.15 (-0.06-0.36) |
| Western Europe | 1243.07 (745.99-1915.33) | 913.39 (566.91-1344.21) | -0.27 | 0.64 (0.39-0.99) | 0.48 (0.3-0.71) | -1.07 (-1.5--0.63) |
| Western Sub-Saharan Africa | 224.03 (132.95-358.18) | 646.92 (378.97-1033.06) | 1.89 | 0.26 (0.16-0.42) | 0.28 (0.17-0.45) | 0.45 (0.38-0.53) |
